# Supplementary material for: A quantitative framework reveals ecological drivers of grassland microbial community assembly in response to warming
Source: Nat Commun. 2020 Sep 18;11:4717. doi: 10.1038/s41467-020-18560-z (PMC7501310; doi:10.1038/s41467-020-18560-z)
Supplement: Supplementary file 1 — Supplementary Information [file 41467_2020_18560_MOESM1_ESM.pdf]

Supplementary information

A quantitative framework reveals ecological drivers of grassland microbial community assembly in response to warming

Ning et al.

Daliang Ning, Mengting Yuan, Linwei Wu, Ya Zhang, Xue Guo, Xishu Zhou, Yunfeng Yang, Adam P. Arkin, Mary K. Firestone, and Jizhong Zhou

Supplementary Notes

Supplementary Note 1. Algorithm and parameter optimization for iCAMP

Phylogenetic binning algorithm. In the first step of iCAMP, all taxa were divided into bins according to a phylogenetic signal threshold (d_s = 0.2) within which the phylogenetic signal of microbial niche preference was generally found significant in various environments[1-3]. Three phylogenetic binning algorithms were compared (Supplementary Figure 3a, b, c), based on phylogenetic distances to abundant species, pairwise phylogenetic distances, and phylogenetic tree, respectively. The algorithms do not have substantial difference in principle, thus led to very similar performances (< 9% difference in performance indexes) of iCAMP when applied to simulated communities (Supplementary Figure 1). Under the low- and medium-phylogenetic-signal scenarios, which are more common in the real world, the algorithm based on phylogenetic tree showed slightly higher quantitative precision (up to 3.9% higher, $p < 0.1$) and qualitative performance (up to 8.5% higher) than the other two algorithms (Supplementary Figure 3d, e), and thus the tree-based binning is used for iCAMP. This is probably because the relatedness in phylogenetic tree cannot be fully represented by distances. On the contrary, under the high-phylogenetic-signal scenario, the algorithm based on pairwise phylogenetic distance showed slightly better performance than other algorithms (Supplementary Figure 1f). Considering microbial traits in the real world usually have low or medium phylogenetic signal[4], the tree-based algorithm is more recommended for iCAMP.

Minimal bin size. To maintain statistical power within each bin, a minimal requirement of bin size (minimal taxa number in a bin, n_{min}) should be defined. If a bin has too few taxa, it will be merged into the most relevant bin. However, high n_{min} may make the phylogenetic distances within some bins too large to maintain phylogenetic signal. A series of the values of n_{min} from 6 to 96 were compared for their impacts on iCAMP performance. While quantitative accuracy was not significantly affected, all other indexes, especially quantitative precision, qualitative precision, and sensitivity of iCAMP, were significantly ($p < 0.05$) influenced by n_{min}, with the best performance at n_{min} = 24 (Supplementary Figure 4a, b, c). To explore the reason, the phylogenetic signal within each bin was analyzed by Mantel tests between phylogenetic distance and niche difference under different n_{min}. When n_{min} = 24, the bins with significant (one-tail $p < 0.05$ and R > 0.10, since R <

0.10 is usually regarded as negligible effect size[5]) phylogenetic signal reached the highest relative abundance (Supplementary Figure 4d, e, f). When other n_{min} values showed the same relative abundance of significant bins (e.g. n_{min} = 48 and 96 in Supplementary Figure 4e, f), n_{min} = 24 led to higher effect size, measured as average R value of within-bin phylogenetic signal. Thus, n_{min} could be determined according to within-bin phylogenetic signal, i.e. higher relative abundance of bins with significant phylogenetic and higher R value of within-bin phylogenetic signal. In empirical studies, if key environmental variables are available, the function 'dniche' and 'ps.bin' in iCAMP package can be used to calculate within-bin phylogenetic signal to determine n_{min}. If key environmental variables are unknown or unmeasured, n_{min} can be determined in an indirect way: (i) estimating stochasticity level with other approaches, such as phylogenetic normalized stochasticity ratio (pNST), which had better quantitative performance in the simulated communities (Fig. 2a-c); (ii) then, testing different n_{min} values and choosing the one with estimated stochasticity similar to pNST and/or other approaches.

Phylogenetic metrics. To identify the impacts of selection, the phylogenetic metric βNTI (beta Nearest Taxon Index) has been widely used in recent studies[2,3,6-10], mainly because βNTI is based on βMNTD (beta Mean Nearest Taxon Distance) of which the values are generally within the phylogenetic signal threshold[7,10], and thus it is better than other phylogenetic metrics to reflect niche preference dissimilarity. However, in iCAMP, the phylogenetic distances in each bin are mostly within the phylogenetic signal threshold, thus, another phylogenetic metric βNRI (beta Net Relatedness Index) based on βMPD (beta Mean Pairwise Distance) is also applicable. When applied to the simulated communities, βNRI resulted in obviously ($p < 0.01$) higher quantitative precision (13.8-17.3% higher) and qualitative performance (2.7-28.6% higher) of iCAMP than βNTI did under medium- and high-phylogenetic-signal scenarios (Supplementary Figure 5b, c), but only slightly higher quantitative precision (3% higher) than βNTI under low-phylogenetic scenario (Supplementary Figure 5a). While βNTI only counts the distance of each taxon to its nearest relative, βNRI counts distance of each taxon to all other taxa, and hence it includes more information than βNTI. The higher the across-tree phylogenetic signal is, the more useful information βNRI can count in than βNTI. Therefore, the advantage of βNRI in iCAMP is more obvious under scenarios with higher phylogenetic signal. Accordingly, βNRI is preferred in iCAMP.

Randomization range in null models. Besides phylogenetic metrics, the null model algorithm is also critical in the second step of iCAMP. As in QPEN, the phylogenetic null model is utilized to infer selection, and taxonomic null model is used to further identify dispersal limitation and homogenizing dispersal[3,11]. For both phylogenetic and taxonomic null models, the randomization can be performed within each bin or across taxa in all bins, and thus there are four possible combinations (Supplementary Figure 6). In the four combinations, within-bin randomization in phylogenetic null model plus across-bin randomization in taxonomic null model provided higher quantitative precision and qualitative performance (1.6-103% higher, $p < 0.05$) of iCAMP than the other options in all scenarios (Supplementary Figure 6), especially under low-phylogenetic-signal scenario (Supplementary Figure 6a). The best option using within-bin randomization in phylogenetic null model should be mainly due to significant phylogenetic signal within bin rather than across bins, which is important for using βNRI to infer selection. The better performance of across-bin randomization in taxonomic null model is reasonable considering that the taxonomic null model analysis is used to infer neutral dispersal process, which is not species-specific but influences taxa across all bins in probability as long as under the same metacommunity. Therefore, for iCAMP, βNRI should be calculated based on within-bin randomization, and RC should be estimated based on across-bin randomization.

Randomization times. The null model analysis needs enough randomization times to estimate the distribution of the null values of phylogenetic/taxonomic dissimilarity. Low randomization times cannot provide reproducible results, but high randomization times cost more computational resources and/or time. Randomization times ranging from 25 to 5,000 were used for iCAMP analysis of simulated communities, and the quantitative and qualitative results of iCAMP were compared with the iCAMP result from 60,000 randomization. When the randomization times are not less than 200, deviation of quantitative results and the error rate of qualitative results were less than 0.05 (down to zero) and became relative stable (no significant change in mean and deviation) as randomization times increased (Supplementary Figure 7). We also tested different randomization times for QPEN using simulated data. When randomization times are larger than 200, QPEN results showed small deviation (< 6.7%) from null expectation from 60,000 randomizations, with interquartile range (IQR) equal to 0%. When applied to the empirical data,

iCAMP and QPEN with 1,000 randomizations were repeated for 3 times. iCAMP can estimate the relative importance of each process in the 780 pairwise comparisons among the 40 samples. The standard deviation of these tests ranged from 0 to 6.8% (≤8.8% or ≤3.9% if randomizing 200 or 3,000 times, respectively), with IQR values from 0.03% to 1.02% (≤1.5% or ≤0.60% if randomizing 200 or 3,000 times) for different processes. QPEN can identify one dominating process for each of the 780 pairwise comparisons, for which only 1.9% (4.7% or 1.4% if randomizing 200 or 3,000 times) are different among the 3 times. Altogether, the commonly used randomization times (1,000) can provide adequate reproducibility for iCAMP and QPEN analysis, and 200 times may also be acceptable for relatively small data (e.g. less than 2,000 OTUs).

Reducing the taxa number. Besides randomization times, a large taxa number can cause quadratic increase of computational resource demand and time cost for phylogenetic null model analysis, making iCAMP and QPEN much less feasible for very large dataset (e.g. over 100,000 OTUs). In addition, the taxa with low relative abundances may bring more technical noise, for example, in the OTU tables from amplicon sequencing[12-14]. Thus, large data may need to be reduced before the iCAMP analysis. Three methods were compared, including classic rarefaction (rarefaction), cutting based on average relative abundance across samples (average abundance cut), and cutting based on cumulative abundance in each sample (cumulative abundance cut). Their performance was evaluated according to the quantitative deviation and qualitative error rate of iCAMP using the reduced OTU table compared to results from original OTU table. Rarefaction led to obvious overestimation of drift (Supplementary Figure 8), and the error rate of qualitative estimation can be larger than 90% even though over 70% species were still remained (e.g. qualitative in Supplementary Figure 8a). This is probably because rarefaction itself is a random sub-sampling process bringing more artificial stochasticity, and also removes much more sequences than other methods to reduce taxa number. The other two methods resulted in similar performance of iCAMP (Supplementary Figure 8). After cumulative abundance cut, quantitative deviation was usually lower than 10% when taxa number was reduced to a half of the original number, but can be up to 87% (e.g. for homogeneous selection in Supplementary Figure 8b) at 30% of the original taxa number. After abundance cut, qualitative error rates were generally more than 10%, in some cases up to 60%. Altogether, to investigate assembly mechanism, sequencing should be deep enough with high coverage to minimize the negative impact of random sampling.

Reducing taxa number can significantly change the estimation, and thus it is not advisable, except necessary denoising, i.e. removing unreliable technical noise (e.g. removing global singleton)[12]. Our program applies parallel computation, matrix-based algorithm, and 'big memory'[15] (efficiently utilizing hard disk as memory) to make iCAMP feasible for a relative large dataset (10,000-40,000 OTUs) on a desktop computer or a small server. For a very large dataset, high performance computing can help, if not feasible, the taxa number can be reduced by cumulative abundance cut before iCAMP analysis. Although the quantitative results could be acceptable, the qualitative results are not reliable.

Null model significance testing indexes. βNRI is a standard effect size, of which the threshold (i.e. 1.96 or 2) for significant difference is derived based on normal distribution of null model values. When the null distribution is not normal, βNRI has risk to misestimate the significance. Therefore, direct test based on null distribution should be preferred for null model significance test[16]. Accordingly, an alternative significance testing index for iCAMP is defined as a signed nonparametric one-tail confidence level, and the operator for each process can be calculated based on the confidence index, as showed in following equations. If the null model values follow normal distribution, the βNRI threshold 1.96 is equivalent to the confidence threshold 0.975.

$$\mathrm{CbMPD}_{uvk} = \begin{cases} \mathrm{Pr}_{1uvk} = \mathrm{Pr}(\beta\mathrm{MPD}_{\mathrm{null}uvk} < \beta\mathrm{MPD}_{uvk}) & \mathrm{Pr}_{1uvk} \geq \mathrm{Pr}_{2uvk} \\ -\mathrm{Pr}_{2uvk} = \mathrm{Pr}(\beta\mathrm{MPD}_{\mathrm{null}uvk} > \beta\mathrm{MPD}_{uvk}) & \mathrm{Pr}_{1uvk} < \mathrm{Pr}_{2uvk} \end{cases} \quad (1)$$

$$\mathrm{CBray}_{uvk} = \begin{cases} \mathrm{Pr}_{3uvk} = \mathrm{Pr}(\mathrm{BC}_{\mathrm{null}uvk} < \mathrm{BC}_{uvk}) & \mathrm{Pr}_{3uvk} \geq \mathrm{Pr}_{4uvk} \\ -\mathrm{Pr}_{4uvk} = \mathrm{Pr}(\mathrm{BC}_{\mathrm{null}uvk} > \mathrm{BC}_{uvk}) & \mathrm{Pr}_{3uvk} < \mathrm{Pr}_{4uvk} \end{cases} \quad (2)$$

$$W_{\mathrm{HeS}uvk} = \begin{cases} 1 & \mathrm{CbMPD}_{uvk} > 0.975 \\ 0 & \text{else} \end{cases} \quad (3)$$

$$W_{\mathrm{HoS}uvk} = \begin{cases} 1 & \mathrm{CbMPD}_{uvk} < -0.975 \\ 0 & \text{else} \end{cases} \quad (4)$$

$$W_{\mathrm{DL}uvk} = \begin{cases} 1 & |\mathrm{CbMPD}_{uvk}| \leq 0.975 \text{ and } \mathrm{CBray}_{uvk} > 0.975 \\ 0 & \text{else} \end{cases} \quad (5)$$

$$W_{\mathrm{HD}uvk} = \begin{cases} 1 & |\mathrm{CbMPD}_{uvk}| \leq 1.96 \text{ and } \mathrm{CBray}_{uvk} < -0.975 \\ 0 & \text{else} \end{cases} \quad (6)$$

$$W_{\mathrm{DR}uvk} = \begin{cases} 1 & |\mathrm{CbMPD}_{uvk}| \le 1.96 \text{ and } |\mathrm{CBray}_{uvk}| \le 0.975 \\ 0 & \text{else} \end{cases} \quad (7)$$

CbMPD_{uvk} Confidence index based on βMPD of Bin *k* between community *u* and *v*.

CBray_{uvk} Confidence index based on Bray-Curtis dissimilarity of bin k between community *u* and *v*.

$\beta\mathrm{MPD}_{uvk}$ Observed beta mean pairwise distance of Bin *k* between community *u* and *v*, and $\beta\mathrm{MPD}_{\mathrm{null}uvk}$ is the βMPD of the null communities randomized according to a null model.

BC_{uvk} Observed Bray-Curtis dissimilarity, and $\mathrm{BC}_{\mathrm{null}\,uvk}$ represents corresponding null values.

Pr_{1uvk}, Pr_{2uvk} Probability (percentage) of null βMPD values lower or higher than the observed βMPD, respectively.

Pr_{3uvk}, Pr_{4uvk} Probability (percentage) of null Bray-Curtis values lower or higher than the observed value, respectively.

$W_{\mathrm{HeS}uvk}$ Operator for heterogeneous selection, to count whether the turnover of the kth phylogenetic bin (Bin *k*) between community *u* and *v* governed by heterogeneous selection. $W_{\mathrm{HoS}uvk}$, $W_{\mathrm{DL}uvk}$, $W_{\mathrm{HD}uvk}$, and $W_{\mathrm{DR}uvk}$ are analogous operators for homogeneous selection, dispersal limitation, homogenizing dispersal, or ‘drift’, respectively.

Based on the confidence index, iCAMP was performed for all our simulated situations and the empirical data. The performance indexes of iCAMP on simulated data was nearly the same as using βNRI and RC (Supplementary Figure 17a, b), with Pearson and concordance correlation coefficients larger than 0.99, Cohen’s *d* lower than 0.11 (negligible difference), and Wilcoxon *p* larger than 0.93. The estimated process importance at community level (P_τ) and bin level ($P_{\tau k}$) were highly similar to those based on βNRI and RC, in both simulated and empirical datasets (Supplementary Figure 17c, d). Considering their performance, popularity, and relevance to the previous approach (QPEN), we mainly used βNRI and RC rather than the confidence index for iCAMP analysis in this study. However, since microbial communities are so diverse and complicated, the confidence index can be a preferred choice when the null model simulated

distributions are not normal. Accordingly, iCAMP package provides a function ‘null.norm’ for normality test of null values and a function ‘change.sigindex’ for quickly switching between different indexes for null model significance test.

Supplementary Note 2. Robustness to complex assembly mechanisms in each bin

iCAMP only identifies the predominate process controlling a turnover of a bin between two samples. Given the uncertainty in determining phylogenetic signal threshold (d_s) and the necessary to merge small bins into their relatives, it is highly possible that the members in the bin are actually governed by different processes. This complexity raises the risk of misestimating the relative importance of different processes at either bin or community level. To test the robustness of iCAMP to such noises, we simulated communities with bins of different sizes and phylogenetic distance thresholds, but used the same binning setting in iCAMP analysis. Consequently, many bins estimated by iCAMP were actually governed by two or more processes, so called complex bins. The mean ratio and relative abundance of these complex bins (governed by two or more processes) ranged from 0% to 68% under different simulation settings, and up to 100% in some situations (Supplementary Figure 10a-c). The performance of iCAMP became worse as the ratio of complex bins increased, especially in precision and sensitivity under low-phylogenetic-signal scenario (Supplementary Figure 10d, g), and usually worse at bin (Supplementary Figure 10g-i) than community level (Supplementary Figure 10d-f), but it appears that they still better than QPEN (Supplementary Figure 10j-l). In addition, iCAMP had quantitative accuracy and precision higher than 0.98 and 0.68 (Supplementary Figure 10d-i), respectively, at both community and bin level, demonstrating its robustness to noises in bin determination.

Selection can include distinct forces. When abiotic filtering generally selects for phylogenetic relatives in similar environments, competitive exclusion can be strong among the relatives sharing similar niche preference. Thus, abiotic filtering and biotic competition could lead to opposite patterns, significantly decreasing the performance of null-model-based approach[17]. To investigate this issue, we simulated selection-controlled species under different ratios of abiotic filtering and biotic competition and performed iCAMP analysis. When competition contributes less portion than filtering, iCAMP remains relative high performance (index values > 0.7) at both community (Supplementary Figure 11a-c) and bin level (Supplementary Figure 11d-f), indicating the robustness of iCAMP. As competition becomes stronger (>50%) than filtering, iCAMP precision and sensitivity decreases down to 0.5 and 0.6, respectively, although accuracy and specificity still remain higher than 0.8 (Supplementary Figure 11a-f), suggesting some degree of robustness. Regardless of the inference from competition, iCAMP had higher performance than QPEN

(Supplementary Figure 11g-i) in all simulated datasets. Collectively, these results indicate that disentangling abiotic and biotic forces is an important direction for further development.

Supplementary Note 3. Cross-bin selection.

Cross-bin selection can play an essential role in community assembly. For instance, intense competition between different bins can lead to significantly different abundances, thus shape the community structure. Since iCAMP is built on within-bin beta diversity, an intuitive concern is whether iCAMP can capture important cross-bin selection. We hypothesize that a strong cross-bin selection will not only cause between-bin difference or similarity, but also result in obvious within-bin difference detectable by iCAMP.

This hypothesis is theoretically reasonable. A bin in iCAMP is not a small group of species with almost the same niche preference or traits. On the contrary, because iCAMP binning means to ensure adequate within-bin phylogenetic signal (Supplementary Figure 4), each bin generally consists of various species from different genera or families, with obvious differences in niche preference and key traits. Thus, if under strong competition or facilitative interactions from other bins or strong filtering pressure across bins, the members in the same bin should have different responses that are different from random patterns. Therefore, an important cross-bin selection force should be detectable by iCAMP.

The hypothesis is also supported by the fact that significant microbial phylogenetic signal is widely observed within a relatively short phylogenetic distance threshold[1,2,9,10]. If the important cross-bin selection could not lead to obvious within-bin (i.e. within the threshold) difference, the cross-bin selection should be less influential than within-bin selection or only important in some special cases; otherwise, it contradicts to the fact. In other words, the cross-bin selection is either detectable or generally not so important.

In addition, the hypothesis is further supported by the results from our simulated communities. In the simulation model, competition and filtering were simulated across the species pool rather than any specific bin(s). Under all scenarios, cross-bin competition and/or filtering across bins are very common in the situations with relatively high ratio of selection. We even simulated some situations with actual bins larger than estimated bins in iCAMP (see the minimal size of actual bin = 48 in Supplementary Figure 10), leading to strong selection across different estimated bins in iCAMP.

Nevertheless, iCAMP showed excellent quantitative performance in simulated communities, demonstrating the ability to cover the impact of cross-bin selection.

Collectively, iCAMP is generally able to capture important cross-bin selection. But in some special cases when an important cross-bin selection does not lead to detectable within-bin difference, iCAMP might underestimate the importance of selection. The challenge can be solved by further integrating functional genes-based diversity and network analyses with iCAMP.

Supplementary Figures

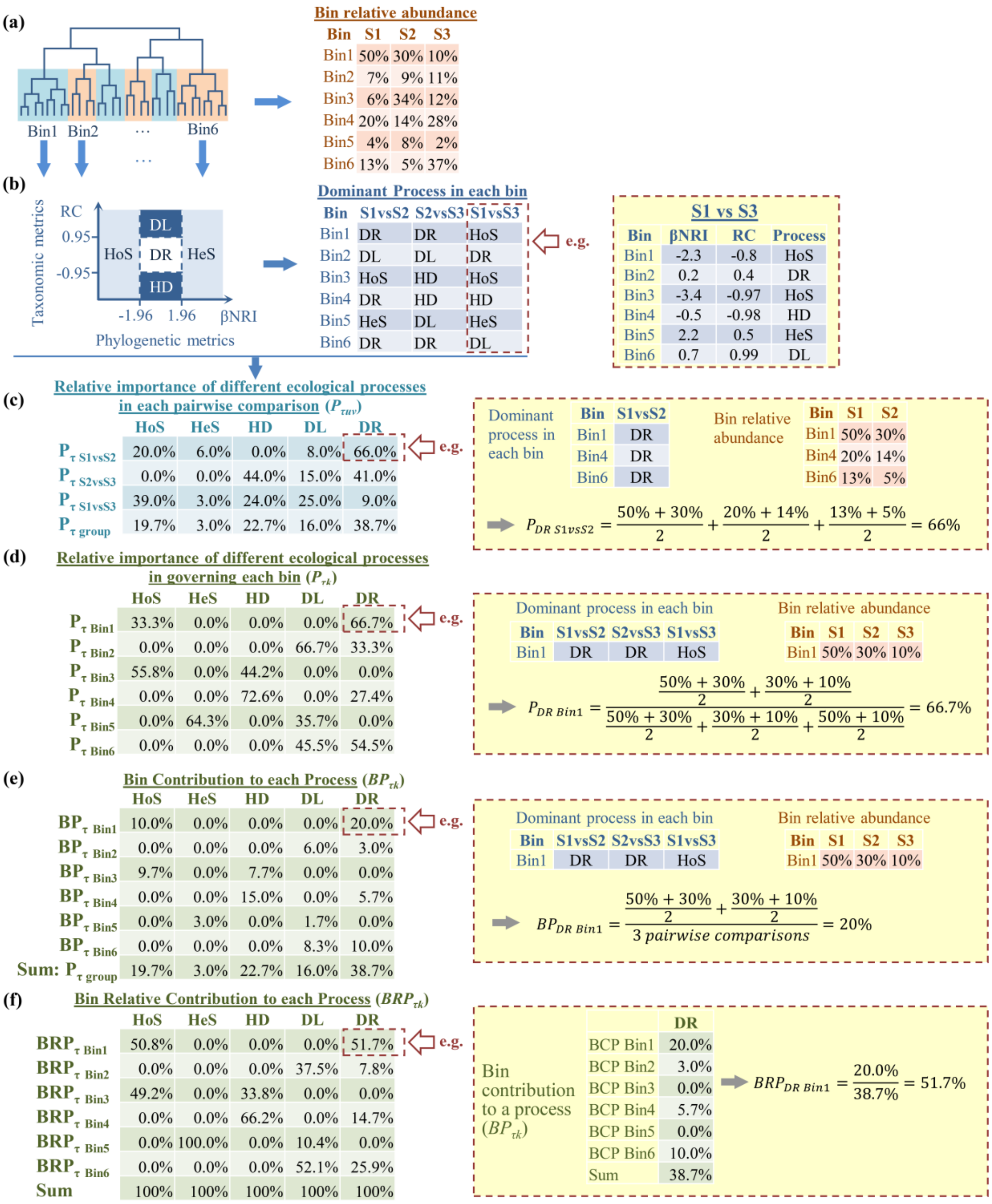

Supplementary Figure 1. A simple example of iCAMP analysis. (a) Phylogenetic binning based on phylogenetic tree. **(b)** Identifying an ecological process governing the turnover of each bin (Eq. 1-10). **(c)** Relative importance of different ecological processes in governing each pairwise community turnover (Eq. 12, 13). A community turnover means the dissimilarity/similarity found

in a pairwise comparison between two communities or samples. **(d)** Relative importance of different ecological processes in governing each bin (Eq. 11). **(e)** Contribution of each bin to each process (Eq. 14). **(f)** Relative contribution of each bin to each process (Eq. 15). Boxes with dashed lines in panel (**c-f**) showed detailed calculation examples. Bin1 to Bin6, phylogenetic bin IDs. S1, S2, S3, sample IDs. HoS, homogeneous selection; HeS, heterogeneous selection; HD, homogenizing dispersal; DL, dispersal limitation; DR, 'drift' including stochastic drift, diversification, weak selection and/or weak dispersal. βNRI, beta net relatedness index calculated from beta mean pairwise distance (βMPD); RC, modified Raup-Crick metrics based on Bray-Curtis dissimilarity.

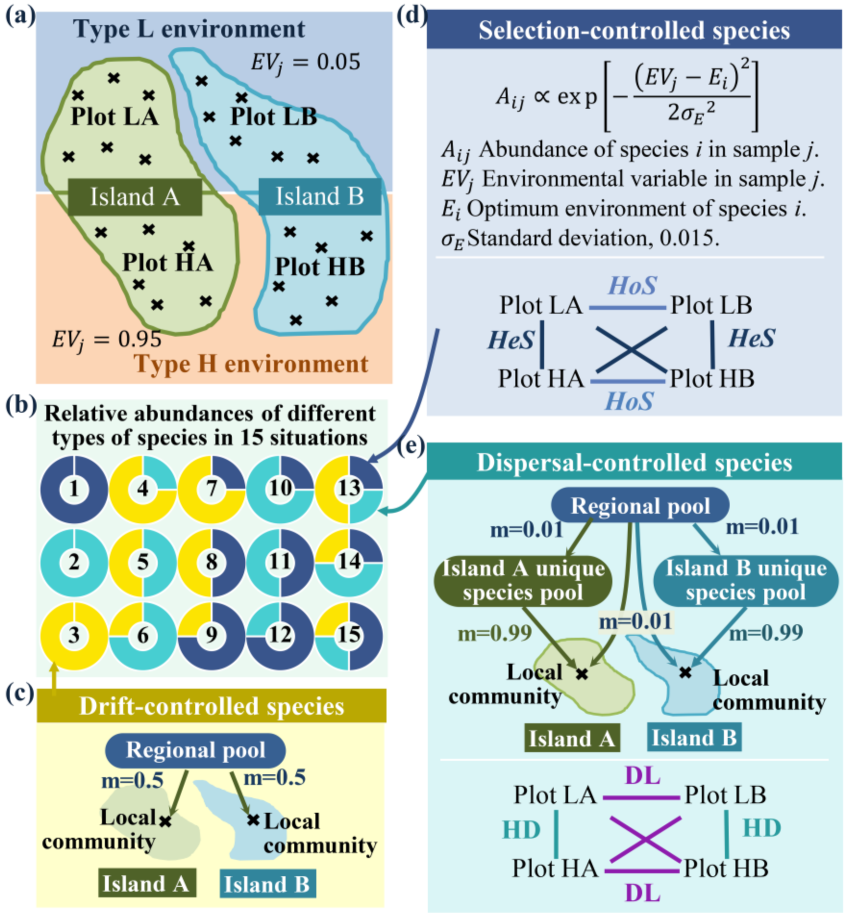

Supplementary Figure 2. Illustration of the basic settings of the simulated communities. (a) Predefined locations of sampling plots (LA, LB, HA, HB) and local communities (black cross) in two islands (A, B) and two types of environments (L, e.g., low temperature; H, e.g., high temperature). **(b)** Predefined relative abundances of species controlled by various processes under 15 different situations. Livid, selection-controlled species; turquoise, dispersal-controlled species; dark yellow, drift-controlled species. These 15 situations were simulated under each of the three scenarios, i.e. low, medium, and high phylogenetic signal of the key traits. **(c)** Immigration setting to simulate species controlled by drift without too high or low dispersal rate. *m* is dispersal rate in neutral theory model, which is the probability that a dead individual will be replaced by an individual immigrating from the regional pool rather than by a local individual. Since the dispersal ($m = 0.5$) is neither limited ($m \rightarrow 0$) nor homogenizing ($m \rightarrow 1$), the turnovers of these species are all controlled by drift. **(d)** Relative abundance of selection-controlled species determined by the key trait (E_i). For selection-controlled species, the turnovers between plots under the same environments (Plot LA vs LB and plot HA vs HB) is governed by homogeneous selection (HoS), and those between different environments (LA vs HA, LA vs HB, LB vs HA, and LB vs HB) is governed by heterogeneous selection (HeS). **(e)** Immigration setting to simulate species controlled by dispersal and the dominant processes between different plots. For dispersal-controlled species, the turnover within each island (LA vs HA, LB vs HB) is controlled by homogenizing dispersal (HD), and their turnover between different islands (LA vs LB, LA vs HB, HA vs HB, and HA vs LB) is controlled by dispersal limitation.

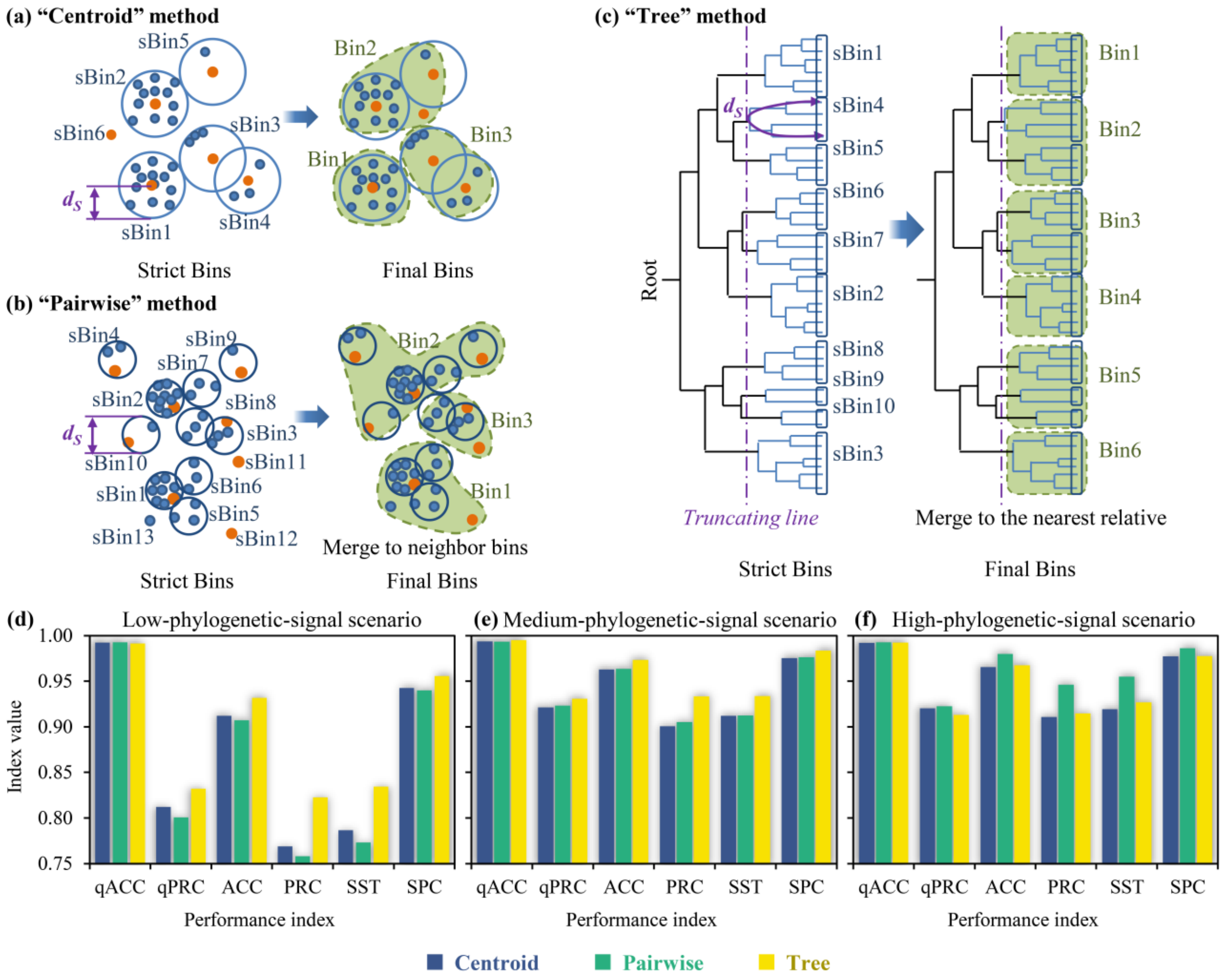

Supplementary Figure 3. Comparison of three methods for phylogenetic binning in iCAMP. (a) Binning based on phylogenetic distance to abundant species ('**centroid**' method). In a strict bin (sBin1-6), all distances to the core species (orange dot, the most abundant species) are shorter than the phylogenetic signal threshold (d_s). To ensure enough statistical power, bins with too few species are combined with the nearest neighbor(s) until all final bins (Bin1-3) reach the minimal requirement (n_{min}, set as 6 in this figure). **(b)** Binning based on pairwise phylogenetic distance ('**pairwise**' method). In a strict bin (sBin1-13), all pairwise distances are shorter than d_s. Then, small bins are combined with the nearest neighbor(s) to form final bins with species no less than n_{min}. **(c)** Binning based on phylogenetic tree ('**tree**' method). In a strict bin, all species have the same ancestor after the truncating point and all pairwise phylogenetic distances are shorter than d_s. Then, small bins are merged to the nearest relative(s) to form final bins. **(d)** Performance of iCAMP using different phylogenetic binning methods under low phylogenetic signal. **(e)** medium phylogenetic signal, and **(f)** high phylogenetic signal. Performance indexes include quantitative accuracy (qACC) and precision (qPRC), qualitative accuracy (ACC), precision (PRC), sensitivity (SST), and specificity (SPC). Source data are provided as a Source Data file.

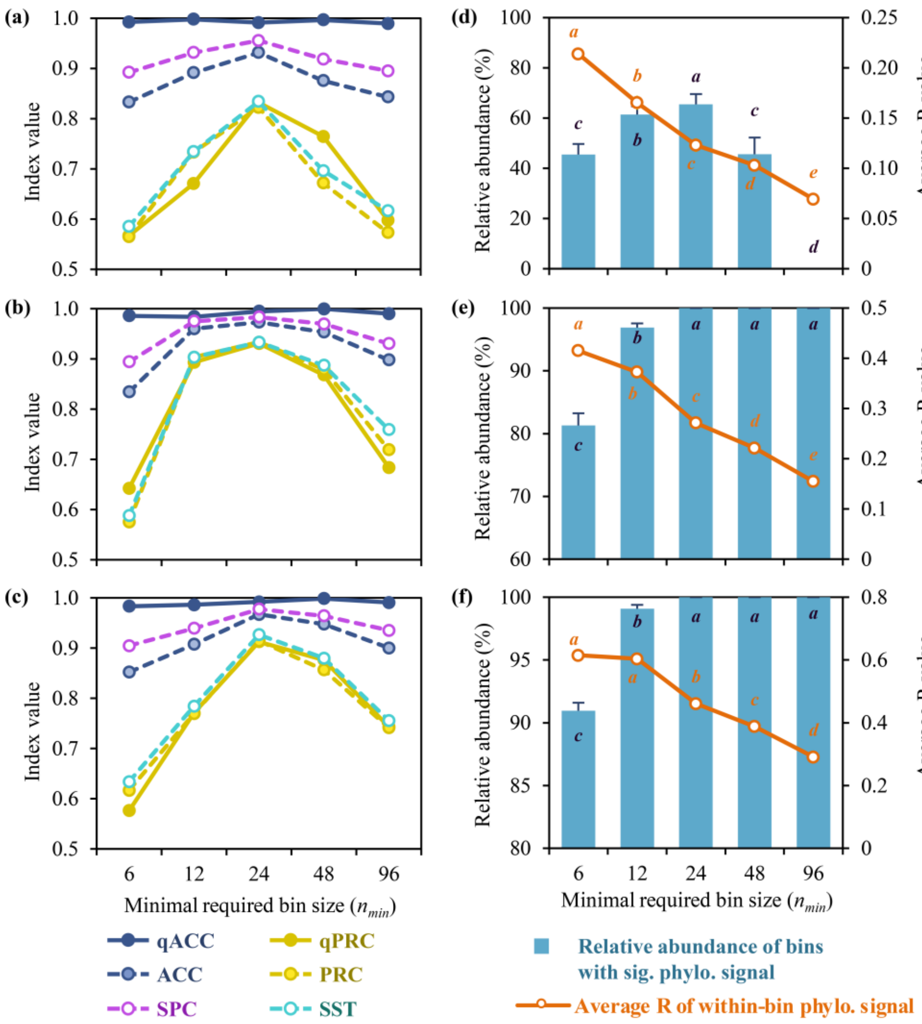

Supplementary Figure 4. Effects of minimum required bin sizes (*n_{min}*) on iCAMP performance and within-bin phylogenetic signal. (a) Performance of iCAMP using different n_{min} under low-phylogenetic-signal scenario, **(b)** medium-phylogenetic-signal scenario, and **(c)** high-phylogenetic-signal scenario. **(d)** Evaluation of within-bin phylogenetic signal when different n_{min} under low-phylogenetic-signal scenario is used, **(e)** medium-phylogenetic-signal scenario, and **(f)** high-phylogenetic-signal scenario. Here, within-bin phylogenetic signal means correlation between the pairwise phylogenetic distances and niche preference differences among species within the same bin. Within-bin phylogenetic signal is evaluated by relative abundance of bins with significant phylogenetic signal and average R value of all within-bin Mantel test. Performance indexes are abbreviated as Supplementary Figure 3. In a bin, the phylogenetic signal is regarded as significant if R > 0.10 (R < 0.10 is usually regarded as negligible effect size[5]) and one-tail p < 0.05 in Mantel test between phylogenetic distances and niche preference difference (key trait difference). (**a-c**) n = 4,140 comparisons = 276 comparisons among 24 biologically independent samples in each of 15 situations. (**d-f**) Data are presented as mean values ± SEM. Error bars indicate standard error of the relative abundances under different situations in each scenario (n = 15 independent situations), and different letters represent significant difference (p < 0.05 based on two-side 1,000 times bootstrapping test, see Source Data file for exact p values). Source data are provided as a Source Data file.

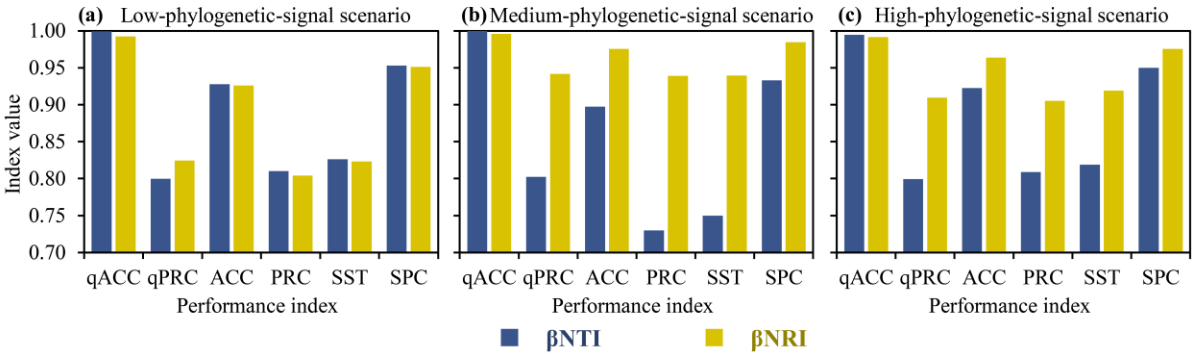

Supplementary Figure 5. Effects of phylogenetic metrics on iCAMP performances under different simulation scenarios. (a) Low-phylogenetic-signal scenario; **(b)** Medium-phylogenetic-signal scenario; **(c)** High-phylogenetic-signal scenario. βNTI, livid; βNRI, dark yellow. Performance indexes are abbreviated as Supplementary Figure 3. Source data are provided as a Source Data file.

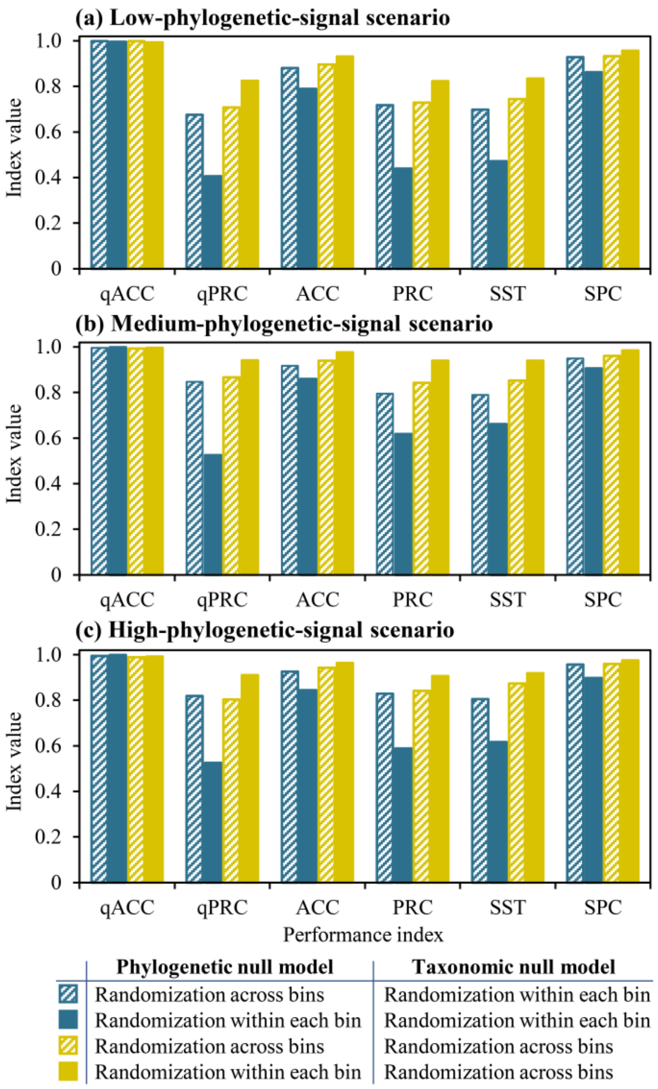

Supplementary Figure 6. Effects of randomization range in phylogenetic and taxonomic null models on iCAMP performances. **(a)** Low-phylogenetic-signal scenario; **(b)** Medium-phylogenetic-signal scenario; **(c)** High-phylogenetic-signal scenario. Diagonal, randomization across bins in phylogenetic null model; filled, randomization within each bin in phylogenetic null model; dark teal, randomization within each bin in taxonomic null model; dark yellow, randomization across bins in taxonomic null model. Performance indices are abbreviated as Supplementary Figure 3. Source data are provided as a Source Data file.

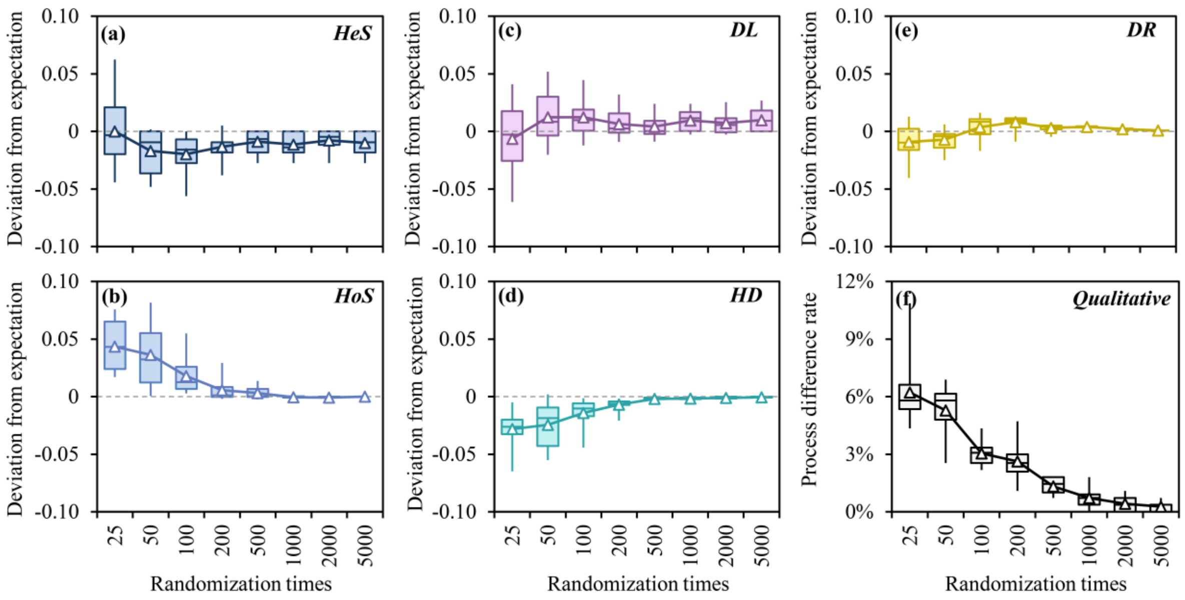

Supplementary Figure 7. Effects of randomization times on the reproducibility of quantitative and qualitative results from iCAMP. (a) Deviation of the estimated relative importance of HeS from expectation under different randomization times. **(b)** Deviation of HoS. **(c)** Deviation of DL. **(d)** Deviation of HD. **(e)** Deviation of DR. **(f)** The rate of dominant processes estimated differently from expectation under different randomization times. iCAMP was applied to simulated communities under the 12th situation (75% selection, 25% dispersal, Supplementary Table 1) in low-phylogenetic-signal scenario. Deviation from expectation was calculated as the difference of the estimated relative importance of a process from the result after 60,000-time randomizations. Process difference rate, percentage of the estimated dominated processes which are different from the estimation after 60,000-time randomizations. The box plots are based on results from iCAMP repeated for 12 times; top of box is 75th percentile; bottom of box is 25th percentile; center bar is median; whiskers show the maximum and minimum values; triangle is mean value. Source data are provided as a Source Data file.

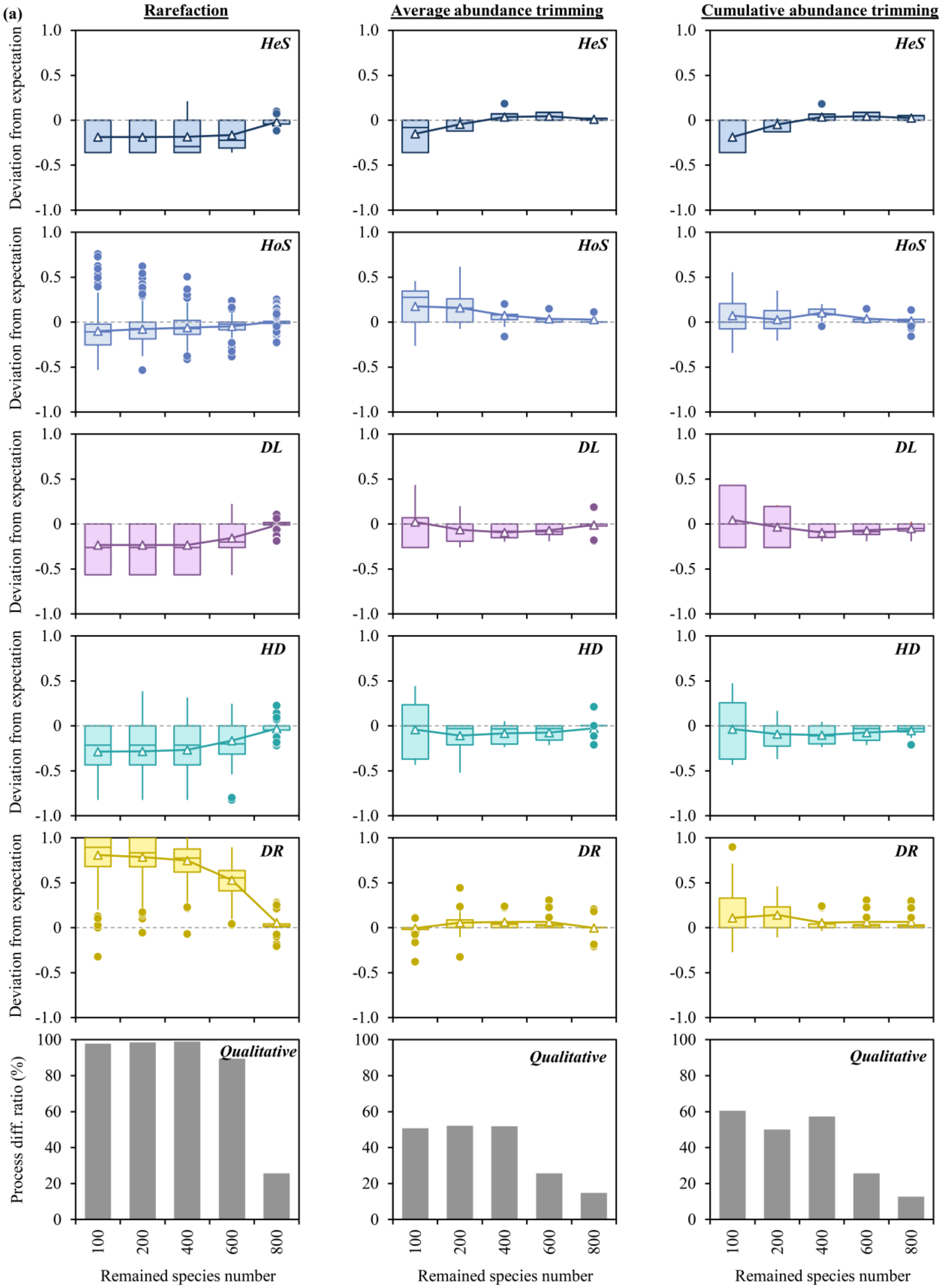
(a)
Rarefaction
Average abundance trimming
Cumulative abundance trimming
HeS
HoS
DL
HD
DR
Qualitative
Deviation from expectation
Process diff. ratio (%)
Remained species number

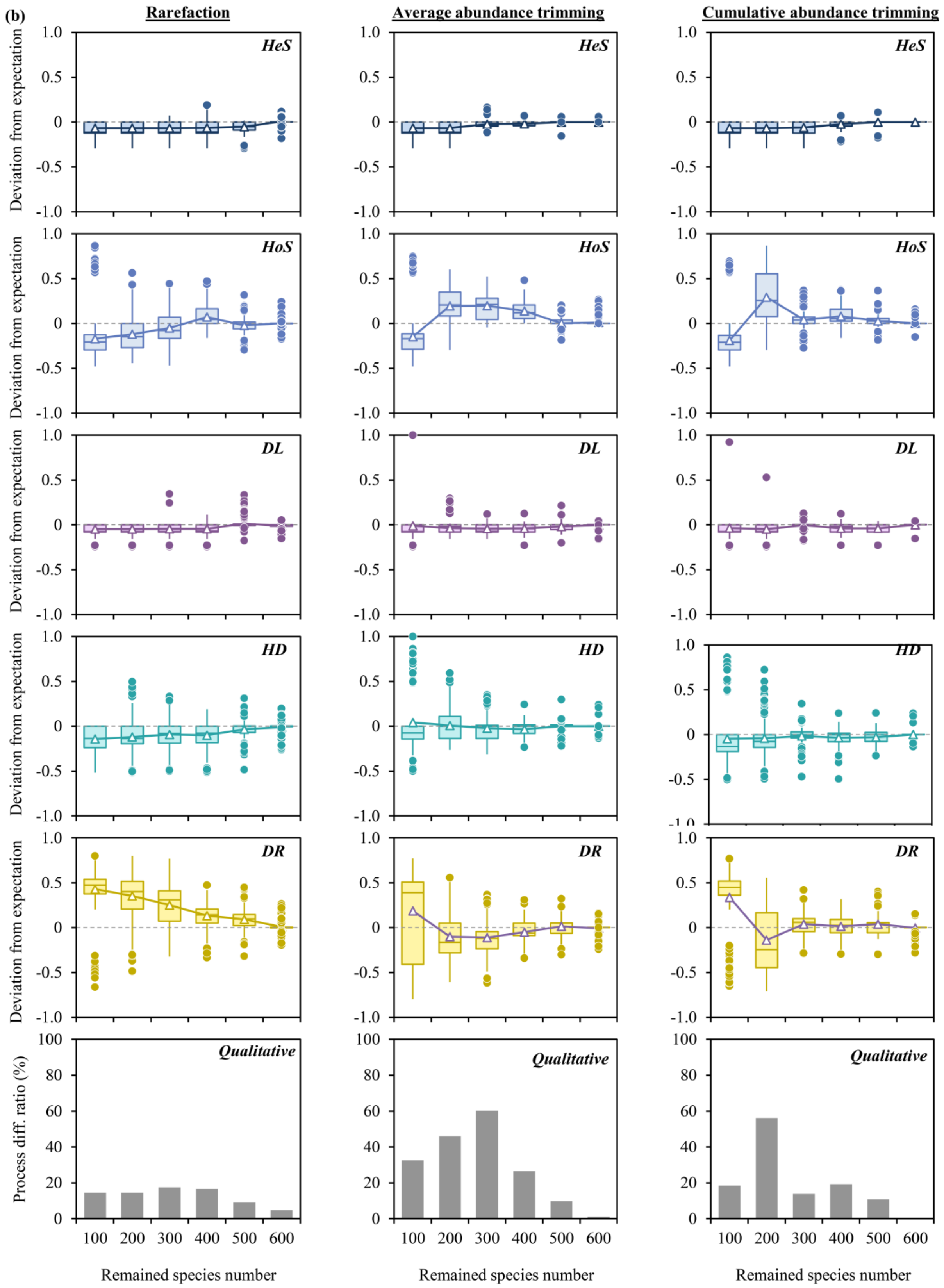

Supplementary Figure 8. Effects of taxa number reduction with different methods on the reproducibility of quantitative and qualitative results from iCAMP. (a) Results from

simulated communities under the 12th situation (75% selection, 25% dispersal, Supplementary Table 1) in low-phylogenetic-signal scenario. **(b)** Results from simulated communities under the 13th situation (25% selection, 25% dispersal, 50% drift, Supplementary Table 1) in low-phylogenetic-signal scenario. Deviation from expectation is calculated as the difference of the estimated relative importance of a process from the result of the original data. Process difference rate, percentage of the estimated dominated processes which are different from the results of original data. In the box plots, top of box is 75th percentile; bottom of box is 25th percentile; center bar is median; whiskers show the maximum and minimum values within 1.5 of the interquartile range; dots represent outliers; triangle is mean value; n = 1,000 independent tests for the taxa number reduction. Source data are provided as a Source Data file.

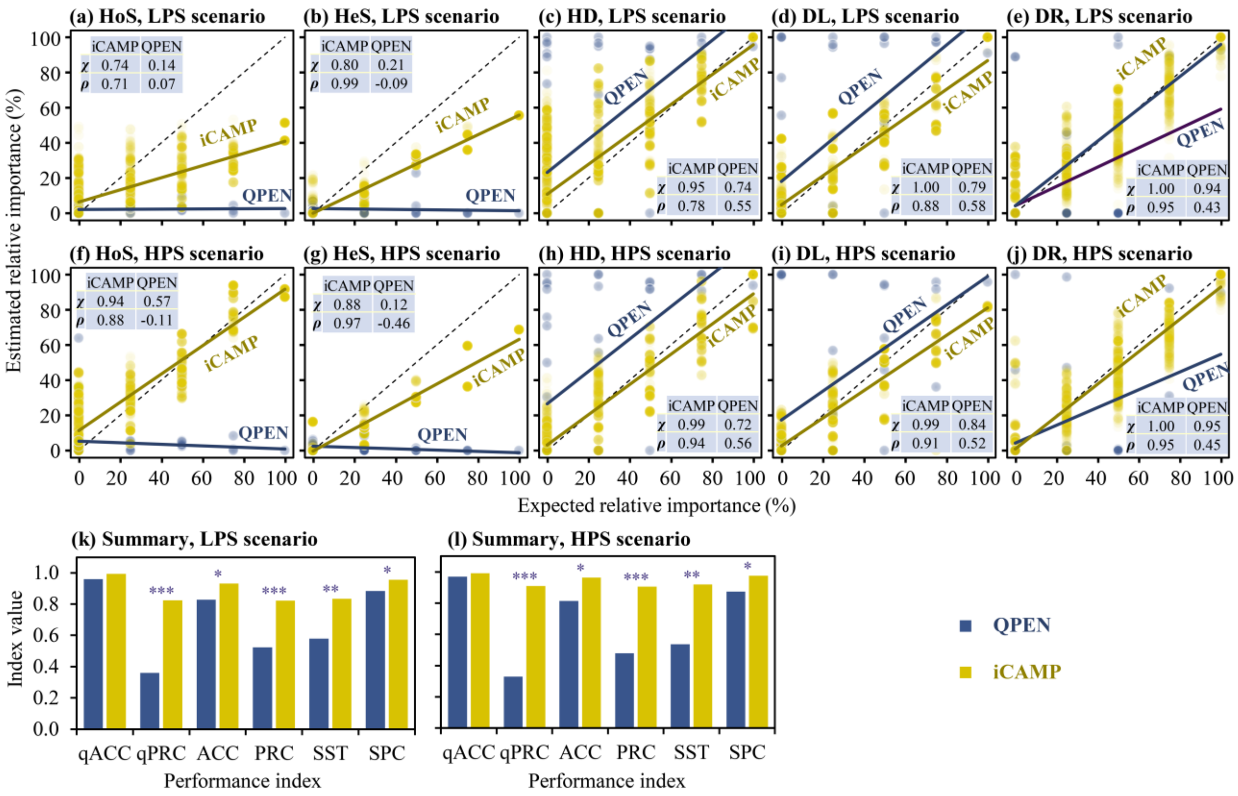

Supplementary Figure 9. Performances of iCAMP (dark yellow) and QPEN (livid) with simulated communities. The performance was evaluated by the consistency of estimated and expected relative importance of individual processes under different phylogenetic signal scenarios: **(a)** HoS under low-phylogenetic-signal scenario (LPS), **(b)** HeS under LPS, **(c)** HD under LPS, **(d)** DL under LPS, **(e)** DR under LPS, **(f)** HoS under high-phylogenetic-signal scenario (HPS), **(g)** HeS under HPS, **(h)** HD under HPS, **(i)** DL under HPS, and **(j)** DR under HPS. **(k)** Overall performance of iCAMP and QPEN under LPS evaluated by six performance indexes: quantitative accuracy (qACC, χ) and precision (qPRC, ρ), qualitative accuracy (ACC), precision (PRC), sensitivity (SST), and specificity (SPC); (**l**) Performance of iCAMP and qPEN under high-phylogenetic-signal scenario (HPS). For (**a-j**) and performance indexes calculation in (**k, l**), iCAMP n = 4140 comparisons = 276 comparisons among 24 biologically independent samples in each of the 15 situations; QPEN n = 60 groups = 4 groups of comparisons among 24 samples in each of the 15 situations. In (**k, l**), one-side difference significance test was based on n = 1000 times bootstrapping from 15 independent situations; ***, $p < 0.001$; **, $p < 0.01$; *, $p < 0.05$; p = 0.151, 0.000, 0.021, 0.000, 0.002, 0.029 under LPS for qACC, qPRC, ACC, PRC, SST, SPC, respectively; p = 0.311, 0.000, 0.012, 0.000, 0.002, 0.022 under HPS for qACC, qPRC, ACC, PRC, SST, SPC, respectively. Source data are provided as a Source Data file.

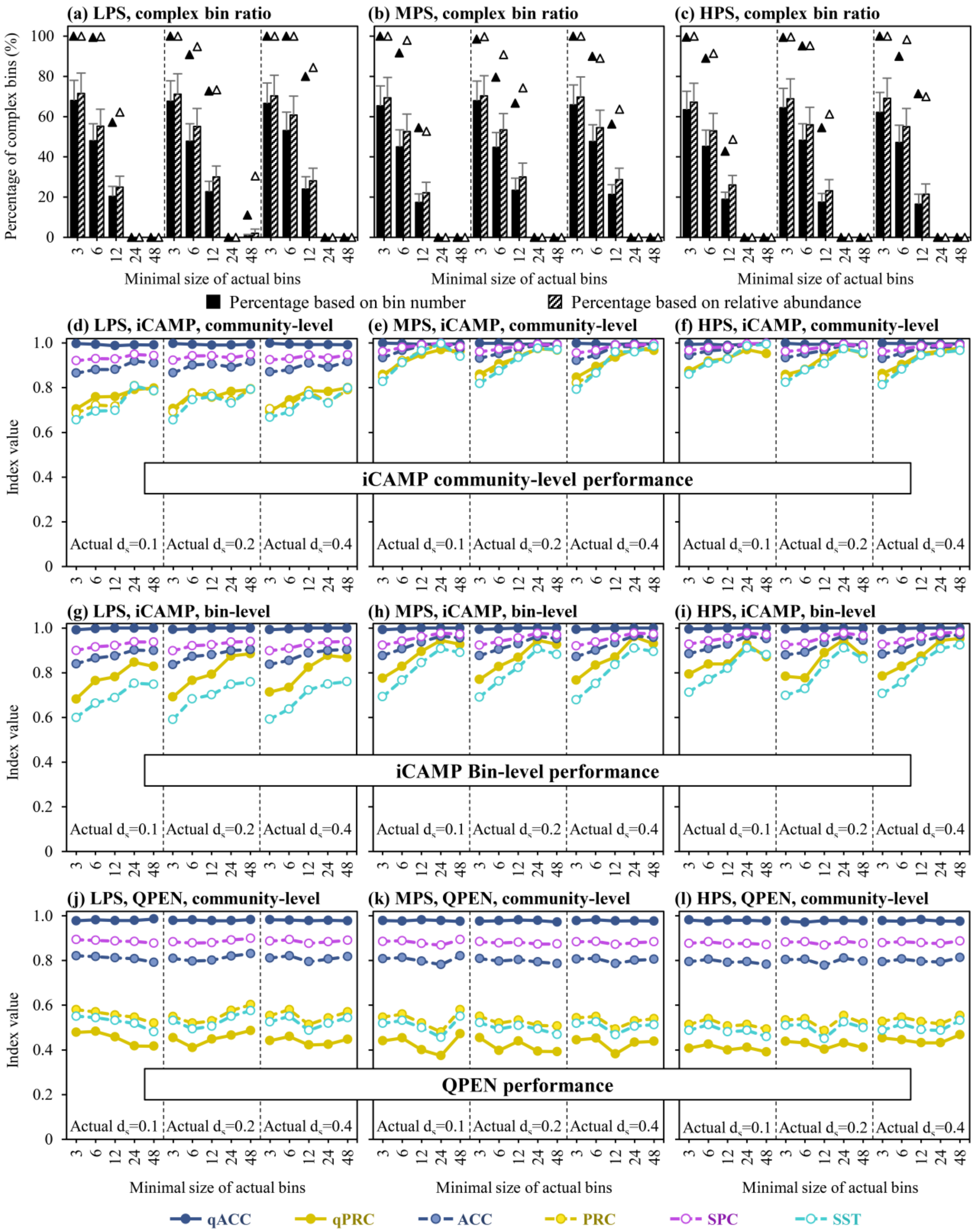

Supplementary Figure 10. Performance of iCAMP and QPEN with simulated communities when individual bins are governed by multiple processes. (a-c) Percentage of complex bins based on bin number (black filled) or relative abundance (diagonal) in different simulated datasets

under low- (LPS), medium- (MPS), and high-phylogenetic-signal (HPS) scenarios. The simulations are based on different actual bin size limitation and distance threshold (d_s). But iCAMP still estimates bins with a certain minimal bin size of 24 and threshold of 0.2, resulting in many estimated bins individually governed by multiple processes, so-called complex bins. Data are presented as mean values ± SD. Error bars indicate standard errors (n = 15 independent situations), and triangles indicates maximums in different simulated situations. **(d-f)** iCAMP performance at community level and **(g-i)** bin level, under different scenarios. **(j-l)** QPEN performance. Performance indexes: qACC and qPRC, quantitative accuracy and precision; ACC, PRC, SPC, and SST, qualitative accuracy, precision, specificity, and sensitivity, respectively. Source data are provided as a Source Data file.

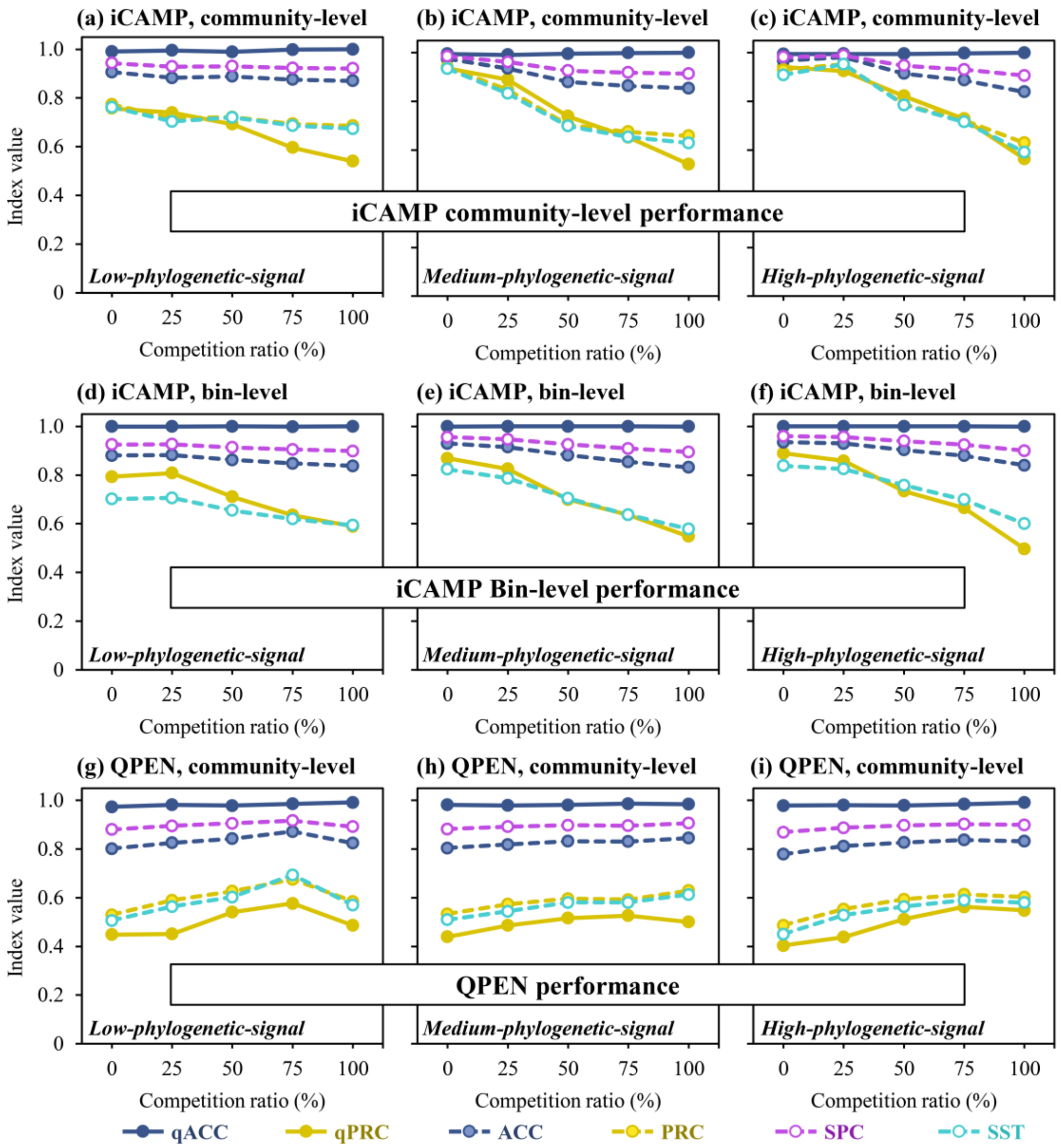

Supplementary Figure 11. Performance of iCAMP and QPEN with simulated communities when the roles of competition in selection vary from 0 to 100%. (a-c) iCAMP performance at community level and **(b-f)** bin level under different phylogenetic signal scenarios. **(g-i)** QPEN performance. Performance indexes: qACC and qPRC, quantitative accuracy and precision; ACC, PRC, SPC, and SST, qualitative accuracy, precision, specificity, and sensitivity, respectively. Source data are provided as a Source Data file.

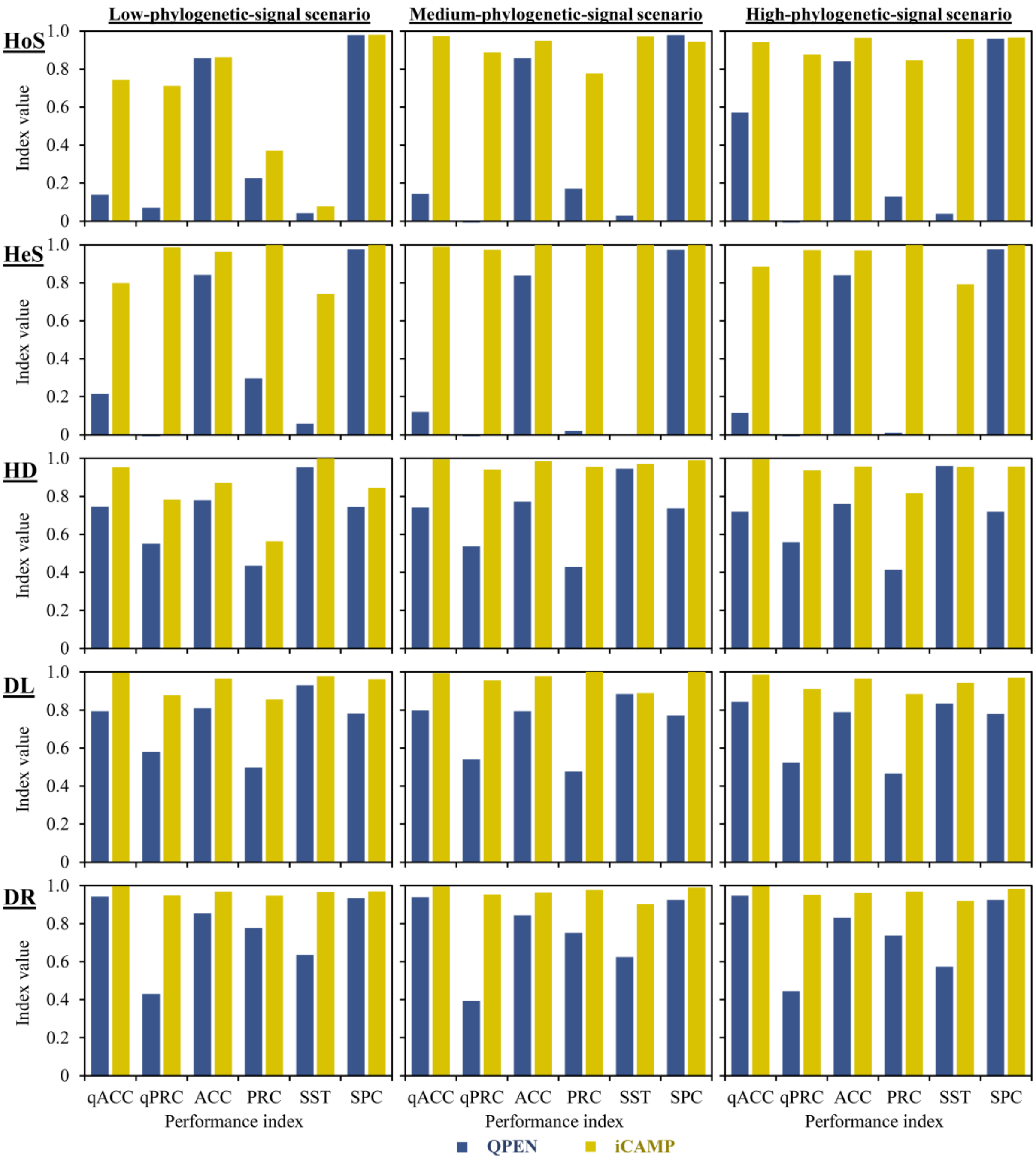

Supplementary Figure 12. Performances of iCAMP (dark yellow) and QPEN (livid) in assessing the relative importance of different ecological processes in simulated communities. iCAMP and QPEN were applied to simulated communities under different scenarios with low, medium, and high phylogenetic signals. Performance indexes were abbreviated as in Supplementary Figure 3. Source data are provided as a Source Data file.

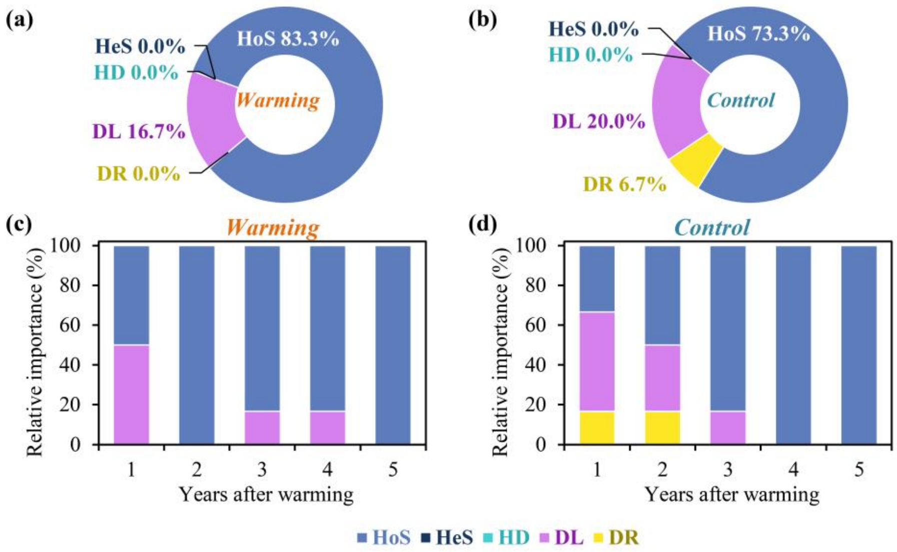

Supplementary Figure 13. Relative importance of different assembly processes estimated with QPEN. (a) Average relative importance of different processes in bacterial assembly under warming, and **(b)** under control. **(c)** Relative importance of different ecological processes under warming and **(d)** under control each year. Source data are provided as a Source Data file.

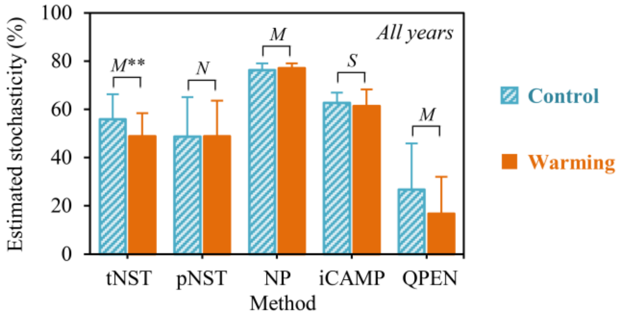

Supplementary Figure 14. Stochasticity estimated by different methods with the experimental data across all 5 years after warming. tNST and pNST represent taxonomic and phylogenetic normalized stochasticity ratio, respectively; NP represents abundance-weighted neutral taxa percentage. One-side significance based on bootstrapping was indicated as ***, $p < 0.01$; **, $p < 0.05$ (p = 0.015, 0.797, 0.270, 0.390, 0.367 for tNST, pNST, NP, iCAMP, QPEN, respectively); L, M, S, and N represent large, medium, small, and negligible effect sizes, according to Cohen's d estimated as the mean difference between warming and control divided by pooled standard deviation. Data are presented as mean values ± SD. Error bars represent standard deviations (n = 30 comparisons = 6 comparisons among 4 biologically independent samples in each of the 5 years). Source data are provided as a Source Data file.

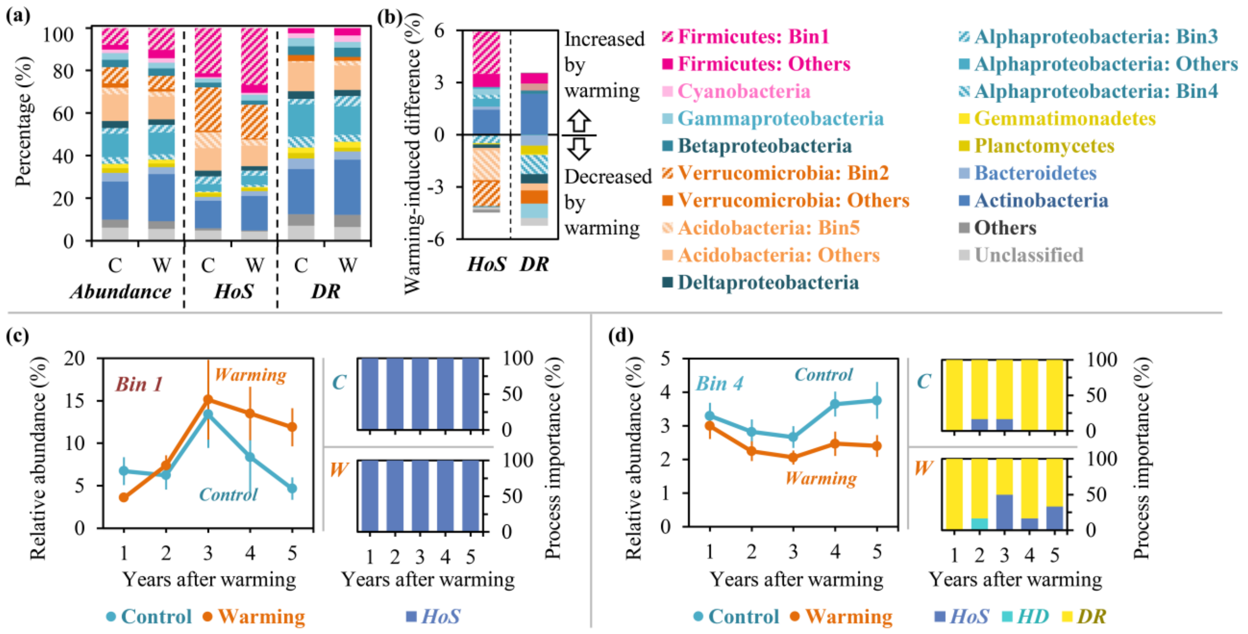

Supplementary Figure 15. Ecological processes controlling major phylogenetic bins. (a) Relative abundances of different phyla and their relative contributions to homogeneous selection (HoS) and drift (DR) under control (C) and warming (W). Top 5 abundant bins were particularly highlighted. **(b)** Warming-induced changes of different phyla in the later 3 years. **(c)** and **(d),** relative abundances of Bin 1 and Bin 4, and relative importance of ecological processes controlling their assembly under control (C, aqua) and warming (W, orange). Error bars indicate standard error (n = 4). Source data are provided as a Source Data file.

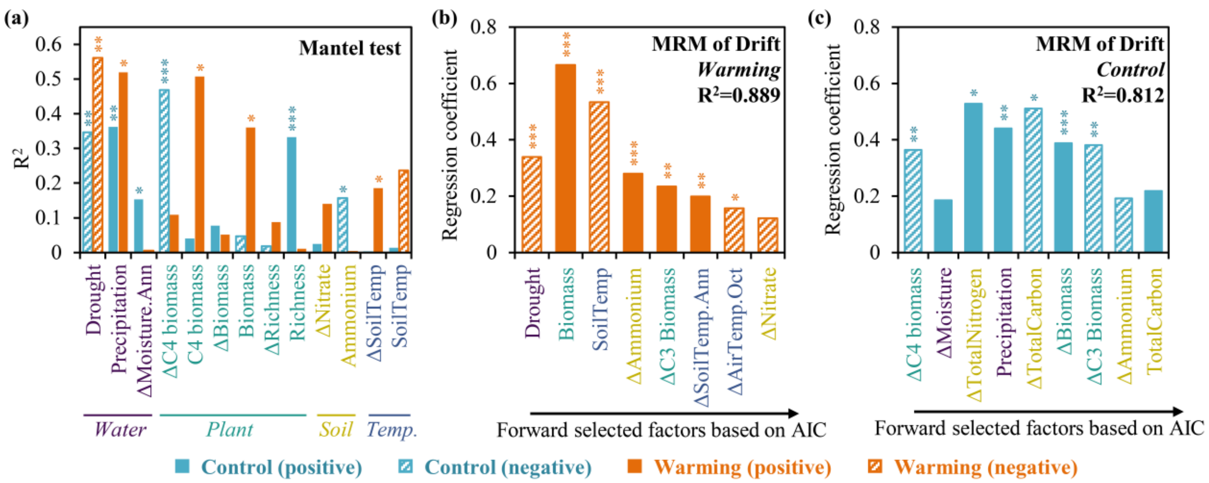

Supplementary Figure 16. Effects of environmental factors on drift under warming (orange bars) and control (aqua bars). (a) Correlations based on Mantel test. This figure only showed the factors with significant correlation in Mantel test, see Supplementary Table 2 for other factors. **(b)** Multiple Regression on distance Matrix (MRM) under warming; and **(c)** MRM under control. R^2, coefficient of determination based on the best model from Mantel analysis (see Supplementary Table 2 for details). The correlation was determined based on the difference (with a triangle before the name) or the mean (without triangle) of a factor between each pair of samples. Factors marked with '.Ann' are annual means, while other factors were measured in the sampling month. Significance was expressed as ***, $p < 0.01$; **, $p < 0.05$; *, $p < 0.1$ (see Source Data file for exact p values). Temp, temperature. Source data are provided as a Source Data file.

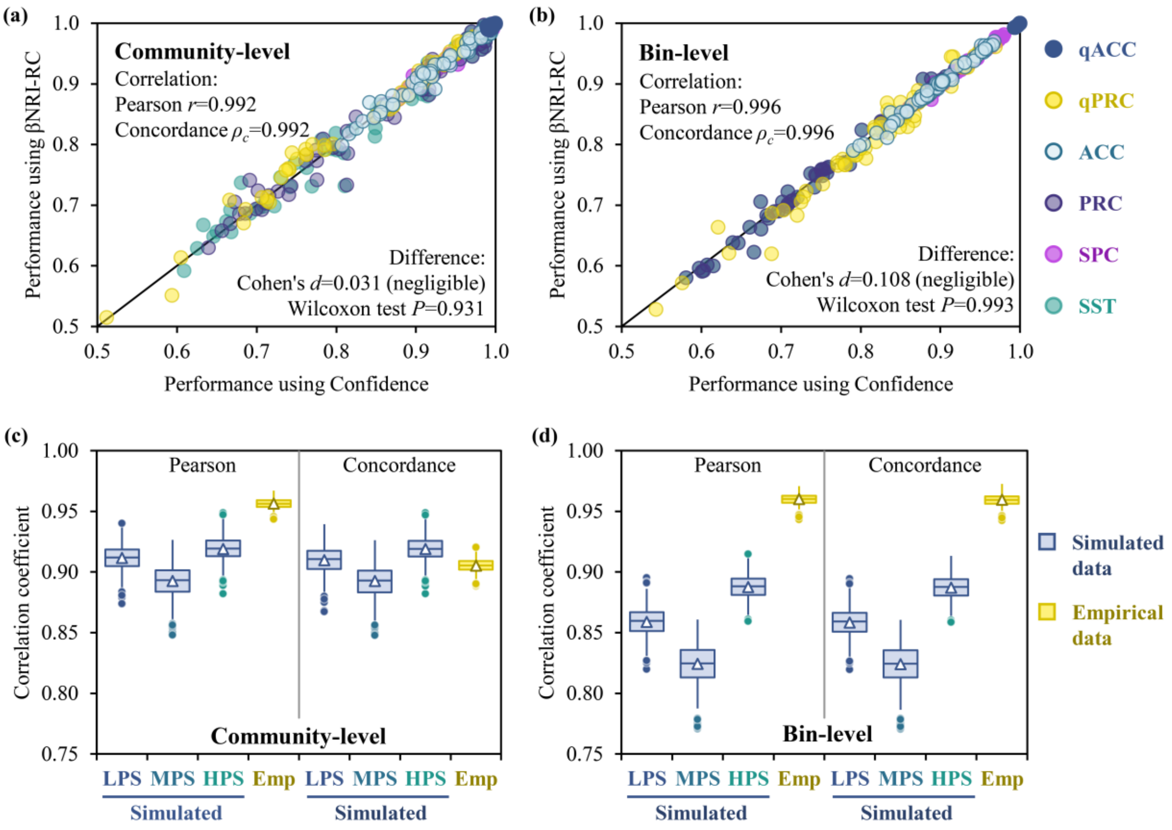

Supplementary Figure 17. Effect of null model significance testing indexes (βNRI-RC versus Confidence) on iCAMP performance and results. The 'Confidence' is calculated by direct counting the percentage of null values higher or lower than the observed value, i.e. non-parametric one-tail confidence level. **(a, b)** Correlation of iCAMP performance using different significance testing indexes, at community and bin level. **(c, d)** Correlation coefficients of process importance estimated by iCAMP using different significance testing indexes. At both community and bin levels, βNRI-RC and Confidence led to almost identical performance of iCAMP (correlation higher than 0.99, negligible difference with Cohen's $|d|<0.11$ and Wilcoxon $P>0.9$) and highly similar results in terms of process relative importance (correlation higher than 0.77, negligible difference with Cohen's $|d|<10^{-10}$ and Wilcoxon $P>0.36$). The comparison counts in 57 sets of simulated situations (each set has 15 situations, i.e. 855 situations), including those considering complex processes in each bin (Supplementary Figure 10 and 11), in addition to the empirical dataset. The similarity is demonstrated with both Pearson's correlation and Lin's concordance correlation coefficients. iCAMP performance indexes are abbreviated as Supplementary Figure 3. LPS, MPS, and HPS indicate low-, medium-, and high-phylogenetic-signal scenarios. Emp, Empirical data. (**a, b**) n = 342 = 6 indexes for each of the 57 data sets. (**c, d**) box and whisker, quartiles; triangle, mean value; n = 855 situations. Source data are provided as a Source Data file.

Supplementary Tables

Supplementary Table 1. Defined (expected) relative importance of different ecological processes under various simulated situations. Each simulated scenario has following 15 situations. The location of plot HA, HB, LA, and LB is showed in Fig. S2a. HeS, heterogeneous selection; HoS, homogeneous selection; DL, dispersal limitation; HD, homogenizing dispersal; DR, "drift", including drift, stochastic diversification, weak selection, and/or weak dispersal; ST, expected stochasticity.

Situation	Selection	Dispersal	Drift	Comparison	HeS	HoS	DL	HD	DR	ST
1	100%	0%	0%	HA vs HB	0%	100%	0%	0%	0%	0%
				HA vs LA	100%	0%	0%	0%	0%	
				HA vs LB	100%	0%	0%	0%	0%	
				HB vs LA	100%	0%	0%	0%	0%	
				HB vs LB	100%	0%	0%	0%	0%	
				LA vs LB	0%	100%	0%	0%	0%	
2	0%	100%	0%	HA vs HB	0%	0%	100%	0%	0%	100%
				HA vs LA	0%	0%	0%	100%	0%	
				HA vs LB	0%	0%	100%	0%	0%	
				HB vs LA	0%	0%	100%	0%	0%	
				HB vs LB	0%	0%	0%	100%	0%	
				LA vs LB	0%	0%	100%	0%	50%	
3	0%	0%	100%	HA vs HB	0%	0%	0%	0%	100%	100%
				HA vs LA	0%	0%	0%	0%	100%	
				HA vs LB	0%	0%	0%	0%	100%	
				HB vs LA	0%	0%	0%	0%	100%	
				HB vs LB	0%	0%	0%	0%	100%	
				LA vs LB	0%	0%	0%	0%	100%	
4	0%	25%	75%	HA vs HB	0%	0%	25%	0%	75%	100%
				HA vs LA	0%	0%	0%	25%	75%	
				HA vs LB	0%	0%	25%	0%	75%	
				HB vs LA	0%	0%	25%	0%	75%	
				HB vs LB	0%	0%	0%	25%	75%	
				LA vs LB	0%	0%	25%	0%	75%	
5	0%	50%	50%	HA vs HB	0%	0%	50%	0%	50%	100%
				HA vs LA	0%	0%	0%	50%	50%	
				HA vs LB	0%	0%	50%	0%	50%	
				HB vs LA	0%	0%	50%	0%	50%	
				HB vs LB	0%	0%	0%	50%	50%	
				LA vs LB	0%	0%	50%	0%	50%	
6	0%	75%	25%	HA vs HB	0%	0%	75%	0%	25%	100%
				HA vs LA	0%	0%	0%	75%	25%	
				HA vs LB	0%	0%	75%	0%	25%	
				HB vs LA	0%	0%	75%	0%	25%	
				HB vs LB	0%	0%	0%	75%	25%	
				LA vs LB	0%	0%	75%	0%	25%	
7	25%	0%	75%	HA vs HB	0%	25%	0%	0%	75%	75%
				HA vs LA	25%	0%	0%	0%	75%	
				HA vs LB	25%	0%	0%	0%	75%	
				HB vs LA	25%	0%	0%	0%	75%	
				HB vs LB	25%	0%	0%	0%	75%	
				LA vs LB	0%	25%	0%	0%	75%	

Supplementary Table 1. Continued

Situation	Selection	Dispersal	Drift	Comparison	HeS	HoS	DL	HD	DR	ST
8	50%	0%	50%	HA vs HB	0%	50%	0%	0%	50%	50%
				HA vs LA	50%	0%	0%	0%	50%	
				HA vs LB	50%	0%	0%	0%	50%	
				HB vs LA	50%	0%	0%	0%	50%	
				HB vs LB	50%	0%	0%	0%	50%	
				LA vs LB	0%	50%	0%	0%	50%	
9	75%	0%	25%	HA vs HB	0%	75%	0%	0%	25%	25%
				HA vs LA	75%	0%	0%	0%	25%	
				HA vs LB	75%	0%	0%	0%	25%	
				HB vs LA	75%	0%	0%	0%	25%	
				HB vs LB	75%	0%	0%	0%	25%	
				LA vs LB	0%	75%	0%	0%	25%	
10	25%	75%	0%	HA vs HB	0%	25%	75%	0%	0%	75%
				HA vs LA	25%	0%	0%	75%	0%	
				HA vs LB	25%	0%	75%	0%	0%	
				HB vs LA	25%	0%	75%	0%	0%	
				HB vs LB	25%	0%	0%	75%	0%	
				LA vs LB	0%	25%	75%	0%	0%	
11	50%	50%	0%	HA vs HB	0%	50%	50%	0%	0%	50%
				HA vs LA	50%	0%	0%	50%	0%	
				HA vs LB	50%	0%	50%	0%	0%	
				HB vs LA	50%	0%	50%	0%	0%	
				HB vs LB	50%	0%	0%	50%	0%	
				LA vs LB	0%	50%	50%	0%	0%	
12	75%	25%	0%	HA vs HB	0%	75%	25%	0%	0%	25%
				HA vs LA	75%	0%	0%	25%	0%	
				HA vs LB	75%	0%	25%	0%	0%	
				HB vs LA	75%	0%	25%	0%	0%	
				HB vs LB	75%	0%	0%	25%	0%	
				LA vs LB	0%	75%	25%	0%	0%	
13	25%	25%	50%	HA vs HB	0%	25%	25%	0%	50%	75%
				HA vs LA	25%	0%	0%	25%	50%	
				HA vs LB	25%	0%	25%	0%	50%	
				HB vs LA	25%	0%	25%	0%	50%	
				HB vs LB	25%	0%	0%	25%	50%	
				LA vs LB	0%	25%	25%	0%	50%	
14	25%	50%	25%	HA vs HB	0%	25%	50%	0%	25%	75%
				HA vs LA	25%	0%	0%	50%	25%	
				HA vs LB	25%	0%	50%	0%	25%	
				HB vs LA	25%	0%	50%	0%	25%	
				HB vs LB	25%	0%	0%	50%	25%	
				LA vs LB	0%	25%	50%	0%	25%	
15	50%	25%	25%	HA vs HB	0%	50%	25%	0%	25%	50%
				HA vs LA	50%	0%	0%	25%	25%	
				HA vs LB	50%	0%	25%	0%	25%	
				HB vs LA	50%	0%	25%	0%	25%	
				HB vs LB	50%	0%	0%	25%	25%	
				LA vs LB	0%	50%	25%	0%	25%	

Supplementary Table 2. Coefficient of determination (R^2) between the relative importance of HoS or DR and each factor. The relative importance of HoS and DR was estimated by iCAMP. The correlation was analyzed by modified Mantel test based on linear model (LM) and general linear model (GLM). The relative importance of an ecological process (Y) and each factor (X) were either log-transformed or not before fitting the models, to test linear (Y ~ X), logarithmic (Y ~ lnX), exponential (lnY ~ X), and power law (lnY ~ lnX) relationship. The best model was selected based on R^2. In the factor abbreviations, the first letter ("d" or "m") indicated whether the difference or the mean of the factor for each pair of samples were performed by correlation analysis; ABC3, aboveground biomass of C-3 plants; ABC4, aboveground biomass of C-4 plants; ABT, aboveground biomass of all plants; PRn, plant richness; pH, soil pH; TC, total soil carbon; TN, total soil nitrogen; NO3, soil nitrate ; NH4, soil ammonium nitrogen; SMoi, soil moisture in sampling month; AMoi, annual mean of soil moisture; SPr, precipitation in sampling month; APr, annual precipitation; SDI, drought index in the sampling month, which is additive inverse of the standardized precipitation-evapotranspiration index (SPEI) in the sampling month; ADI, annual mean of the drought index, which was additive inverse of annual mean SPEI; AST, annual mean of soil temperature; SST, mean soil temperature in sampling month; AAT, annual mean of air temperature; SAT, mean air temperature in sampling month; WY, warming years; X, eastward coordinate; Y, northward coordinate; Dist, geographic distance; PCNM1-5, principal coordinates of neighborhood matrix calculated from geographic distance. Significance is indicated as ***, $p < 0.01$; **, $p < 0.05$; *, $p < 0.1$.

X: factor	Control						Warming						Control	Warming
	Mantel-LM				Mantel-GLM		Mantel-LM				Mantel-GLM		Best model results	
	Y~X	Y~lnX	lnY~X	lnY~lnX	Y~X	Y~lnX	Y~X	Y~lnX	lnY~X	lnY~lnX	Y~X	Y~lnX		
(1) Y: HoS importance														
**dABC3**	0.002	0.013	0.000	0.007	0.002	0.013	0.007	0.004	0.008	0.009	0.007	0.004	0.013	0.009
**mABC3**	0.000	0.001	0.000	0.000	0.000	0.001	0.138	0.063	0.181	0.089	0.135	0.062	0.001	0.181
**dABC4**	0.241**	0.345***	0.218**	0.304***	0.244**	0.347***	0.065	0.049	0.048	0.033	0.067	0.051	0.347***	0.067
**mABC4**	0.003	0.046	0.005	0.038	0.003	0.045	0.328*	0.498*	0.292*	0.441*	0.338*	0.500*	0.046	0.500*
**dABT**	0.118*	0.103*	0.129*	0.120*	0.116*	0.102*	0.042	0.012	0.037	0.022	0.042	0.012	0.129*	0.042
**mABT**	0.000	0.006	0.000	0.006	0.000	0.005	0.370*	0.255	0.419*	0.297	0.364*	0.248	0.006	0.419*
**dPRn**	0.003	0.010	0.003	0.010	0.003	0.010	0.101*	0.128	0.102*	0.131	0.100*	0.126	0.010	0.131
**mPRn**	0.332***	0.278***	0.323***	0.271**	0.336***	0.277***	0.016	0.005	0.016	0.006	0.016	0.005	0.336***	0.016
**dpH**	0.001	0.001	0.001	0.001	0.001	0.001	0.087	0.083	0.078	0.074	0.087	0.083	0.001	0.087
**mpH**	0.022	0.022	0.027	0.027	0.022	0.022	0.109	0.103	0.129	0.123	0.107	0.101	0.027	0.129
**dTC**	0.004	0.009	0.002	0.007	0.004	0.009	0.000	0.000	0.002	0.004	0.000	0.000	0.009	0.004
**mTC**	0.007	0.009	0.007	0.007	0.007	0.009	0.028	0.031	0.032	0.032	0.028	0.031	0.009	0.032
**dTN**	0.012	0.022	0.009	0.018	0.012	0.022	0.001	0.000	0.000	0.002	0.001	0.000	0.022	0.002
**mTN**	0.013	0.018	0.012	0.016	0.013	0.018	0.011	0.013	0.012	0.012	0.011	0.013	0.018	0.013
**dNO3**	0.006	0.019	0.007	0.021	0.006	0.019	0.049	0.223*	0.046	0.207*	0.049	0.223*	0.021	0.223*
**mNO3**	0.011	0.067	0.012	0.069	0.011	0.067	0.001	0.046	0.001	0.056	0.001	0.044	0.069	0.056
**dNH4**	0.053	0.034	0.061	0.040	0.053	0.034	0.022	0.045	0.019	0.041	0.022	0.044	0.061	0.045
**mNH4**	0.134*	0.152*	0.142*	0.158*	0.133*	0.152*	0.000	0.002	0.000	0.002	0.000	0.002	0.158*	0.002

Supplementary Table 2. Continued

X: factor	Control						Warming						Control	Warming
	Mantel-LM				Mantel-GLM		Mantel-LM				Mantel-GLM		Best model results	
	Y~X	Y~lnX	lnY~X	lnY~lnX	Y~X	Y~lnX	Y~X	Y~lnX	lnY~X	lnY~lnX	Y~X	Y~lnX		
**(1) Y: HoS importance**														
**dSMoi**	0.002	0.002	0.001	0.005	0.002	0.002	0.102	0.011	0.131	0.021	0.099	0.011	0.005	0.131
**mSMoi**	0.003	0.005	0.002	0.004	0.003	0.005	0.046	0.014	0.073	0.027	0.045	0.014	0.005	0.073
**dAMoi**	0.228**	0.273***	0.264***	0.310***	0.226**	0.271***	0.006	0.009	0.006	0.013	0.006	0.009	0.310***	0.013
**mAMoi**	0.011	0.011	0.007	0.008	0.011	0.011	0.080	0.058	0.109	0.081	0.078	0.056	0.011	0.109
**mSPr**	0.253***	0.305***	0.234***	0.281***	0.256***	0.305***	0.411*	0.526*	0.367	0.464*	0.423*	0.527*	0.305***	0.527*
**mAPr**	0.009	0.004	0.013	0.007	0.009	0.004	0.083	0.068	0.084	0.071	0.082	0.067	0.013	0.084
**mSDI**	0.149	0.300**	0.138	0.278**	0.151	0.301**	0.254	0.567*	0.235	0.511*	0.264	0.569*	0.301**	0.569*
**mADI**	0.008	0.013	0.011	0.010	0.008	0.013	0.028	0.003	0.023	0.003	0.027	0.003	0.013	0.028
**dAST**	0.031	0.029	0.027	0.025	0.031	0.029	0.001	0.001	0.001	0.000	0.001	0.001	0.031	0.001
**mAST**	0.008	0.010	0.007	0.008	0.008	0.009	0.217	0.224	0.204	0.209	0.216	0.222	0.010	0.224
**dSST**	0.006	0.004	0.007	0.005	0.006	0.004	0.077	0.135	0.085	0.147*	0.078	0.136	0.007	0.147*
**mSST**	0.005	0.004	0.007	0.006	0.005	0.004	0.313*	0.321*	0.314*	0.324*	0.313*	0.322*	0.007	0.324*
**dAAT**	0.037	0.041	0.034	0.039	0.037	0.042	0.067	0.042	0.074	0.050	0.067	0.041	0.042	0.074
**mAAT**	0.043	0.041	0.041	0.039	0.043	0.042	0.056	0.047	0.036	0.029	0.057	0.049	0.043	0.057
**dSAT**	0.050	0.045	0.059	0.053	0.050	0.045	0.021	0.019	0.031	0.029	0.020	0.019	0.059	0.031
**mSAT**	0.040	0.035	0.048	0.043	0.039	0.035	0.043	0.043	0.038	0.038	0.044	0.043	0.048	0.044
**mWY**							0.262	0.376	0.312	0.434	0.254	0.368		0.434
**dX**	0.008	0.006	0.011	0.010	0.008	0.006	0.000	0.000	0.000	0.000	0.000	0.000	0.011	0.000
**mX**	0.010	0.000	0.008	0.000	0.010	0.000	0.000	0.000	0.000	0.000	0.000	0.000	0.010	0.000
**dY**	0.002	0.002	0.005	0.005	0.002	0.002	0.006	0.000	0.006	0.000	0.006	0.000	0.005	0.006
**mY**	0.000	0.000	0.000	0.000	0.000	0.000	0.008*	0.007**	0.010**	0.011***	0.008*	0.008**	0.000	0.011***
**Dist**	0.001	0.000	0.000	0.000	0.001	0.000	0.005	0.005	0.005	0.004	0.005	0.005	0.001	0.005
**dPCNM1**	0.000	0.005	0.003	0.007	0.000	0.005	0.007	0.011	0.008	0.014	0.007	0.011	0.007	0.014
**mPCNM1**	0.003	0.005	0.003	0.006	0.003	0.005	0.008**	0.008*	0.010***	0.009**	0.008**	0.008*	0.006	0.010***
**dPCNM2**	0.001	0.035	0.001	0.034	0.001	0.035	0.000	0.001	0.000	0.001	0.000	0.001	0.035	0.001
**mPCNM2**	0.016	0.036*	0.014	0.035*	0.016	0.036*	0.000	0.001	0.000	0.001	0.000	0.001	0.036*	0.001
**dPCNM3**	0.001	0.006	0.002	0.009	0.001	0.006	0.002	0.001	0.002	0.001	0.002	0.001	0.009	0.002
**mPCNM3**	0.045*	0.007	0.051*	0.010	0.044*	0.007	0.000	0.000	0.001	0.000	0.000	0.000	0.051*	0.001
**dPCNM4**	0.012	0.008	0.012	0.010	0.012	0.008	0.002	0.000	0.003	0.000	0.002	0.000	0.012	0.003
**mPCNM4**	0.000	0.002	0.000	0.003	0.000	0.002	0.002	0.000	0.002	0.000	0.002	0.000	0.003	0.002
**dPCNM5**	0.016	0.011	0.021	0.015	0.016	0.011	0.004	0.008*	0.003	0.010*	0.004	0.008*	0.021	0.010*
**mPCNM5**	0.001	0.004	0.003	0.006	0.001	0.004	0.001	0.007*	0.001	0.009	0.001	0.007*	0.006	0.009

Supplementary Table 2. Continued

X: factor	Control						Warming						Control	Warming
	Mantel-LM				Mantel-GLM		Mantel-LM				Mantel-GLM		Best model results	
	Y~X	Y~lnX	lnY~X	lnY~lnX	Y~X	Y~lnX	Y~X	Y~lnX	lnY~X	lnY~lnX	Y~X	Y~lnX		
**(2) Y: DR importance**														
**dABC3**	0.088	0.036	0.093	0.037	0.087	0.036	0.008	0.008	0.007	0.005	0.008	0.008	0.093	0.008
**mABC3**	0.016	0.020	0.014	0.020	0.016	0.020	0.130	0.067	0.108	0.054	0.128	0.067	0.020	0.130
**dABC4**	0.350***	0.442***	0.364***	0.469***	0.354***	0.443***	0.094	0.058	0.109	0.072	0.096	0.059	0.469***	0.109
**mABC4**	0.021	0.039	0.019	0.041	0.021	0.039	0.339*	0.481*	0.356*	0.507*	0.347*	0.482*	0.041	0.507*
**dABT**	0.024	0.078	0.026	0.074	0.024	0.077	0.048	0.018	0.051	0.012	0.049	0.018	0.078	0.051
**mABT**	0.025	0.046	0.022	0.044	0.024	0.046	0.361*	0.278	0.332	0.253	0.358*	0.274	0.046	0.361*
**dPRn**	0.001	0.018	0.001	0.017	0.001	0.018	0.077	0.087	0.076	0.087	0.077	0.087	0.018	0.087
**mPRn**	0.329***	0.290**	0.323***	0.284**	0.332***	0.290**	0.011	0.008	0.010	0.007	0.011	0.007	0.332***	0.011
**dpH**	0.000	0.001	0.000	0.000	0.000	0.001	0.103	0.099	0.103*	0.099	0.103	0.099	0.001	0.103
**mpH**	0.000	0.000	0.000	0.000	0.000	0.000	0.098	0.093	0.086	0.081	0.097	0.092	0.000	0.098
**dTC**	0.036	0.048	0.040	0.053	0.037	0.048	0.001	0.001	0.002	0.002	0.001	0.001	0.053	0.002
**mTC**	0.000	0.003	0.000	0.003	0.000	0.003	0.037	0.046	0.031	0.041	0.037	0.046	0.003	0.046
**dTN**	0.068	0.087	0.069	0.089*	0.068	0.087	0.003	0.001	0.004	0.002	0.003	0.001	0.089*	0.004
**mTN**	0.001	0.003	0.001	0.003	0.001	0.003	0.014	0.019	0.012	0.017	0.015	0.020	0.003	0.020
**dNO3**	0.025	0.009	0.019	0.008	0.025	0.009	0.039	0.137*	0.038	0.140*	0.038	0.137*	0.025	0.140*
**mNO3**	0.001	0.010	0.000	0.013	0.001	0.010	0.003	0.024	0.003	0.022	0.003	0.024	0.013	0.024
**dNH4**	0.032	0.014	0.027	0.011	0.032	0.014	0.042	0.093	0.041	0.088	0.042	0.093	0.032	0.093
**mNH4**	0.123	0.157*	0.111	0.145*	0.122	0.156*	0.000	0.002	0.000	0.002	0.000	0.002	0.157*	0.002
**dSMoi**	0.008	0.001	0.008	0.000	0.008	0.001	0.068	0.004	0.053	0.001	0.067	0.004	0.008	0.068
**mSMoi**	0.000	0.001	0.000	0.001	0.000	0.001	0.049	0.010	0.035	0.005	0.049	0.010	0.001	0.049
**dAMoi**	0.116*	0.153*	0.107*	0.142*	0.115*	0.152*	0.002	0.005	0.001	0.003	0.002	0.005	0.153*	0.005
**mAMoi**	0.039	0.039	0.039	0.040	0.039	0.039	0.077	0.055	0.062	0.044	0.076	0.055	0.040	0.077
**mSPr**	0.333**	0.355**	0.332**	0.362**	0.335**	0.354**	0.419*	0.493*	0.435*	0.520*	0.426*	0.492*	0.362**	0.520*
**mAPr**	0.003	0.008	0.002	0.007	0.002	0.008	0.049	0.037	0.050	0.037	0.048	0.036	0.008	0.050
**mSDI**	0.221	0.341**	0.216	0.347**	0.223	0.342**	0.284	0.539*	0.288	0.561**	0.291	0.539*	0.347**	0.561**
**mADI**	0.001	0.062	0.001	0.056	0.001	0.061	0.009	0.016	0.012	0.015	0.009	0.016	0.062	0.016
**dAST**	0.056	0.051	0.056	0.051	0.056	0.051	0.001	0.004	0.001	0.004	0.001	0.004	0.056	0.004
**mAST**	0.000	0.000	0.000	0.000	0.000	0.000	0.134	0.137	0.140	0.144	0.134	0.137	0.000	0.144
**dSST**	0.001	0.000	0.001	0.000	0.001	0.000	0.123	0.184*	0.115	0.174*	0.124	0.186*	0.001	0.186*
**mSST**	0.014	0.011	0.011	0.009	0.014	0.011	0.227	0.237	0.224	0.233	0.227	0.237	0.014	0.237
**dAAT**	0.074	0.079	0.074	0.079	0.074	0.079	0.132*	0.100	0.128*	0.093	0.132*	0.099	0.079	0.132*
**mAAT**	0.029	0.029	0.033	0.033	0.029	0.029	0.030	0.023	0.041	0.033	0.031	0.024	0.033	0.041
**dSAT**	0.023	0.020	0.021	0.018	0.023	0.020	0.000	0.000	0.000	0.000	0.000	0.000	0.023	0.000

Supplementary Table 2. Continued

X: factor	Control						Warming						Control	Warming
	Mantel-LM				Mantel-GLM		Mantel-LM				Mantel-GLM		Best model results	
	Y~X	Y~lnX	lnY~X	lnY~lnX	Y~X	Y~lnX	Y~X	Y~lnX	lnY~X	lnY~lnX	Y~X	Y~lnX		
**(2) Y: DR importance**														
**mSAT**	0.025	0.021	0.021	0.018	0.025	0.021	0.047	0.047	0.051	0.051	0.047	0.047	0.025	0.051
**mWY**							0.256	0.350	0.225	0.315	0.251	0.346		0.350
**dX**	0.013	0.002	0.011	0.001	0.013	0.002	0.000	0.000	0.000	0.000	0.000	0.000	0.013	0.000
**mX**	0.031	0.010	0.036	0.013	0.031	0.010	0.001	0.001	0.000	0.000	0.001	0.001	0.036	0.001
**dY**	0.000	0.000	0.000	0.000	0.000	0.000	0.005	0.008	0.005	0.009	0.005	0.008	0.000	0.009
**mY**	0.001	0.001	0.001	0.001	0.001	0.001	0.002	0.002	0.003	0.004	0.002	0.002	0.001	0.004
**Dist**	0.004	0.002	0.006	0.004	0.004	0.002	0.002	0.002	0.002	0.003	0.002	0.002	0.006	0.003
**dPCNM1**	0.004	0.000	0.008	0.001	0.004	0.000	0.011	0.008	0.010	0.006	0.011	0.008	0.008	0.011
**mPCNM1**	0.000	0.000	0.000	0.001	0.000	0.000	0.001	0.001	0.002	0.001	0.001	0.001	0.001	0.002
**dPCNM2**	0.000	0.043	0.000	0.046	0.000	0.043	0.000	0.002	0.000	0.002	0.000	0.002	0.046	0.002
**mPCNM2**	0.046*	0.045	0.050**	0.048	0.046*	0.045	0.000	0.002	0.000	0.002	0.000	0.002	0.050**	0.002
**dPCNM3**	0.009	0.000	0.011	0.000	0.009	0.000	0.013	0.008	0.015**	0.010	0.013	0.008	0.011	0.015**
**mPCNM3**	0.037	0.000	0.036	0.000	0.037	0.000	0.008*	0.008*	0.009	0.008	0.008*	0.008*	0.037	0.009
**dPCNM4**	0.000	0.000	0.000	0.000	0.000	0.000	0.004	0.000	0.003	0.000	0.004	0.000	0.000	0.004
**mPCNM4**	0.011	0.004	0.013	0.005	0.011	0.004	0.004	0.000	0.003	0.000	0.004	0.000	0.013	0.004
**dPCNM5**	0.004	0.002	0.003	0.001	0.004	0.002	0.001	0.001	0.001	0.001	0.001	0.001	0.004	0.001
**mPCNM5**	0.001	0.000	0.002	0.000	0.001	0.000	0.003	0.001	0.002	0.001	0.003	0.001	0.002	0.003

Supplementary Table 3. Correlation between environmental factors and major community assembly processes based on Mantel and partial Mantel test. Y, response variable, the relative importance of an assembly process, i.e. homogeneous selection (HoS) or drift (DR). X, an environmental factor. Z, the controlling variable in partial Mantel test. Environmental factors are log-transformed. Abbreviations are the same as Table S2. Only factors significantly correlated with the processes were showed. Interestingly, plant biomass (mABT) under warming and C4 plant biomass variation (dABC4) under control (bold) had very small change in correlation with the two processes when controlling other factors.

Treatment	Method	Y	Z	X							
Warming	Mantel	HoS	/	mSDI	mSPr	mABC4	**mABT**	dPRn	dNO3	dSST	mSST
				0.753**	-0.725**	-0.706*	**-0.505***	-0.357*	-0.472**	-0.367*	0.567*
	Partial Mantel		mSDI	/	0.141	-0.027	**-0.503***	-0.264	-0.129	-0.287	0.511*
			mSPr	0.323	/	-0.047	**-0.623***	-0.289*	-0.134	-0.361*	0.414
			mABC4	0.372	-0.241	/	**-0.618***	-0.407*	-0.192*	-0.407*	0.378
			mABT	0.752*	-0.782*	-0.764*	**/**	-0.292*	-0.536**	-0.192	0.723**
			dPRn	0.734*	-0.709*	-0.721*	**-0.468***	/	-0.452**	-0.318*	0.516*
			dNO3	0.672*	-0.633*	-0.614*	**-0.563***	-0.326*	/	-0.293	0.535*
			dSST	0.735*	-0.724*	-0.718*	**-0.413**	-0.306*	-0.423*	/	0.634*
			mSST	0.727**	-0.649*	-0.605*	**-0.690***	-0.239	-0.427**	-0.487**	/
Control	Mantel	HoS	/	mSDI	mSPr	dAMoi	**dABC4**	dABT	mPRn	mNH4	
				0.547***	-0.553***	-0.523***	**0.587*****	-0.321**	-0.527***	0.390*	
	Partial Mantel		mSDI	/	-0.093	-0.439***	**0.429****	-0.377**	-0.353*	0.036	
			mSPr	0.023	/	-0.439***	**0.423****	-0.320*	-0.373*	0.084	
			dAMoi	0.472*	-0.478	/	**0.603*****	-0.198	-0.492***	0.280*	
			dABC4	0.358	-0.359	-0.543***	**/**	-0.393**	-0.372**	0.290*	
			dABT	0.575***	-0.552**	-0.471***	**0.618*****	/	-0.534***	0.442**	
			mPRn	0.388	-0.414	-0.487***	**0.467****	-0.335**	/	0.120	
			mNH4	0.418*	-0.432*	-0.458***	**0.541*****	-0.386**	-0.401**	/	
Warming	Mantel	Drift	/	mSDI	mSPr	mABC4	**mABT**	dSST			
				-0.734**	0.702*	0.694*	**0.528***	0.429**			
	Partial Mantel		mSDI	/	-0.176	0.049	**0.528***	0.379*			
			mSPr	-0.345	/	0.099	**0.638***	0.443**			
			mABC4	-0.336	0.178	/	**0.641***	0.489**			
			mABT	-0.734*	0.764**	0.759**	**/**	0.263*			
			dSST	-0.718**	0.707*	0.718**	**0.420***	/			
Control	Mantel	Drift	/	mSDI	mSPr	dAMoi	**dABC4**	mPRn	mNH4		
				-0.584**	0.596**	0.391**	**-0.665*****	0.539***	-0.396*		
	Partial Mantel		mSDI	/	0.143	0.267*	**-0.526*****	0.354*	-0.005		
			mSPr	0.016	/	0.264*	**-0.518*****	0.376*	-0.055		
			dAMoi	-0.527*	0.540*	/	**-0.666*****	0.502**	-0.313*		
			dABC4	-0.384*	0.393	0.394**	**/**	0.369*	-0.291		
			mPRn	-0.434	0.468	0.327*	**-0.566****	/	-0.120		
			mNH4	-0.468*	0.487*	0.306*	**-0.628*****	0.413*	/		

Supplementary References

1. Wang, J. J. et al. Phylogenetic beta diversity in bacterial assemblages across ecosystems: deterministic versus stochastic processes. *ISME J.* **7**, 1310-1321 (2013).
2. Tripathi, B. M. et al. Soil pH mediates the balance between stochastic and deterministic assembly of bacteria. *ISME J.* **12**, 1072-1083 (2018).
3. Stegen, J. C. et al. Quantifying community assembly processes and identifying features that impose them. *ISME J.* **7**, 2069 (2013).
4. Goberna, M. & Verdú, M. Predicting microbial traits with phylogenies. *ISME J.* **10**, 959 (2015).
5. Cohen, J. *Statistical power analysis for the behavioral sciences*, 2nd edn. Academic Press (1988).
6. Chen, C. et al. Oral microbiota of periodontal health and disease and their changes after nonsurgical periodontal therapy. *ISME J.* **12**, 1210 (2018).
7. Zhou, J. & Ning, D. Stochastic community assembly: Does it matter in microbial ecology? *Microbiol. Mol. Biol. Rev.* **81**, (2017).
8. Stegen, J. C. et al. Groundwater-surface water mixing shifts ecological assembly processes and stimulates organic carbon turnover. *Nat. Commun.* **7**, 11237 (2016).
9. Dini-Andreote, F., Stegen, J. C., van Elsas, J. D. & Salles, J. F. Disentangling mechanisms that mediate the balance between stochastic and deterministic processes in microbial succession. *Proc. Natl. Acad. Sci. U.S.A.* **112**, E1326-E1332 (2015).
10. Stegen, J. C., Lin, X., Konopka, A. E. & Fredrickson, J. K. Stochastic and deterministic assembly processes in subsurface microbial communities. *ISME J.* **6**, 1653–1664 (2012).
11. Stegen, J. C., Lin, X., Fredrickson, J. K. & Konopka, A. E. Estimating and mapping ecological processes influencing microbial community assembly. *Front. Microbiol.* **6**, 370 (2015).
12. Wen, C. et al. Evaluation of the reproducibility of amplicon sequencing with Illumina MiSeq platform. *PLOS ONE* **12**, e0176716 (2017).
13. Zhou, J. et al. Random sampling process leads to overestimation of β-diversity of microbial communities. *mBio* **4**, e00324-00313 (2013).
14. Zhou, J. et al. Reproducibility and quantitation of amplicon sequencing-based detection. *ISME J.* **5**, 1303-1313 (2011).
15. Kane, M. J., Emerson, J. W. & Weston, S. Scalable Strategies for Computing with Massive Data. *J. Stat. Softw.* **55**, 1-19 (2013).
16. Veech, J. A. Significance testing in ecological null models. *Theor. Ecol.* **5**, 611-616 (2012).
17. Ning, D., Deng, Y., Tiedje, J. M. & Zhou, J. A general framework for quantitatively assessing ecological stochasticity. *Proc. Natl. Acad. Sci. U.S.A.* **116**, 16892-16898 (2019).
