# Supplementary material for: A quantitative framework reveals ecological drivers of grassland microbial community assembly in response to warming
Source: Nat Commun. 2020 Sep 18;11:4717. doi: 10.1038/s41467-020-18560-z (PMC7501310; doi:10.1038/s41467-020-18560-z)
Supplement: Supplementary file 3 — Reporting Summary [file 41467_2020_18560_MOESM3_ESM.pdf]

nature research

Corresponding author(s): Jizhong Zhou

Last updated by author(s): Aug 23, 2020

Reporting Summary

Nature Research wishes to improve the reproducibility of the work that we publish. This form provides structure for consistency and transparency in reporting. For further information on Nature Research policies, see our Editorial Policies and the Editorial Policy Checklist.

Statistics

For all statistical analyses, confirm that the following items are present in the figure legend, table legend, main text, or Methods section.

n/a	Confirmed	
☐	☒	The exact sample size (*n*) for each experimental group/condition, given as a discrete number and unit of measurement
☐	☒	A statement on whether measurements were taken from distinct samples or whether the same sample was measured repeatedly
☐	☒	The statistical test(s) used AND whether they are one- or two-sided<br>*Only common tests should be described solely by name; describe more complex techniques in the Methods section.*
☐	☒	A description of all covariates tested
☐	☒	A description of any assumptions or corrections, such as tests of normality and adjustment for multiple comparisons
☐	☒	A full description of the statistical parameters including central tendency (e.g. means) or other basic estimates (e.g. regression coefficient) AND variation (e.g. standard deviation) or associated estimates of uncertainty (e.g. confidence intervals)
☐	☒	For null hypothesis testing, the test statistic (e.g. *F*, *t*, *r*) with confidence intervals, effect sizes, degrees of freedom and *P* value noted<br>*Give P values as exact values whenever suitable.*
☒	☐	For Bayesian analysis, information on the choice of priors and Markov chain Monte Carlo settings
☒	☐	For hierarchical and complex designs, identification of the appropriate level for tests and full reporting of outcomes
☐	☒	Estimates of effect sizes (e.g. Cohen's *d*, Pearson's *r*), indicating how they were calculated

Our web collection on statistics for biologists contains articles on many of the points above.

Software and code

Policy information about availability of computer code

Data collection	Simulated community data were generated with custom scripts (available from GitHub https://github.com/DaliangNing/iCAMP1) written by R (version 3.5.3). Empirical data of 16S rRNA gene sequencing were processed by our pipeline (http://zhoulab5.rccc.ou.edu:8080) built on Galaxy platform (Version 17.01) with UPARSE from USEARCH (Version 9.2).
Data analysis	All statistical analyses were implemented by R (version 3.5.3), detailed in the method section. R package 'sads' (version 0.4.2) and function 'fastBM' in R package 'phytools' (version 0.6-99) were used to simulate metacommunity and trait evolution, respectively. The R package 'iCAMP' is available on CRAN (The Comprehensive R Archive Network, https://cran.r-project.org/). All custom scripts in this manuscript are available in GitHub (https://github.com/DaliangNing/iCAMP1).

For manuscripts utilizing custom algorithms or software that are central to the research but not yet described in published literature, software must be made available to editors and reviewers. We strongly encourage code deposition in a community repository (e.g. GitHub). See the Nature Research guidelines for submitting code & software for further information.

Data

Policy information about availability of data

All manuscripts must include a data availability statement. This statement should provide the following information, where applicable:

- Accession codes, unique identifiers, or web links for publicly available datasets
- A list of figures that have associated raw data
- A description of any restrictions on data availability

The sequencing data are available in the NCBI Sequence Read Archive under project no. PRJNA331185. The source data underlying Figs 2 to 5 and Supplementary Figs 3 to 17 are provided in the Source Data file. Other source data are all available from GitHub (https://github.com/DaliangNing/iCAMP1), such as OTU tables, phylogenetic trees, treatment information, etc. Other data are available from the authors upon reasonable request.

Field-specific reporting

Please select the one below that is the best fit for your research. If you are not sure, read the appropriate sections before making your selection.

☐ Life sciences ☐ Behavioural & social sciences ☒ Ecological, evolutionary & environmental sciences

For a reference copy of the document with all sections, see nature.com/documents/nr-reporting-summary-flat.pdf

Ecological, evolutionary & environmental sciences study design

All studies must disclose on these points even when the disclosure is negative.

Study description	This study is to develop a framework for quantitative understanding of microbial community assembly mechanisms. The empirical dataset is from an experimental warming grassland filed. The field site experiment was established in 2009. Surface soil temperature in warming plots is increased to 2 to 3°C higher than the controls by utilizing infrared radiator. Surface soil samples were taken annually from 4 warming and 4 control plots.
Research sample	Because this study focused on the effect of experimental warming on soil microbial assembly mechanisms, we selected all the four warming plots and four control plots without any other experimental treatment. In addition, our previous study (Guo et al. 2018. Nature Climate Change 8: 813-818) has found obvious effect of warming on the soil microbial communities in 5-year warming and the yearly samples well reflected the impact, thus, a total of 40 yearly samples over 5 years were analyzed in this study. The samples are grassland soil samples and analyzed for bacterial community structure by 16S rRNA gene sequencing. Data are collected from the previous published paper (Guo et al. 2018. Nature Climate Change 8: 813-818).
Sampling strategy	Surface soil samples were taken annually from 4 warming and 4 control plots. Each sample was mixed from three soil cores (2.5 cm diameter × 15 cm depth) by using a soil sampler tube. A total of 40 samples over 5 years after the warming infrastructure construction finished were analyzed in this study. Because on the experiment site, we only have 4 warming plots and 4 control plots that are not combined with other treatments, which fit our need to focus on warming effect. Moreover, our previous study (Guo et al. 2018. Nature Climate Change 8: 813-818) has found significant effect of warming on soil microbial community structure from these samples, demonstrating sufficient statistical power of the sample size to capture microbial community responses. Besides, the sample size in each treatment (n= 4 x 5 = 20) is large enough for null model analysis and should have enough statistical power to observe the warming effect on soil community assembly processes, which is supported by our results. Details are in the previous paper (Guo et al. 2018. Nature Climate Change 8: 813-818).
Data collection	The empirical data were collected from the published paper (Guo et al. 2018. Nature Climate Change 8: 813-818), which were obtained by 16S rRNA gene sequencing on Illumina MiSeq platform and processed by our pipeline (http://zhoulab5.rccc.ou.edu:8080) built on Galaxy platform (Version 17.01) with UPARSE from USEARCH (Version 9.2). Simulated community data were generated with custom scripts (available from GitHub https://github.com/DaliangNing/iCAMP1) written by R (version 3.5.3).
Timing and spatial scale	The samples were taken yearly at the date of peak plant biomass (late September or early October), because it can ensure meaningful plant biomass measurement and be able to capture treatment effect on soil microbial communities, which has been proven by our previous study (Guo et al. 2018) . We used the samples taken from 2010 to 2014, because our previous study (Guo et al. 2018) has found the experimental warming showed significant effect on soil microbial community structure within this time scale. The 4 controls and 4 warming plots are within the same site with a total area of around 600 square meters, and each plot is 2.5 m in length and 1.75 m in width. We set this spatial scale because the plot should be big enough to have diverse grass species and the site area should make all plots under very similar environment conditions except the different experimental treatments. Other details have been described in the previous published paper (Guo et al. 2018. Nature Climate Change 8: 813-818).
Data exclusions	No data were excluded.
Reproducibility	Each treatment has 4 replicate plots within the same site, and yearly sampled for 5 years. 16S rRNA gene amplicons were sequenced by MiSeq platform (Illumina, SanDiego, CA, USA) using a 500-cycle v2 MiSeq reagent cartridge (Illumina).
Randomization	The 8 plots (control and warming) are randomly distributed in the experiment site. Besides, all samples were randomly reordered and further analyzed in this random order, in order to rule out as many biases by the following sampling handling, DNA extraction, PCR amplification and sequencing as possible.
Blinding	Samples were in random order during processing. People who processed the samples to determine 16S rRNA sequences and physical chemical properties did not know the actual meaning of the sample IDs.

Did the study involve field work? ☒ Yes ☐ No

Field work, collection and transport

Field conditions	This study uses data from the previous published paper (Guo et al. 2018. Nature Climate Change 8: 813-818). The experimental site is in an old-field tallgrass prairie abandoned from cropping 40 years ago with light grazing until 2008. Dominant plants in this field site are C3 forbs (Ambrosia trifida, Solanum carolinense and Euphorbia dentate) and C4 grasses (Tridens flavus, Sporobolus compositus and Sorghum halapense). Mean monthly temperature in the field ranged from 3.3 °C in January to 28.1 °C in July, with an annual mean temperature of 16.3 °C. The average annual precipitation was 914 mm. In the experiment plots, the soil is Port-Pulaski-Keokuk complex, which is loam with 51% of sand, 35% of silt and 13% of clay. The soil has a high available water holding capacity (37%), neutral pH, and a deep (ca. 70 cm), moderately penetrable root zone. The concentrations of soil organic matter and total nitrogen (N)

	are 1.9% and 0.1%, respectively, and the soil bulk density is 1.2 g cm.
Location	The site is at the Kessler Atmospheric and Ecological Field Station (KAEFS) in the US Great Plains in McClain County, Oklahoma (34°59′ N, 97°31′W)
Access & import/export	This study uses data from the previous published paper (Guo et al. 2018. Nature Climate Change 8: 813-818). In the previous study, project and class site use requests were completed for our study. Liability waivers were completed hard copies provided to KAEFS.
Disturbance	In the experiment site, infrared heaters and rainfall-collection-redistribution devices may disturb the grassland ecosystem. To minimize the difference caused by disturbances, 'dummy' heaters/devices were used in the control plots. The moderate disturbance was kept within each plot (2.5x1.75m).

Reporting for specific materials, systems and methods

We require information from authors about some types of materials, experimental systems and methods used in many studies. Here, indicate whether each material, system or method listed is relevant to your study. If you are not sure if a list item applies to your research, read the appropriate section before selecting a response.

Materials & experimental systems

n/a	Involved in the study
☒	☐ Antibodies
☒	☐ Eukaryotic cell lines
☒	☐ Palaeontology and archaeology
☒	☐ Animals and other organisms
☒	☐ Human research participants
☒	☐ Clinical data
☒	☐ Dual use research of concern

Methods

n/a	Involved in the study
☒	☐ ChIP-seq
☒	☐ Flow cytometry
☒	☐ MRI-based neuroimaging
