# Supplementary material for: A quantitative framework reveals ecological drivers of grassland microbial community assembly in response to warming
Source: Nat Commun. 2020 Sep 18;11:4717. doi: 10.1038/s41467-020-18560-z (PMC7501310; doi:10.1038/s41467-020-18560-z)
Supplement: Supplementary file 4 — Description of Additional Supplementary Files [file 41467_2020_18560_MOESM4_ESM.pdf]

Description of Additional Supplementary Files

File Name: Supplementary Code 1
Description: iCAMP package and an example with detailed notes.
