# Supplementary material for: A quantitative framework reveals ecological drivers of grassland microbial community assembly in response to warming
Source: Nat Commun. 2020 Sep 18;11:4717. doi: 10.1038/s41467-020-18560-z (PMC7501310; doi:10.1038/s41467-020-18560-z)
Supplement: Supplementary file 5 — Supplementary Code 1 [file 41467_2020_18560_MOESM5_ESM.zip › iCAMP_1.2.4.zip!/iCAMP/html/00Index.html]

R: Infer Community Assembly Mechanisms by Phylogenetic-Bin-Based
Null Model Analysis

# Infer Community Assembly Mechanisms by Phylogenetic-Bin-Based Null Model Analysis

---

## Documentation for package ‘iCAMP’ version 1.2.4

- DESCRIPTION file.

## Help Pages

|  |  |
| --- | --- |
| iCAMP-package | Infer Community Assembly Mechanisms by Phylogenetic-bin-based null model analysis |
| bmntd | beta mean nearest taxon distance (betaMNTD) |
| bmpd | Beta mean pairwise distance (betaMPD) |
| bNRI.bin.big | Calculate beta net relatedness index (betaNRI) for each phylogenetic bin |
| bNRIn.p | Calculate beta net relatedness index with parallel computing |
| bNTI.bin.big | Calculate beta nearest taxon index (betaNTI) for each phylogenetic bin |
| bNTIn.p | Calculate beta nearest taxon index (betaNTI) with parallel computing |
| change.sigindex | Change significance index option in iCAMP analysis |
| cohend | Cohen's d effect size |
| dist.3col | Transform distance matrix to 3-column matrix |
| dist.bin.3col | Convert a list of dist (or matrixes) to a matrix |
| dniche | Calculate niche difference between species |
| example.data | A simple example dataset for test |
| iCAMP | Infer Community Assembly Mechanisms by Phylogenetic-bin-based null model analysis |
| icamp.big | Infer community assembly mechanism by phylogenetic-bin-based null model analysis |
| icamp.bins | Summarize iCAMP result in each bin |
| icamp.boot | Bootstrapping analysis of icamp results |
| icamp.out | Example output of function icamp.big |
| match.2col | Check the consistency of the first two columns of different matrixes |
| match.name | Check and ensure the consistency of IDs in different objects. |
| maxbigm | Find maximum value in a big matrix |
| midpoint.root.big | Midpoint root a large phylogeny |
| mntdn | Mean nearest taxon distance (MNTD) |
| mpdn | Mean pairwise distance (MPD) |
| NRI.p | Calculate net relatedness index (NRI) by parallel computing. |
| NTI.p | Calculate nearest taxon index (NTI) with parallel computing |
| null.norm | Normality test for null values |
| pdist.big | Pairwise phylogenetic distance matrix from big tree |
| ps.bin | Test within-bin phylogenetic signal |
| qp.bin.js | Calculate relative importance of community assembly processes |
| qpen | Quantifying assembly processes based on entire-community null model analysis |
| RC.bin.bigc | Calculate modified Roup-Crick index based on Bray-Curtis similarity for each phylogenetic bin |
| RC.pc | Modified Raup-Crick index based on Bray-Curtis similarity |
| snm | Estimation of neutral taxa percentae and dispersal rate |
| snm.boot | Estimation of neutral taxa percentae and dispersal rate |
| snm.comm | Estimation of neutral taxa percentae and dispersal rate |
| taxa.binphy.big | Phylogenetic binning based on phylogenetic tree |
| tree.droot | Distance from root to tip(s) and node(s) on phylogenetic tree |
| tree.path | List nodes and edge lengthes from root to each tip and/or node |
