# Supplementary material for: A quantitative framework reveals ecological drivers of grassland microbial community assembly in response to warming
Source: Nat Commun. 2020 Sep 18;11:4717. doi: 10.1038/s41467-020-18560-z (PMC7501310; doi:10.1038/s41467-020-18560-z)
Supplement: Supplementary file 5 — Supplementary Code 1 [file 41467_2020_18560_MOESM5_ESM.zip › iCAMP_1.2.4.zip!/iCAMP/html/bmntd.html]

R: beta mean nearest taxon distance (betaMNTD)

|  |  |
| --- | --- |
| bmntd {iCAMP} | R Documentation |

## beta mean nearest taxon distance (betaMNTD)

### Description

Calculates beta MNTD (beta mean nearest taxon distance, Webb et al 2008) for taxa in each pair of communities in a givern community matrix.

### Usage

```
bmntd(comm, pd, abundance.weighted = TRUE,
      exclude.conspecifics = FALSE,time.output=FALSE,
      unit.sum=NULL)
```

### Arguments

|  |  |
| --- | --- |
| `comm` | matrix or data.frame, community data matrix, rownames are sample names, colnames are OTU ids. |
| `pd` | matrix, pairwise phylogenetic distance matrix. |
| `abundance.weighted` | logic, whether weighted by species abundance, default is TRUE, means weighted. |
| `exclude.conspecifics` | logic, whether conspecific taxa in different communities be exclude from beta MNTD calculations, default is FALSE. |
| `time.output` | logic, whether to count calculation time, default is FALSE. |
| `unit.sum` | NULL or a number or a nemeric vector. When unit.sum is not NULL and a beta diversity index is calculated for a bin, the taxa abundances will be divided by unit.sum to calculate the relative abundances. usually, unit.sum can be set as the sequencing depth in each sample. Default setting is NULL, means not to do this special transformation. |

### Details

beta mean nearest taxon distance for taxa in each pair of communities. Modified from 'comdistnt' in package 'picante'(Kembel et al 2010), this function includes matrix multiplication to be efficient for medium size dataset.

### Value

result is a distance object of pairwise beta MNTD between samples.

### Note

Version 3: 2020.8.16, add examples.
Version 2: 2018.10.15, add unit.sum option. if unit.sum!=NULL, will calculate relative abundance according to unit.sum.
Version 1: 2015.9.23

### Author(s)

Daliang Ning

### References

Webb, C.O., Ackerly, D.D. & Kembel, S.W. (2008). Phylocom: software for the analysis of phylogenetic community structure and trait evolution. Bioinformatics, 24, 2098-2100.

Kembel, S.W., Cowan, P.D., Helmus, M.R., Cornwell, W.K., Morlon, H., Ackerly, D.D. et al. (2010). Picante: R tools for integrating phylogenies and ecology. Bioinformatics, 26, 1463-1464.

### See Also

`bNTIn.p`

### Examples

```
data("example.data")
comm=example.data$comm
pd=example.data$pd
bmntd.wt=bmntd(comm, pd, abundance.weighted = TRUE,
               exclude.conspecifics = FALSE)
```

---

[Package *iCAMP* version 1.2.4 Index]
