# Supplementary material for: A quantitative framework reveals ecological drivers of grassland microbial community assembly in response to warming
Source: Nat Commun. 2020 Sep 18;11:4717. doi: 10.1038/s41467-020-18560-z (PMC7501310; doi:10.1038/s41467-020-18560-z)
Supplement: Supplementary file 5 — Supplementary Code 1 [file 41467_2020_18560_MOESM5_ESM.zip › iCAMP_1.2.4.zip!/iCAMP/html/bmpd.html]

R: Beta mean pairwise distance (betaMPD)

|  |  |
| --- | --- |
| bmpd {iCAMP} | R Documentation |

## Beta mean pairwise distance (betaMPD)

### Description

Calculates mean pairwise distance separating taxa in each pair of communities in a given community matrix.

### Usage

```
bmpd(comm, pd, abundance.weighted = TRUE, na.zero = TRUE,
     time.output = FALSE, unit.sum = NULL)
```

### Arguments

|  |  |
| --- | --- |
| `comm` | matrix or data.frame, community data matrix, rownames are sample names, colnames are OTU ids. |
| `pd` | matrix, pairwise phylogenetic distance matrix. |
| `abundance.weighted` | logic, whether weighted by species abundance, default is TRUE, means weighted. |
| `na.zero` | logic. when the sum of a row (a sample) is zero in community data matrix, the relative abundance will be NAN. Sometimes, to avoid some problem in following calculation, this kind of NAN value need be set as zero. Defalt is TRUE. |
| `time.output` | logic, whether to count calculation time, default is FALSE. |
| `unit.sum` | When a beta diversity index is calculated for a bin, the taxa abundances will be divided by unit.sum to calculate the relative abundances. usually, unit.sum can be set as the sequencing depth in each sample. Default setting is NULL, means not to do this special transformation. |

### Details

beta mean pairwise distance.

### Value

Output is a distance object of pairwise betaMPD between samples.

### Note

Version 3: 2020.8.16, add examples.
Version 2: 2018.10.3, add unit.sum option. if unit.sum!=NULL, will calculate relative abundance according to unit.sum
Version 1: 2015.8.21.

### Author(s)

Daliang Ning

### References

Webb CO, Ackerly DD, and Kembel SW. 2008. Phylocom: software for the analysis of phylogenetic community structure and trait evolution. Bioinformatics 18:2098-2100

### See Also

`bNRIn.p`

### Examples

```
data("example.data")
comm=example.data$comm
pd=example.data$pd
bmpd.wt=bmpd(comm, pd, abundance.weighted = TRUE)
```

---

[Package *iCAMP* version 1.2.4 Index]
