# Supplementary material for: A quantitative framework reveals ecological drivers of grassland microbial community assembly in response to warming
Source: Nat Commun. 2020 Sep 18;11:4717. doi: 10.1038/s41467-020-18560-z (PMC7501310; doi:10.1038/s41467-020-18560-z)
Supplement: Supplementary file 5 — Supplementary Code 1 [file 41467_2020_18560_MOESM5_ESM.zip › iCAMP_1.2.4.zip!/iCAMP/html/bNTIn.p.html]

R: Calculate beta nearest taxon index (betaNTI) with parallel...

|  |  |
| --- | --- |
| bNTIn.p {iCAMP} | R Documentation |

## Calculate beta nearest taxon index (betaNTI) with parallel computing

### Description

Perform null model test based on a phylogenetic beta diversity index, beta mean phylogenetic distance to the nearest taxon (betaMNTD); calculate beta nearest taxon index (betaNTI), or modified Raup-Crick metric, or confidence level, based on the comparison between observed and null betaMNTD. Run by parallel computing.

### Usage

```
bNTIn.p(comm, dis, nworker = 4, memo.size.GB = 50,
        weighted = c(TRUE, FALSE), exclude.consp = FALSE,
        rand = 1000, output.bMNTD = c(FALSE, TRUE), 
        sig.index=c("SES","Confidence","RC","bNTI"),
        unit.sum = NULL, correct.special = FALSE,
        detail.null=FALSE,
        special.method=c("MNTD", "MPD", "both"),
        ses.cut=1.96,rc.cut=0.95,conf.cut=0.975)
```

### Arguments

|  |  |
| --- | --- |
| `comm` | community data matrix. rownames are sample names. colnames are species names |
| `dis` | Phylogenetic distance matrix. |
| `nworker` | for parallel computing. Either a character vector of host names on which to run the worker copies of R, or a positive integer (in which case that number of copies is run on localhost). default is 4, means 4 threads will be run. |
| `memo.size.GB` | numeric, to set the memory size as you need, so that calculation of large tree will not be limited by physical memory. unit is Gb. default is 50Gb. |
| `weighted` | Logic, consider abundances or not (just presence/absence). default is TRUE. |
| `exclude.consp` | Logic, should conspecific taxa in different communities be exclude from MNTD calculations? default is FALSE. The same as in the function bmntd. |
| `rand` | integer, randomization times. default is 1000. |
| `output.bMNTD` | logic, if TRUE, the output will include the observed betaMNTD. |
| `sig.index` | character, the index for null model significance test. SES or bNTI, standard effect size, i.e. beta nearest taxon index (betaNTI); Confidence, percentage of null values less extreme than the observed value, i.e. non-parametric one-side confidence level; RC, modified Raup-Crick index (RC) based on betaMNTD, i.e. count the number of null betaMNTD lower than observed betaMNTD plus a half of the number of null betaMNTD equal to observed betaMNTD, to get alpha, then calculate betaMNTD-based RC as (2 x alpha - 1). default is SES. If input a vector, only the first element will be used. |
| `unit.sum` | NULL or a number or a nemeric vector. When a beta diversity index is calculated for a bin, the taxa abundances will be divided by unit.sum to calculate the relative abundances. Usually, unit.sum can be set as the sequencing depth in each sample. Default setting is NULL, means not to do this transformation. |
| `correct.special` | logic, whether to correct the special cases when calculating bNTI. Default is FALSE. |
| `detail.null` | logic, if TRUE, the output will include all the null values. Default is FALSE. |
| `special.method` | When correct.special is TRUE, which method will be used to check underestimation of deterministic pattern(s) in special cases. MNTD, use null model test of mean distance to the nearest taxon; MPD, use null model test based on mean pairwise distance; both, use null model test of both MPD and MNTD. Default is MNTD. |
| `ses.cut` | numeric, the cutoff of significant standard effect size, default is 1.96. |
| `rc.cut` | numeric, the cutoff of significant modified Raup-Crick metric, default is 0.95. |
| `conf.cut` | numeric, the cutoff of significant one-side confidence level, default is 0.975. |

### Details

The beta nearest taxon index (beta NTI; Webb et al. 2008, Stegen et al. 2012) is a standardized measure of the mean phylogenetic distance to the nearest taxon between samples/communities (beta MNTD) and quantifies the extent of terminal clustering, independent of deep level clustering. Parallel computing is used to improve the speed.

The null model algorithm is "taxa shuffle" (Kembel 2009), i.e. shuffling taxa labels across the
tips of the phylogenetic tree to randomize phylogenetic relationships among species.

In the output of betaNTI, the diagonal are set as zero. If the randomized results are all the same, the standard deviation will be zero and betaNTI will be NAN. In this case, beta NTI will be set as zero, since the observed result is not differentiable from randomized results.

Modified RC (Chase et al 2011) and Confidence (Ning et al 2020) are alternative significance test indexes to evaluate how the observed beta diversity index deviates from null expectation, which could be a better metric than standardized effect size (betaNTI) in some cases, e.g. null values do not follow normal distribution.

### Value

Output is a list with following elements:

|  |  |
| --- | --- |
| `index` | a square matrix of betaNTI (or RC or Confidence based on betaMNTD) values. |
| `sig.index` | character, indicates the index for null model significance test, SES (i.e. betaNTI), RC, or Confidence. |
| `betaMNTD.obs` | Output only if output.bMNTD is TRUE. A square matrix of observed beta MNTD values. |
| `rand` | Output only if detail.null is TRUE. A matrix with null values of beta MNTD for each turnover. |
| `special.crct` | Output only if detail.null is TRUE. it will be NULL if correct.special is FALSE. Otherwise, it will be a list with three elements, corresponding to three different null model significance testing indexes, i.e. SES, RC, and Confidence. Each element is a square matrix, where the value is zero if the result for a turnover does not need to correct, otherwise there will be a corrected value. |

### Note

Version 6: 2020.8.18, update help document, add example.
Version 5: 2020.8.1, change RC opiton to sig.index, add detail.null, rc.cut and conf.cut.
Version 4: 2018.10.15, consider special cases.
Version 3: 2016.3.26, add RC option.
Version 2: 2015.9.23, set diag of bNTI = 0 and set 0/0 = 0 for bNTI.
Version 1: 2015.4.1

### Author(s)

Daliang Ning

### References

Webb, C.O., Ackerly, D.D. & Kembel, S.W. (2008). Phylocom: software for the analysis of phylogenetic community structure and trait evolution. Bioinformatics, 24, 2098-2100.

Kembel, S.W. (2009). Disentangling niche and neutral influences on community assembly: assessing the performance of community phylogenetic structure tests. Ecol Lett, 12, 949-960.

Stegen, J.C., Lin, X., Konopka, A.E. & Fredrickson, J.K. (2012). Stochastic and deterministic assembly processes in subsurface microbial communities. Isme Journal, 6, 1653-1664.

Chase, J.M., Kraft, N.J.B., Smith, K.G., Vellend, M. & Inouye, B.D. (2011). Using null models to disentangle variation in community dissimilarity from variation in alpha-diversity. Ecosphere, 2, 1-11.

Ning, D., Yuan, M., Wu, L., Zhang, Y., Guo, X., Zhou, X. et al. (2020). A quantitative framework reveals the ecological drivers of grassland soil microbial community assembly in response to warming. bioRxiv. doi:10.1101/2020.02.22.960872 (Nature Communications, final revision).

### See Also

`bmntd`

### Examples

```
data("example.data")
comm=example.data$comm
pd=example.data$pd
nworker=2 # parallel computing thread number
rand.time=4 # usually use 1000 for real data.
bNTI=bNTIn.p(comm=comm, dis=pd, nworker = nworker, memo.size.GB = 50,
             weighted = TRUE, exclude.consp = FALSE, rand = rand.time,
             output.bMNTD = FALSE, sig.index = "SES", unit.sum = NULL,
             correct.special = TRUE, detail.null = FALSE,
             special.method = "MNTD")
```

---

[Package *iCAMP* version 1.2.4 Index]
