# Supplementary material for: A quantitative framework reveals ecological drivers of grassland microbial community assembly in response to warming
Source: Nat Commun. 2020 Sep 18;11:4717. doi: 10.1038/s41467-020-18560-z (PMC7501310; doi:10.1038/s41467-020-18560-z)
Supplement: Supplementary file 5 — Supplementary Code 1 [file 41467_2020_18560_MOESM5_ESM.zip › iCAMP_1.2.4.zip!/iCAMP/html/change.sigindex.html]

R: Change significance index option in iCAMP analysis

|  |  |
| --- | --- |
| change.sigindex {iCAMP} | R Documentation |

## Change significance index option in iCAMP analysis

### Description

This function is to change the method to calculate significance between null and observed dissimilarity and/or change the significance threshold values.

### Usage

```
change.sigindex(icamp.output, sig.index = c("Confidence", "SES.RC", "SES", "RC"),
                detail.save = TRUE, detail.null = FALSE,
                ses.cut = 1.96, rc.cut = 0.95, conf.cut = 0.975)
```

### Arguments

|  |  |
| --- | --- |
| `icamp.output` | list, the exact output of the function icamp.big in which detail.null must be TRUE, to save all null values. |
| `sig.index` | character, Confidence means to directly count the percentage of null values higher/lower than observed value; SES.RC means to use Standard Effect Size (e.g. betaNRI, betaNTI) for phylogenetic beta diversity and use modified Raup-Crick for taxonomic beta diversity, which is typical practice in the previous method; SES means to use Standard Effect Size for both phylogenetic and taxonomic beta diversity; RC means to use modified Raup-Crick for both phylogenetic and taxonomic beta diversity. |
| `detail.save` | logic, whether to output the details, including binning information, significance indexes, bin abundances, and some key parameter settings for iCAMP analysis. Default is TRUE |
| `detail.null` | logic, whether to output all observed and null values of beta diversity indexes. Default is FALSE. |
| `ses.cut` | numeric, the cutoff of significant standard effect size, default is 1.96. |
| `rc.cut` | numeric, the cutoff of significant modified Raup-Crick index value, default is 0.95. |
| `conf.cut` | numeric, the cutoff of significant confidence level (one-tail), default is 0.975. |

### Details

This function is to re-calculate significance using another index or a different threshold value using previously saved null model values. Since the null values are directly extracted from previous icamp.big results, it can skip the most time-consuming step (randomization) and quickly complete calculation.

The default threshold values of Confidence (0.975), SES (1.96), and RC (0.95) mean to capture the 0.95 two-tail confidence level (P=0.05). But, SES need to assume the null values follow normal distribution. RC counts in a half of the special cases that null values are equal to observed values, which is good for obtaining a symmetric metric but theoretically has risk to misestimate significance level (but very slight). Thus, Confidence is preferred as long as the 1000-time randomization is representative.

### Value

The output will be the same as icamp.big.

### Note

Version 2: 2020.8.18, update help document, add example.
Version 1: 2020.8.1

### Author(s)

Daliang Ning

### References

Ning, D., Yuan, M., Wu, L., Zhang, Y., Guo, X., Zhou, X. et al. (2020). A quantitative framework reveals the ecological drivers of grassland soil microbial community assembly in response to warming. bioRxiv. doi:10.1101/2020.02.22.960872 (Nature Communications, final revision).

### See Also

`icamp.big`, `null.norm`

### Examples

```
data("icamp.out")
icamp.out.new=change.sigindex(icamp.output=icamp.out, sig.index = "Confidence")
```

---

[Package *iCAMP* version 1.2.4 Index]
