# Supplementary material for: A quantitative framework reveals ecological drivers of grassland microbial community assembly in response to warming
Source: Nat Commun. 2020 Sep 18;11:4717. doi: 10.1038/s41467-020-18560-z (PMC7501310; doi:10.1038/s41467-020-18560-z)
Supplement: Supplementary file 5 — Supplementary Code 1 [file 41467_2020_18560_MOESM5_ESM.zip › iCAMP_1.2.4.zip!/iCAMP/html/cohend.html]

R: Cohen's d effect size

|  |  |
| --- | --- |
| cohend {iCAMP} | R Documentation |

## Cohen's d effect size

### Description

This function is to calculate the popular effect size index Cohen's d.

### Usage

```
cohend(treat, control, paired = FALSE)
```

### Arguments

|  |  |
| --- | --- |
| `treat` | a numeric vector. treatment group. |
| `control` | a numberic vector. control group. |
| `paired` | logic. Whether the samples in treatment and control groups are paired. default is FALSE. |

### Details

This function computes the value of Cohen's d statistics (Cohen 1988).
The effect size magnitude is performed using the thresholds proposed by Cohen (1992), i.e. |d|<0.2 "negligible", 0.2<=|d|<0.5 "small", 0.5<=|d|<0.8 "medium", |d|>=0.8 "large".
The variance of the d is calculate using the conversion formula reportead at page 238 of Cooper et al. (2009):
((n1+n2)/(n1\*n2) + .5\*d^2/df) \* ((n1+n2)/df)
Its square root is output as standard deviation of d.

### Value

A list of values will be returned

|  |  |
| --- | --- |
| `d` | Cohen's d value, (mean(treat)-mean(control))/sd |
| `sd` | standard deviation of d |
| `magnitude` | a qualitative assessment of the magnitude of effect size |
| `paired` | whether the samples are paired |

### Note

version 1: 2016.2.12

### Author(s)

Daliang Ning

### References

Cohen, J. (1988). Statistical power analysis for the behavioral sciences (2nd ed.). New York:Academic Press

Cohen, J. (1992). A power primer. Psychological Bulletin, 112, 155-159.

The Handbook of Research Synthesis and Meta-Analysis (Cooper, Hedges, & Valentine, 2009)

### Examples

```
x=c(1,5,8)
y=c(2,6,10)
cohend(x,y)
cohend(x,y,paired=TRUE)
```

---

[Package *iCAMP* version 1.2.4 Index]
