# Supplementary material for: A quantitative framework reveals ecological drivers of grassland microbial community assembly in response to warming
Source: Nat Commun. 2020 Sep 18;11:4717. doi: 10.1038/s41467-020-18560-z (PMC7501310; doi:10.1038/s41467-020-18560-z)
Supplement: Supplementary file 5 — Supplementary Code 1 [file 41467_2020_18560_MOESM5_ESM.zip › iCAMP_1.2.4.zip!/iCAMP/html/dist.3col.html]

R: Transform distance matrix to 3-column matrix

|  |  |
| --- | --- |
| dist.3col {iCAMP} | R Documentation |

## Transform distance matrix to 3-column matrix

### Description

Transform a distance matrix to a 3-column matrix in which the first 2 columns indicate the pairwised samples/species names.

### Usage

```
dist.3col(dist)
```

### Arguments

|  |  |
| --- | --- |
| `dist` | a square matrix or distance object with column names and row names. |

### Details

In many cases, a 3-column matrix is easier to use than a distance matrix.

### Value

|  |  |
| --- | --- |
| `name1` | 1st column, the first item of pairwised two items |
| `name2` | 2nd column, the second item of pairwised two items |
| `dis` | 3rd column, distance value of the pairwised two itmes |

### Note

Version 1: 2015.5.17

### Author(s)

Daliang Ning

### Examples

```
# In this example, dist.3col transforms the distance object
# of Bray-Curtis dissimilarity to 3-column matrix.

data("example.data")
comm=example.data$comm
BC=vegan::vegdist(comm)
BC3c=dist.3col(BC)
```

---

[Package *iCAMP* version 1.2.4 Index]
