# Supplementary material for: A quantitative framework reveals ecological drivers of grassland microbial community assembly in response to warming
Source: Nat Commun. 2020 Sep 18;11:4717. doi: 10.1038/s41467-020-18560-z (PMC7501310; doi:10.1038/s41467-020-18560-z)
Supplement: Supplementary file 5 — Supplementary Code 1 [file 41467_2020_18560_MOESM5_ESM.zip › iCAMP_1.2.4.zip!/iCAMP/html/dist.bin.3col.html]

R: Convert a list of dist (or matrixes) to a matrix

|  |  |
| --- | --- |
| dist.bin.3col {iCAMP} | R Documentation |

## Convert a list of dist (or matrixes) to a matrix

### Description

Convert a list of distance matrixes (or square matrixes) with the same sample IDs into a matrix.

### Usage

```
dist.bin.3col(dist.bin, obj.name = NULL)
```

### Arguments

|  |  |
| --- | --- |
| `dist.bin` | a list, each element is a distance matrix or square matrix. all elements have exactly the same sample IDs (rownames and colnames) which are in the same order. |
| `obj.name` | a character, as a prefix of the bin names. |

### Details

A tool to facilitate format transformation in iCAMP analysis.

### Value

output is a matrix. The first two columns are sample IDs, and each of the following columns represent an element in the original list which usually is a bin in iCAMP analysis.

### Note

Version 2: 2020.8.18, add example
Version 1: 2015.8.30

### Author(s)

Daliang Ning

### Examples

```
# let's see a very simple example
bin.dist=as.matrix(dist(1:10))
rownames(bin.dist)<-colnames(bin.dist)<-paste0("Sample",1:10)
dist.bins=list(bin1=bin.dist,bin2=bin.dist+1,bin3=bin.dist*2)
dis.3c=dist.bin.3col(dist.bins,obj.name="test")
```

---

[Package *iCAMP* version 1.2.4 Index]
