# Supplementary material for: A quantitative framework reveals ecological drivers of grassland microbial community assembly in response to warming
Source: Nat Commun. 2020 Sep 18;11:4717. doi: 10.1038/s41467-020-18560-z (PMC7501310; doi:10.1038/s41467-020-18560-z)
Supplement: Supplementary file 5 — Supplementary Code 1 [file 41467_2020_18560_MOESM5_ESM.zip › iCAMP_1.2.4.zip!/iCAMP/html/dniche.html]

R: Calculate niche difference between species

|  |  |
| --- | --- |
| dniche {iCAMP} | R Documentation |

## Calculate niche difference between species

### Description

Calculate niche difference between species based on each environmental variable, directly output the matrix or save the result matrix as big.matrix.

### Usage

```
dniche(env, comm,
       method = c("ab.overlap", "niche.value", "prefer.overlap"),
       nworker = 4, memory.G = 50, out.dist = FALSE,
       bigmemo = TRUE, nd.wd = getwd())
```

### Arguments

|  |  |
| --- | --- |
| `env` | matrix or data.frame, each row is a sample, each column is an environmental factor which may be important to represent the niche, thus rownames are sample IDs, and colnames are environmental factor names. |
| `comm` | matrix or data.frame, each row is a sample, each column is a spcies (OTU or ASV), thus rownames are sample IDs, colnames are species/OTU/ASV IDs. |
| `method` | methods to calculate niche difference. ab.overlap means to calculate from overlapp based on observed abundances along an environment gradient. niche.value means to calculate the difference from abundance weighted mean of each environment factor for each species. prefer.overlap is similar to ab.overlap, but the observed abundances of each species are divided by total abundance sum of the species before calculating overlapping. If list multiple methods as a vector, only the first element will be used. |
| `nworker` | integer, for parallel computing. Either a character vector of host names on which to run the worker copies of R, or a positive integer (in which case that number of copies is run on localhost). default is 4, means 4 threads will be run. |
| `memory.G` | numeric, to set the memory size as you need, so that calculation of large tree will not be limited by physical memory. unit is Gb. default is 50Gb. |
| `out.dist` | logic, if TRUE, the output niche difference matrix of each environment factor will be a distance object, otherwise will be a matrix in the output list. |
| `bigmemo` | logic, if TRUE, big.matrix in R package bigmemory will be used to save each niche differnece matrix as a big matrix on hard disk. |
| `nd.wd` | folder path, when bigmemo is TRUE, where the big matrixes are saved. |

### Details

The method niche.value is to calculate niche difference as the absolute difference of niche values between each pair of species. The niche value of a species is calculated as abundance-weighted mean of each environmental factor as previously reported (Stegen et al 2012 ISME J). In the method ab.overlap, the abundance of each species along the gradient of an environment factor is estimated using the density function using Gaussian kernel with 512 points. Then, the niche difference between two species is calculated as the sum of absolute abundance difference at each point divided by the sum of the higher abundance at each point, like Ruzicka dissimilarity (weighted Jaccard). It is like 1 - niche overlap based on abundance profile overlap, thus called ab.overlap. The method prefer.overlap is very similar to ab.overlap, just one modification, i.e. the observed abundance of each species in each sample is divied by the total abundance of the species across all sample, to normalize the profile, before calcuating niche difference.

Bigmemory (Kane et al 2013) is used to deal with large datasets.

### Value

The output is a list object, with several elements.

|  |  |
| --- | --- |
| `bigmemo` | logic, to show whether big.matrix is used. |
| `nd` | if bigmemo is FALSE, this is a list of matrixes or distance objects showing the niche difference matrix based on each environment factor. if bigmemo is TRUE, this is a list of big matrix file names. |
| `nd.wd` | only appear when bigmemo is TRUE, shows the folder path where the big matrixes are saved. |
| `names` | only appear when bigmemo is TRUE, shows species (OTU or ASV) IDs, in the same order as rownames and colnames in the niche difference matrixes. |
| `method` | The method used. |

### Note

Version 2: 2020.8.18, add example.
Version 1: 2020.5.15

### Author(s)

Daliang Ning

### References

Stegen, J.C., Lin, X., Konopka, A.E. & Fredrickson, J.K. (2012). Stochastic and deterministic assembly processes in subsurface microbial communities. ISME J, 6, 1653-1664.

Ning, D., Yuan, M., Wu, L., Zhang, Y., Guo, X., Zhou, X. et al. (2020). A quantitative framework reveals the ecological drivers of grassland soil microbial community assembly in response to warming. bioRxiv. doi:10.1101/2020.02.22.960872 (Nature Communications, final revision).

Kane, M.J., Emerson, J., Weston, S. (2013). Scalable Strategies for Computing with Massive Data. Journal of Statistical Software, 55(14), 1-19. URL http://www.jstatsoft.org/v55/i14/.

### See Also

`ps.bin`

### Examples

```
data("example.data")
comm=example.data$comm
env=example.data$env

# if data is small, you do not need to use big.memory
niche.dif=dniche(env = env, comm = comm, method = "niche.value",
                 nworker = 1,out.dist=FALSE,bigmemo=FALSE,nd.wd = NULL)

# if data is large, you need to use big.memory
# since big.memory need to specify a certain folder,
# it is set as 'not run'.
# but you may test the code on your computer after change the path for 'save.wd'.
## Not run: 
  save.wd="C:/test/" # please change to the folder you want to save the big niche difference matrix.
  nworker=2 # parallel computing thread number
  niche.dif=dniche(env = env, comm = comm,
                   method = "niche.value", nworker = nworker,
                   out.dist=FALSE,bigmemo=TRUE,nd.wd = save.wd)

## End(Not run)
```

---

[Package *iCAMP* version 1.2.4 Index]
