# Supplementary material for: A quantitative framework reveals ecological drivers of grassland microbial community assembly in response to warming
Source: Nat Commun. 2020 Sep 18;11:4717. doi: 10.1038/s41467-020-18560-z (PMC7501310; doi:10.1038/s41467-020-18560-z)
Supplement: Supplementary file 5 — Supplementary Code 1 [file 41467_2020_18560_MOESM5_ESM.zip › iCAMP_1.2.4.zip!/iCAMP/html/example.data.html]

R: A simple example dataset for test

|  |  |
| --- | --- |
| example.data {iCAMP} | R Documentation |

## A simple example dataset for test

### Description

A small dataset including community matrix, phylogenetic tree, treatment information, environmental factors. just for test.

### Usage

```
data("example.data")
```

### Format

The format is:
List of 4
$ comm : int [1:20, 1:30] 1 3 0 0 2 2 2 0 0 4 ...
..- attr(\*, "dimnames")=List of 2
.. ..$ : chr [1:20] "s1" "s2" "s3" "s4" ...
.. ..$ : chr [1:30] "OTU1" "OTU2" "OTU3" "OTU4" ...
$ tree :List of 4
..$ edge : int [1:58, 1:2] 31 32 33 34 35 36 36 35 34 37 ...
..$ edge.length: num [1:58] 0.314 0.422 0.315 0.881 0.774 ...
..$ Nnode : int 29
..$ tip.label : chr [1:30] "OTU1" "OTU2" "OTU3" "OTU4" ...
..- attr(\*, "class")= chr "phylo"
..- attr(\*, "order")= chr "cladewise"
$ treat:'data.frame': 20 obs. of 2 variables:
..$ Management: chr [1:20] "SF" "BF" "SF" "SF" ...
..$ Location : chr [1:20] "south" "south" "south" "south" ...
$ env :'data.frame': 20 obs. of 2 variables:
..$ pH : num [1:20] 3.4 3.6 3.8 4 4.2 4.4 4.6 4.8 5 5.2 ...
..$ temperature: num [1:20] 12.9 4 13.7 6 8 2.7 10.4 3.2 7.6 5.8 ...
$ pd :'data.frame': 30 obs. of 30 variables:
..$ OTU1 : num [1:30] 0 0.606 1.348 3.015 3.331 ...
..$ OTU2 : num [1:30] 0.606 0 1.19 2.856 3.173 ...
..$ OTU3 : num [1:30] 1.35 1.19 0 2.05 2.37 ...
..$ OTU4 : num [1:30] 3.015 2.856 2.051 0 0.479 ...
..$ OTU5 : num [1:30] 3.331 3.173 2.367 0.479 0 ...
..$ OTU6 : num [1:30] 2.96 2.8 2 1.9 2.22 ...
..$ OTU7 : num [1:30] 3.62 3.46 2.65 2.56 2.87 ...
..$ OTU8 : num [1:30] 4.97 4.82 4.01 3.92 4.23 ...
..$ OTU9 : num [1:30] 4.77 4.61 3.81 3.71 4.03 ...
..$ OTU10: num [1:30] 3.96 3.81 3 2.9 3.22 ...
..$ OTU11: num [1:30] 3.4 3.24 2.43 2.34 2.65 ...
..$ OTU12: num [1:30] 3.69 3.53 2.73 2.63 2.95 ...
..$ OTU13: num [1:30] 5.64 5.48 4.68 4.58 4.9 ...
..$ OTU14: num [1:30] 6.1 5.94 5.14 5.04 5.36 ...
..$ OTU15: num [1:30] 4.73 4.57 3.77 3.67 3.99 ...
..$ OTU16: num [1:30] 5.77 5.61 4.8 4.71 5.03 ...
..$ OTU17: num [1:30] 5.97 5.81 5 4.91 5.22 ...
..$ OTU18: num [1:30] 5.27 5.11 4.3 4.21 4.52 ...
..$ OTU19: num [1:30] 7.56 7.4 6.6 6.5 6.82 ...
..$ OTU20: num [1:30] 7.55 7.39 6.58 6.49 6.8 ...
..$ OTU21: num [1:30] 6.49 6.33 5.53 5.43 5.75 ...
..$ OTU22: num [1:30] 6.52 6.37 5.56 5.46 5.78 ...
..$ OTU23: num [1:30] 6.68 6.52 5.72 5.62 5.94 ...
..$ OTU24: num [1:30] 6.35 6.19 5.38 5.29 5.61 ...
..$ OTU25: num [1:30] 6.37 6.21 5.41 5.31 5.63 ...
..$ OTU26: num [1:30] 5.73 5.57 4.77 4.67 4.99 ...
..$ OTU27: num [1:30] 6.23 6.07 5.27 5.17 5.49 ...
..$ OTU28: num [1:30] 5.99 5.83 5.02 4.93 5.24 ...
..$ OTU29: num [1:30] 5.7 5.54 4.74 4.64 4.96 ...
..$ OTU30: num [1:30] 3.94 3.78 2.97 2.88 3.19 ...
$ pdid.bin:List of 3
..$ : int [1:5] 1 2 3 4 5
..$ : int [1:7] 6 7 8 9 10 11 12
..$ : int [1:18] 13 14 15 16 17 18 19 20 21 22 ...
$ sp.bin :'data.frame': 30 obs. of 1 variable:
..$ bin.id.new: num [1:30] 1 1 1 1 1 2 2 2 2 2 ...
$ classification: chr [1:30, 1:6] "Archaea" "Archaea" "Archaea" "Archaea" ...
..- attr(\*, "dimnames")=List of 2
.. ..$ : chr [1:30] "OTU1" "OTU2" "OTU3" "OTU4" ...
.. ..$ : chr [1:6] "Domain" "Phylum" "Class" "Order" ...

### Details

comm is a matrix, each row as a sample, each column as a species.

tree means phylogenetic tree.

treat is a treatment information matrix, each row as a sample, each column indicates a type of treatment.

env is a matrix of environmental factors, i.e. pH and temperature in this case.

pd is a matrix of the pairwise phylogenetic distance between species.

pdid.bin is a list, each element is a vector of integer, indicating which rows/columns in the big phylogenetic matrix represent the taxa in a bin.

sp.bin is a one-column matrix, rownames are taxa IDs (i.e. OTU IDs), the only column shows the bin ID of each taxon. Bin IDs are integers in the same order as the elements in the list of pdid.bin.

classification is a matrix to define the lineage of each taxon.

This dataset is randomly generated, just for test.

### Examples

```
data(example.data)
comm=example.data$comm
tree=example.data$tree
treat=example.data$treat
env=example.data$env
pd=example.data$pd
pdid.bin=example.data$pdid.bin
sp.bin=example.data$sp.bin
```

---

[Package *iCAMP* version 1.2.4 Index]
