# Supplementary material for: A quantitative framework reveals ecological drivers of grassland microbial community assembly in response to warming
Source: Nat Commun. 2020 Sep 18;11:4717. doi: 10.1038/s41467-020-18560-z (PMC7501310; doi:10.1038/s41467-020-18560-z)
Supplement: Supplementary file 5 — Supplementary Code 1 [file 41467_2020_18560_MOESM5_ESM.zip › iCAMP_1.2.4.zip!/iCAMP/html/iCAMP-package.html]

R: Infer Community Assembly Mechanisms by Phylogenetic-bin-based...

|  |  |
| --- | --- |
| iCAMP-package {iCAMP} | R Documentation |

## Infer Community Assembly Mechanisms by Phylogenetic-bin-based null model analysis

### Description

This package is to implement a general framework to quantitatively infer Community Assembly Mechanisms by Phylogenetic-bin-based null model analysis, abbreviated as iCAMP (Ning et al 2020). It can quantitatively assess the relative importance of different community assembly processes, such as selection, dispersal, and drift, for both communities and each phylogenetic group ('bin'). Each bin usually consists of different taxa from a family or an order. The package also provides funcitons to implement some other published methods, including neutral taxa percentage (Burns et al 2016) based on neutral theory model (Sloan et al 2006) and quantifying assembly processes based on entire-community null models (Stegen et al 2013). It also includes quite a few handy functions, particularly for big datasets, such as phylogenetic and taxonomic null model analysis at both community and bin levels, between-taxa niche difference and phylogenetic distance calculation, phylogenetic signal test within phylogenetic groups, midpoint root of big trees, etc.

Version 1.2.4: the first formal version of iCAMP for CRAN.

### Details

|  |  |
| --- | --- |
| Package: | iCAMP |
| Type: | Package |
| Version: | 1.2.4 |
| Date: | 2020-08-23 |
| License: | GPL-2 |
|  |  |

### Author(s)

Daliang Ning <ningdaliang@ou.edu>

### References

Ning, D., Yuan, M., Wu, L., Zhang, Y., Guo, X., Zhou, X. et al. (2020). A quantitative framework reveals ecological drivers of grassland microbial community assembly in response to warming. bioRxiv. doi:10.1101/2020.02.22.960872 (Nature Communications, in revision).

Burns, A.R., Stephens, W.Z., Stagaman, K., Wong, S., Rawls, J.F., Guillemin, K. et al. (2016). Contribution of neutral processes to the assembly of gut microbial communities in the zebrafish over host development. Isme Journal, 10, 655-664.

Sloan, W.T., Lunn, M., Woodcock, S., Head, I.M., Nee, S. & Curtis, T.P. (2006). Quantifying the roles of immigration and chance in shaping prokaryote community structure. Environmental Microbiology, 8, 732-740.

Stegen, J.C., Lin, X., Fredrickson, J.K., Chen, X., Kennedy, D.W., Murray, C.J. et al. (2013). Quantifying community assembly processes and identifying features that impose them. Isme Journal, 7, 2069-2079.

### Examples

```
data("example.data")
comm=example.data$comm
tree=example.data$tree
# since need to save some outputs to a certain folder,
# the following code is set as 'not run'.
# but you may test the code on your computer
# after change the path for 'save.wd'.
## Not run: 
  save.wd="C:/test/" # please change to the folder you want to save the pd.big output.
  pd.wd=paste0(save.wd,"/pdbig")
  nworker=4 # parallel computing thread number
  rand.time=20 # usually use 1000 for real data.
  
  bin.size.limit=5 # for real data, usually use a proper number
  # according to phylogenetic signal test or try some settings
  # then choose the reasonable stochasticity level.
  # our experience is 12, or 24, or 48.
  # but for this example dataset which is too small, have to use 5.
  
  setwd(save.wd)
  icamp.out=icamp.big(comm=comm,tree=tree,pd.wd=pd.wd,
                      rand=rand.time, nworker=nworker,
                      bin.size.limit=bin.size.limit)

## End(Not run)
```

---

[Package *iCAMP* version 1.2.4 Index]
