# Supplementary material for: A quantitative framework reveals ecological drivers of grassland microbial community assembly in response to warming
Source: Nat Commun. 2020 Sep 18;11:4717. doi: 10.1038/s41467-020-18560-z (PMC7501310; doi:10.1038/s41467-020-18560-z)
Supplement: Supplementary file 5 — Supplementary Code 1 [file 41467_2020_18560_MOESM5_ESM.zip › iCAMP_1.2.4.zip!/iCAMP/html/icamp.big.html]

R: Infer community assembly mechanism by phylogenetic-bin-based...

|  |  |
| --- | --- |
| icamp.big {iCAMP} | R Documentation |

## Infer community assembly mechanism by phylogenetic-bin-based null model analysis

### Description

main function of iCAMP, to perform phylogenetic-bin-based null model analysis and quantify the relative importance of different processes.

### Usage

```
icamp.big(comm, tree, pd.desc = NULL, pd.spname = NULL, pd.wd = getwd(),
          rand = 1000, prefix = "iCAMP", ds = 0.2, pd.cut = NA, sp.check = TRUE,
          phylo.rand.scale = c("within.bin", "across.all", "both"),
          taxa.rand.scale = c("across.all", "within.bin", "both"),
          phylo.metric = c("bMPD", "bMNTD", "both", "bNRI", "bNTI"), 
          sig.index=c("Confidence","SES.RC","SES","RC"), bin.size.limit = 24,
          nworker = 4, memory.G = 50, rtree.save = FALSE, detail.save = TRUE,
          qp.save = TRUE, detail.null=FALSE, ignore.zero = TRUE,
          output.wd = getwd(), correct.special = TRUE, unit.sum = rowSums(comm),
          special.method = c("depend","MPD","MNTD","both"),
          ses.cut = 1.96, rc.cut = 0.95, conf.cut=0.975,
          omit.option = c("no", "test", "omit"), meta.ab = NULL)
```

### Arguments

|  |  |
| --- | --- |
| `comm` | matrix or data.frame, community data, each row is a sample or site, each colname is a taxon (a species or OTU or ASV), thus rownames should be sample IDs, colnames should be taxa IDs. |
| `tree` | phylogenetic tree, an object of class "phylo". |
| `pd.desc` | the name of the file to hold the backingfile description of the phylogenetic distance matrix, it is usually "pd.desc" if using default setting in pdist.big function. If it is NULL, the fucntion pd.big will be used to calculate the phylogenetic distance matrix from tree, and save it in pd.wd as a big.memory file. |
| `pd.spname` | character vector, taxa id in the same rank as the big matrix of phylogenetic distances. |
| `pd.wd` | folder path, where the bigmemmory file of the phylogenetic distance matrix are saved. |
| `rand` | integer, randomization times. default is 1000. |
| `prefix` | character string, the prefix of those output files. |
| `ds` | numeric, the general threshold of phylogenetic distance within which the phylogenetic signal is still significant. default is 0.2. |
| `pd.cut` | numeric, the distance to the tree root where the phylogenetic tree is trancated to get strict phylogenetic bins. if pd.cut is set, the distance threshold (ds) is disabled. default is NA. |
| `sp.check` | logic, whether to match the taxa ids in community data, phylogenetic distance matrix, and tree. default is TRUE. |
| `phylo.rand.scale` | character, the scale to randomize the taxa for phylogenetic null model. "within.bin" means randomization within each bin; "across.all" means randomization across all bins; "both" means to test both methods. Default setting is within.bin. |
| `taxa.rand.scale` | character, the scale to randomize the taxa for taxonomic null model. "within.bin" means randomization within each bin; "across.all" means randomization across all bins; "both" means to test both methods. Default setting is across.all. |
| `phylo.metric` | character, the metric for phylogenetic null model analysis. bMPD (or bNRI), null model analysis based on beta mean pairwise distance (betaMPD); if sig.index is SES, it is beta net relatedness index (betaNRI). bMNTD (or bNTI), null model analysis based on beta mean nearest taxon distance (betaMNTD); if sig.index is SES, it is beta nearest taxon index (betaNTI). both, use null model test based on both bMPD and bMNTD. Default setting is based on bMPD. |
| `sig.index` | character, the index for null model significance test. Confidence, percentage of null values less extreme than the observed value, i.e. non-parametric one-side confidence level; if set sig.index as Confidence, it will be applied to both phylogenetic and taxonomic metrics. If set as SES.RC, use standard effect size (SES) for phylogenetic metrics (i.e. betaNTI or betaNRI), and use modified Raup-Crick (RC) for taxonomic metrics (RCbray). If set as SES, use SES for both phylogenetic and taxonomic metrics. If set as RC, use RC for both phylogenetic and taxonomic metrics. default is Confidence. If input a vector, only the first one will be used. |
| `bin.size.limit` | integer, the minimal requirement of bin size (taxa numer in a bin). Default setting is 24. |
| `nworker` | integer, for parallel computing. Either a character vector of host names on which to run the worker copies of R, or a positive integer (in which case that number of copies is run on localhost). default is 4, means 4 threads will be run. |
| `memory.G` | numeric, to set the memory size as you need, so that calculation of large tree will not be limited by physical memory. unit is Gb. default is 50Gb. |
| `rtree.save` | logic, whether to save the rooted tree as nwk file, if the input tree is not rooted. Default is FALSE. |
| `detail.save` | logic, whether to save the details, i.e. some key objects for iCAMP analysis, as rda file. Default is TRUE. |
| `qp.save` | logic, whether to save the relative importance of processes as csv file. Default is TRUE. |
| `detail.null` | logic, if TRUE, the output will include all the null values. Default is FALSE. But this need to be TRUE if you want to change significance testing index later using 'change.sigindex'. |
| `ignore.zero` | logic, in the community data matrix (comm), whether to remove the row(s)/column(s) of which the sum is zero. Default is TRUE. |
| `output.wd` | a folder path, where the files will be saved when rtree.save, detail.save, or qp.save is true. |
| `correct.special` | logic, whether to correct the special cases when calculating bNRI or bNTI. Default is TRUE. |
| `unit.sum` | NULL or a number or a nemeric vector. When a beta diversity index is calculated for a bin, the taxa abundances will be divided by unit.sum to calculate the relative abundances, and the Bray-Cuits index in each bin will become manhattan index divided by 2. Default setting are the row sums of community matrix, which are usually sequencing depth in each sample. If set as NULL, means not to do this special transformation. |
| `special.method` | When correct.special is TRUE, which method will be used to check underestimation of deterministic pattern(s) in special cases. MPD, use null model test based on mean pairwise distance; MNTD, use null model test based on mean nearest taxon distance; depend, use MPD when phylo.metric is bMPD or bNRI, and use MNTD when phylo.metric is bMNTD or bNTI; both, use both MPD and MNTD. Default is depend |
| `ses.cut` | numeric, the cutoff of significant standard effect size, default is 1.96. |
| `rc.cut` | numeric, the cutoff of significant modified Raup-Crick index value, default is 0.95. |
| `conf.cut` | numeric, the cutoff of significant one-side confidence level, default is 0.975. |
| `omit.option` | three options about omitting small bins. "no" means to merge small bins to their nearest relatives to meet the bin size requirement, rather than omitting them; "test" means to output the information of small strict bins with a size lower than requirement, iCAMP will not be performed; "omit" means to do iCAMP analysis with strict bins which have enough species (larger than bin size requirement). |
| `meta.ab` | a numeric vector, to define the relative aubndance of each species in the regional pool. Default setting is NULL, means to calculate meta.ab as average relative abundance of each species across the samples. |

### Details

This is the main function of iCAMP (Ning et al 2020). Most parameters can use the default settings.

To quantify various ecological processes, the observed taxa are first divided into different groups ('bins') based on their phylogenetic relationships. Then, the process governing each bin is identified based on null model analysis of the phylogenetic diversity using beta Net Relatedness Index (betaNRI), and taxonomic beta-diversities using modified Raup-Crick metric (RC; a typical setting of sig.index as SES.RC). For each bin, the fraction of pairwise comparisons with betaNRI < -1.96 is considered as the percentages of homogeneous selection, whereas those with betaNRI > +1.96 as the percentages of heterogeneous selection based on the threshold applied previously (Stegen et al 2015; Zhou and Ning 2017). Next, taxonomic diversity metric RC is used to partition the remaining pairwise comparisons with abs(NRI) <= 1.96. The fraction of pairwise comparisons with RC < -0.95 is treated as the percentages of homogenizing dispersal, while those with RC > 0.95 as dispersal limitation (Stegen et al 2013). The remains with abs(NRI) <= 1.96 and abs(RC) <= 0.95 represent the percentages of drift, diversification, weak selection and/or weak dispersal(Zhou and Ning 2017), simply designated as 'drift'(Stegen et al 2013) for convenience. The above analysis is repeated for every bin. Subsequently, the fractions of individual processes across all bins are weighted by the relative abundance of each bin, and summarized to estimate the relative importance of individual processes at the whole community level. Besides betaNRI and RC, null model significance can also be inferred by direct test based on null model distribution, which should be a preferred choice when the null model simulated values do not follow normal distribution (Veech 2012). See the references for details.

Bigmemory (Kane et al 2013) is used to deal with large datasets.

### Value

If omit.option is test, the output will be a table summarizing the information of small bins.

Otherwise, the output is a list object, including one or more elements as below:

The first one or selveral (if set 'both' for metrics and/or randomization scale) elements are matrixes of process importances at community level. In each matrix, the first two columns will be sample ID of each turnover, and the third to last column will show estimated relative importance of each process in shaping each turnover between communities (samples). The name(s) of the element(s) shows the metrics and its randomization scale, e.g. bNRIiRCa means phylogenetic null model analysis using betaNRI (i.e. SES based on betaMPD) with randomizaiton within each bin and taxonomic null model analysis using RC based on Bray-Curtis with randomization across bins. Other possible phylogenetic null-model-based metrics: bNTI, betaNTI (i.e. SES based on betaMNTD); RCbMPD, RC based on betaMPD; RCbMNTD, RC based on betaMNTD; CbMPD, confidence level based on betaMPD; CbMNTD, confidence level based on betaMNTD. Other possible taxonomic null-model-based metrics: SESbray, SES based on Bray-Curtis; CBray, confidence level based on Bray-Curtis. i, within-bin randomization; a, across-bin randomization.

|  |  |
| --- | --- |
| `detail` | an element in output only if detail.save is TRUE. A list with elements as below. |
| `taxabin` | an element in 'detail'. A list, show phylogenetic binning results.  The first element is a matrix named sp.bin, where each row is a taxon (OTU or ASV), the first column is the original strict bin ID, the second column is the original bin ID after small bins are merged into nearest relative(s), the third column is the final renewed bin ID.  The second element named bin.united.sp is a list, where each element shows taxa IDs within each bin and the bins are in the order of the final renewed bin IDs.  The third element named bin.strict.sp is a list, where each element shows taxa IDs within each strict bin and the bins are in the order of the original strict bin IDs.  The fourth element named state.strict is a matrix, where the 1st column is orginal strict bin IDs, the 2nd column is the taxa number in each strict bin, the 3rd to 5th columns show the maximum, mean, and standard deviation of phylogenetic distances within each strict bin.  The fifth element named state.united is a matrix, where the row numbering is the final bin ID, the 1st column is orginal bin IDs, the 2nd column is the taxa number in each final bin, the 3rd to 5th columns show the maximum, mean, and standard deviation of phylogenetic distances within each final bin. |
| `SigbMPDi, SigbMPDa, SigbMNTDi, SigbMNTDa, SigBCi, SigBCa` | elements in 'detail', matrixes showing null model significance testing index for each turnover of each bin. In the name of the element(s), SigbMPD, SigMNTD, or SigBC mean the significance testing is based on betaMPD, betaMNTD, or Bray-Curtis; i, within-bin randomization; a, across-bin randomization. In each matrix, the first two columns are sample IDs for each turnover; the 3rd to the last column represent different bins with column names containing the significance testing index name, which can be bNRI, bNTI, RCbMPD, RCbMNTD, CbMPD, CbMNTD, SESbray, RCbray, or CBray as mentioned above. |
| `bin.weight` | an element in 'detail', a matrix showing relative abundance of each bin in each pair of samples. |
| `processes` | an element in 'detail', a list of process importance results at community level. |
| `setting` | an element in 'detail', a data.frame showing all basic settings of this function. |
| `comm` | an element in 'detail', the input community matrix. |
| `rand` | an element in output only if detail.null is TRUE. It is a list with each element showing the observed or null values of a beta diversity index (e.g. betaMPD, betaMNTD, Bray-Curtis). Each index is showed as a list where each element represents a bin. |
| `special.crct` | an element in output only if detail.null is TRUE. It shows the corrected values for special cases, where zero means no correction is needed. |

### Note

Version 5: 2020.8.19, update help document, add example.
Version 4: 2020.5.31
Version 3: 2019.9.30

### Author(s)

Daliang Ning

### References

Ning, D., Yuan, M., Wu, L., Zhang, Y., Guo, X., Zhou, X. et al. (2020). A quantitative framework reveals the ecological drivers of grassland soil microbial community assembly in response to warming. bioRxiv. doi:10.1101/2020.02.22.960872 (Nature Communications, final revision).

Stegen, J.C., Lin, X., Fredrickson, J.K. & Konopka, A.E. (2015). Estimating and mapping ecological processes influencing microbial community assembly. Front Microbiol, 6, 370.

Stegen, J.C., Lin, X., Fredrickson, J.K., Chen, X., Kennedy, D.W., Murray, C.J. et al. (2013). Quantifying community assembly processes and identifying features that impose them. ISME J, 7, 2069.

Zhou, J. & Ning, D. (2017). Stochastic community assembly: Does it matter in microbial ecology? Microbiology and Molecular Biology Reviews, 81.

Veech, J.A. (2012). Significance testing in ecological null models. Theor Ecol, 5, 611-616.

Kane, M.J., Emerson, J., Weston, S. (2013). Scalable Strategies for Computing with Massive Data. Journal of Statistical Software, 55(14), 1-19. URL http://www.jstatsoft.org/v55/i14/.

### See Also

`qp.bin.js`

### Examples

```
data("example.data")
comm=example.data$comm
tree=example.data$tree
# since need to save some output to a certain folder,
# the following code is set as 'not run'.
# but you may test the code on your computer
# after change the folder path for 'save.wd'.
## Not run: 
  save.wd="C:/test/" # please change to the folder you want to save the pd.big output.
  pd.wd=paste0(save.wd,"/pdbig")
  nworker=4 # parallel computing thread number
  rand.time=20 # usually use 1000 for real data.
  
  bin.size.limit=5 # for real data, usually use a proper number
  # according to phylogenetic signal test or try some settings
  # then choose the reasonable stochasticity level.
  # our experience is 12, or 24, or 48.
  # but for this example dataset which is too small, have to use 5.
  
  setwd(save.wd)
  icamp.out=icamp.big(comm=comm,tree=tree,pd.wd=pd.wd,
                      rand=rand.time, nworker=nworker,
                      bin.size.limit=bin.size.limit)

## End(Not run)
```

---

[Package *iCAMP* version 1.2.4 Index]
