# Supplementary material for: A quantitative framework reveals ecological drivers of grassland microbial community assembly in response to warming
Source: Nat Commun. 2020 Sep 18;11:4717. doi: 10.1038/s41467-020-18560-z (PMC7501310; doi:10.1038/s41467-020-18560-z)
Supplement: Supplementary file 5 — Supplementary Code 1 [file 41467_2020_18560_MOESM5_ESM.zip › iCAMP_1.2.4.zip!/iCAMP/html/icamp.bins.html]

R: Summarize iCAMP result in each bin

|  |  |
| --- | --- |
| icamp.bins {iCAMP} | R Documentation |

## Summarize iCAMP result in each bin

### Description

This function is to calculate various statistic index to assess relative importance of each process in each bin and each turnover, and bin's contribution to each process.

### Usage

```
icamp.bins(icamp.detail, treat = NULL, clas = NULL, silent = FALSE,
          boot = FALSE, rand.time = 1000, between.group = FALSE)
```

### Arguments

|  |  |
| --- | --- |
| `icamp.detail` | list object, the output or the "detail" element of the output from `icamp.big` |
| `treat` | matrix or data.frame, indicating the group or treatment of each sample, rownames are sample IDs. Allow to input multi-column matrix, different columns represent different ways to group the samples. |
| `clas` | matrix or data.frame, the classification information of species (OTUs). |
| `silent` | Logic, whether to show messages. Default is FALSE, thus all messages will be showed. |
| `boot` | Logic, whether to do bootstrapping test to get significance of dominating process in each bin. |
| `rand.time` | integer, bootstrapping times. |
| `between.group` | Logic, whether to analyze between-treatment turnovers. |

### Details

Bin level analysis can provide insights into community assembly mechanisms. This function provides more detailed statistics with the output of the main function `icamp.big`.

### Value

Output is a list object.

|  |  |
| --- | --- |
| `Wtuvk` | The dominant process in each turnover of each bin. |
| `Ptuv` | Relative importance of each process in governing the turnovers between each pair of communities (samples). |
| `Ptk` | Relative importance of each process in governing the turnovers of each bin among a group of samples. |
| `Pt` | Relative importance of each process in governing the turnovers in a group of samples. |
| `BPtk` | Bin contribution to each process, measuring the contribution of each bin to the relative importance of each process in the assembly of a group of communities. |
| `BRPtk` | Bin relative contribution to each process, measuring the relative contribution of each bin to a certain process. |
| `Binwt` | Output if treat is given. Bin relative abundance in each group (treatment) of samples. |
| `Bin.TopClass` | Output if clas is given. A matrix showing the bin relative abundance; the top taxon ID, percentage in bin, and classification; the most abundant name at each phylogeny level in the bin. |
| `Class.Bin` | Output if clas is given. A matrix showing the bin ID and classification information for each taxon. |

### Note

Version 2: 2020.8.19, update help document, add example.
Version 1: 2019.12.11

### Author(s)

Daliang Ning

### References

Ning, D., Yuan, M., Wu, L., Zhang, Y., Guo, X., Zhou, X. et al. (2020). A quantitative framework reveals the ecological drivers of grassland soil microbial community assembly in response to warming. bioRxiv. doi:10.1101/2020.02.22.960872 (Nature Communications, final revision).

### See Also

`icamp.big`

### Examples

```
data("icamp.out")
data("example.data")
treatment=example.data$treat
classification=example.data$classification
rand.time=20 # usually use 1000 for real data.
icampbin=icamp.bins(icamp.detail = icamp.out, treat = treatment,
                    clas = classification, boot = TRUE,
                    rand.time = rand.time, between.group = TRUE)
```

---

[Package *iCAMP* version 1.2.4 Index]
