# Supplementary material for: A quantitative framework reveals ecological drivers of grassland microbial community assembly in response to warming
Source: Nat Commun. 2020 Sep 18;11:4717. doi: 10.1038/s41467-020-18560-z (PMC7501310; doi:10.1038/s41467-020-18560-z)
Supplement: Supplementary file 5 — Supplementary Code 1 [file 41467_2020_18560_MOESM5_ESM.zip › iCAMP_1.2.4.zip!/iCAMP/html/icamp.boot.html]

R: Bootstrapping analysis of icamp results

|  |  |
| --- | --- |
| icamp.boot {iCAMP} | R Documentation |

## Bootstrapping analysis of icamp results

### Description

Use bootstrapping to estimate the variation of relative importance of each process in each group, and compare the difference between groups.

### Usage

```
icamp.boot(icamp.result, treat, rand.time = 1000, compare = TRUE,
           silent = FALSE, between.group = FALSE, ST.estimation = FALSE)
```

### Arguments

|  |  |
| --- | --- |
| `icamp.result` | data.frame object, from the output of `icamp.big`. the first two columns are sample IDs, the third to seventh columns are the relative importance of the five ecological processes. |
| `treat` | matrix or data.frame, a one-column (n x 1) matrix indicating the group or treatment of each sample, rownames are sample IDs. if input a n x m matrix, only the first column is used. |
| `rand.time` | integer, bootstrapping times. default is 1000. |
| `compare` | logic, whether to compare icamp reults between different groups. |
| `silent` | logic, if FALSE, some messages will show during calculation. |
| `between.group` | logic, whether to analyze between-treatment turnovers. |
| `ST.estimation` | logic, whether to estimate stochasticity as the total relative importance of dispersal and drift. |

### Details

Bootstrapping is implemented by random draw samples with replacement, to estimate the variation of relative importance of each process in each group, and calculate the relative difference, effect size, and significance of the difference between each two groups.

### Value

Output is a list with three elements.

|  |  |
| --- | --- |
| `summary` | data.frame, summary of each group.  Group: group name from the input "treat".  Process: process name from the icamp.result.  Observed: the mean relative importance of each process in each group.  Mean, Stdev, Min, Quartile25, Median, Quartile75, and Max: mean, standard deviation, minimum, 25 percent-quantile, median, 75 percent-quantile, and maximum of bootstrapping results, respectively.  Lower.whisker, Lower.hinge, Mediean.1, Higher.hinge, Higher.whisker, Outerlier1...: boxplot elements. |
| `compare` | data.frame, summary of comaprison between each two groups. First two columns are group names. From the third column, different indexes for comparison are showed, including Cohen's d (Cohen.d), effect size magnitude according to Cohen's d (Effect.Size), and P value from bootstrapping test (P.value). |
| `boot.detail` | a list of matrixes, each matrix corresponds to a group, showing detailed bootstrapping results in each random draw. |

### Note

Version 2: 2020.8.19, update help document, add example.
Version 1: 2019.11.14

### Author(s)

Daliang Ning

### References

Ning, D., Yuan, M., Wu, L., Zhang, Y., Guo, X., Zhou, X. et al. (2020). A quantitative framework reveals the ecological drivers of grassland soil microbial community assembly in response to warming. bioRxiv. doi:10.1101/2020.02.22.960872 (Nature Communications, final revision).

### See Also

`icamp.big`

### Examples

```
data("icamp.out")
data("example.data")
treatment=example.data$treat
rand.time=20 # usually use 1000 for real data.
icampbt=icamp.boot(icamp.result = icamp.out$bNRIiRCa, treat = treatment,
                   rand.time = rand.time, compare = TRUE,
                   between.group = TRUE, ST.estimation = TRUE)
```

---

[Package *iCAMP* version 1.2.4 Index]
