# Supplementary material for: A quantitative framework reveals ecological drivers of grassland microbial community assembly in response to warming
Source: Nat Commun. 2020 Sep 18;11:4717. doi: 10.1038/s41467-020-18560-z (PMC7501310; doi:10.1038/s41467-020-18560-z)
Supplement: Supplementary file 5 — Supplementary Code 1 [file 41467_2020_18560_MOESM5_ESM.zip › iCAMP_1.2.4.zip!/iCAMP/html/icamp.out.html]

R: Example output of function icamp.big

|  |  |
| --- | --- |
| icamp.out {iCAMP} | R Documentation |

## Example output of function icamp.big

### Description

a typical output of icamp.big.

### Usage

```
data("icamp.out")
```

### Format

The format is:
List of 4
$ bNRIiRCa :'data.frame': 190 obs. of 7 variables:
..$ sample1 : Factor w/ 19 levels "s10","s11","s12",..: 11 13 14 15 16 17 18 19 1 2 ...
..$ sample2 : Factor w/ 19 levels "s1","s10","s11",..: 1 1 1 1 1 1 1 1 1 1 ...
..$ Heterogeneous.Selection: num [1:190] 0 0 0 0 0 0 0 0 0 0 ...
..$ Homogeneous.Selection : num [1:190] 0 0 0 0 0 ...
..$ Dispersal.Limitation : num [1:190] 0 0 0 0 0 0 0 0 0 0 ...
..$ Homogenizing.Dispersal : num [1:190] 0 0.649 0.628 0 0 ...
..$ Drift.and.Others : num [1:190] 1 0.351 0.372 1 1 ...
$ detail :List of 7
..$ taxabin :List of 5
.. ..$ sp.bin :'data.frame': 30 obs. of 3 variables:
.. .. ..$ bin.id.strict: int [1:30] 1 2 3 4 5 6 7 8 9 10 ...
.. .. ..$ bin.id.united: chr [1:30] "34" "34" "34" "34" ...
.. .. ..$ bin.id.new : num [1:30] 1 1 1 1 1 2 2 2 2 2 ...
.. ..$ bin.united.sp:List of 3
.. .. ..$ : chr [1:5] "OTU1" "OTU2" "OTU3" "OTU4" ...
.. .. ..$ : chr [1:7] "OTU6" "OTU7" "OTU8" "OTU9" ...
.. .. ..$ : chr [1:18] "OTU13" "OTU14" "OTU15" "OTU16" ...
.. ..$ bin.strict.sp:List of 30
.. .. ..$ 1 : chr "OTU1"
.. .. ..$ 2 : chr "OTU2"
.. .. ..$ 3 : chr "OTU3"
.. .. ..$ 4 : chr "OTU4"
.. .. ..$ 5 : chr "OTU5"
.. .. ..$ 6 : chr "OTU6"
.. .. ..$ 7 : chr "OTU7"
.. .. ..$ 8 : chr "OTU8"
.. .. ..$ 9 : chr "OTU9"
.. .. ..$ 10: chr "OTU10"
.. .. ..$ 11: chr "OTU11"
.. .. ..$ 12: chr "OTU12"
.. .. ..$ 13: chr "OTU13"
.. .. ..$ 14: chr "OTU14"
.. .. ..$ 15: chr "OTU15"
.. .. ..$ 16: chr "OTU16"
.. .. ..$ 17: chr "OTU17"
.. .. ..$ 18: chr "OTU18"
.. .. ..$ 19: chr "OTU19"
.. .. ..$ 20: chr "OTU20"
.. .. ..$ 21: chr "OTU21"
.. .. ..$ 22: chr "OTU22"
.. .. ..$ 23: chr "OTU23"
.. .. ..$ 24: chr "OTU24"
.. .. ..$ 25: chr "OTU25"
.. .. ..$ 26: chr "OTU26"
.. .. ..$ 27: chr "OTU27"
.. .. ..$ 28: chr "OTU28"
.. .. ..$ 29: chr "OTU29"
.. .. ..$ 30: chr "OTU30"
.. ..$ state.strict :'data.frame': 30 obs. of 5 variables:
.. .. ..$ bin.strict.id : chr [1:30] "1" "2" "3" "4" ...
.. .. ..$ bin.strict.taxa.num: int [1:30, 1] 1 1 1 1 1 1 1 1 1 1 ...
.. .. .. ..- attr(\*, "dimnames")=List of 2
.. .. .. .. ..$ : chr [1:30] "1" "2" "3" "4" ...
.. .. .. .. ..$ : NULL
.. .. ..$ bin.pd.max : num [1:30] 0 0 0 0 0 0 0 0 0 0 ...
.. .. ..$ bin.pd.mean : num [1:30] 0 0 0 0 0 0 0 0 0 0 ...
.. .. ..$ bin.pd.sd : num [1:30] NA NA NA NA NA NA NA NA NA NA ...
.. ..$ state.united :'data.frame': 3 obs. of 5 variables:
.. .. ..$ bin.united.id.old : chr [1:3] "34" "38" "51"
.. .. ..$ bin.united.tax.num: int [1:3] 5 7 18
.. .. ..$ bin.pd.max : num [1:3] 3.33 3.96 6.36
.. .. ..$ bin.pd.mean : num [1:3] 2.04 2.6 3.5
.. .. ..$ bin.pd.sd : num [1:3] 1.073 0.816 1.235
..$ SigbMPDi :'data.frame': 190 obs. of 5 variables:
.. ..$ name1 : Factor w/ 19 levels "s10","s11","s12",..: 11 13 14 15 16 17 18 19 1 2 ...
.. ..$ name2 : Factor w/ 19 levels "s1","s10","s11",..: 1 1 1 1 1 1 1 1 1 1 ...
.. ..$ bNRIi.bin1: num [1:190] 0.919 0.132 -1.207 1.274 1.274 ...
.. ..$ bNRIi.bin2: num [1:190] -0.572 -0.935 0.311 -0.681 0 ...
.. ..$ bNRIi.bin3: num [1:190] 0.919 0.865 1.034 0.345 0.701 ...
..$ SigBCa :'data.frame': 190 obs. of 5 variables:
.. ..$ name1 : Factor w/ 19 levels "s10","s11","s12",..: 11 13 14 15 16 17 18 19 1 2 ...
.. ..$ name2 : Factor w/ 19 levels "s1","s10","s11",..: 1 1 1 1 1 1 1 1 1 1 ...
.. ..$ RCbraya.bin1: num [1:190] -0.81 0.41 0.14 -0.83 -0.8 -0.96 0.3 -0.12 -0.66 -0.75 ...
.. ..$ RCbraya.bin2: num [1:190] 0.79 0.3 0.83 0.45 0.62 0.64 0.83 -0.49 0.84 0.42 ...
.. ..$ RCbraya.bin3: num [1:190] -0.94 -0.98 -1 -0.42 -0.04 -0.82 -0.87 -0.55 -0.92 -0.12 ...
..$ bin.weight:'data.frame': 190 obs. of 5 variables:
.. ..$ samp1: Factor w/ 19 levels "s10","s11","s12",..: 11 13 14 15 16 17 18 19 1 2 ...
.. ..$ samp2: Factor w/ 19 levels "s1","s10","s11",..: 1 1 1 1 1 1 1 1 1 1 ...
.. ..$ bin1 : num [1:190] 0.0873 0.1653 0.1389 0.0975 0.0799 ...
.. ..$ bin2 : num [1:190] 0.242 0.186 0.233 0.204 0.111 ...
.. ..$ bin3 : num [1:190] 0.671 0.649 0.628 0.698 0.809 ...
..$ processes :List of 1
.. ..$ bNRIiRCa:'data.frame': 190 obs. of 7 variables:
.. .. ..$ sample1 : Factor w/ 19 levels "s10","s11","s12",..: 11 13 14 15 16 17 18 19 1 2 ...
.. .. ..$ sample2 : Factor w/ 19 levels "s1","s10","s11",..: 1 1 1 1 1 1 1 1 1 1 ...
.. .. ..$ Heterogeneous.Selection: num [1:190] 0 0 0 0 0 0 0 0 0 0 ...
.. .. ..$ Homogeneous.Selection : num [1:190] 0 0 0 0 0 ...
.. .. ..$ Dispersal.Limitation : num [1:190] 0 0 0 0 0 0 0 0 0 0 ...
.. .. ..$ Homogenizing.Dispersal : num [1:190] 0 0.649 0.628 0 0 ...
.. .. ..$ Drift.and.Others : num [1:190] 1 0.351 0.372 1 1 ...
..$ setting :'data.frame': 1 obs. of 24 variables:
.. ..$ ds : num 0.2
.. ..$ pd.cut : logi NA
.. ..$ max.pd : num 7.83
.. ..$ sp.check : logi TRUE
.. ..$ phylo.rand.scale: Factor w/ 1 level "within.bin": 1
.. ..$ taxa.rand.scale : Factor w/ 1 level "across.all": 1
.. ..$ phylo.metric : Factor w/ 1 level "bMPD": 1
.. ..$ sig.index : Factor w/ 1 level "SES.RC": 1
.. ..$ bin.size.limit : num 5
.. ..$ nworker : num 4
.. ..$ memory.G : num 50
.. ..$ rtree.save : logi FALSE
.. ..$ detail.save : logi TRUE
.. ..$ qp.save : logi FALSE
.. ..$ detail.null : logi TRUE
.. ..$ ignore.zero : logi TRUE
.. ..$ output.wd : Factor w/ 1 level "E:/Dropbox/ToolDevelop/package/iCAMP/LatestVersion/Example/TestOutputs14": 1
.. ..$ correct.special : logi TRUE
.. ..$ unit.sum.mean : num 34.2
.. ..$ special.method : Factor w/ 1 level "depend": 1
.. ..$ ses.cut : num 1.96
.. ..$ rc.cut : num 0.95
.. ..$ conf.cut : num 0.975
.. ..$ omit.option : Factor w/ 1 level "no": 1
..$ comm : int [1:20, 1:30] 1 3 0 0 2 2 2 0 0 4 ...
.. ..- attr(\*, "dimnames")=List of 2
.. .. ..$ : chr [1:20] "s1" "s2" "s3" "s4" ...
.. .. ..$ : chr [1:30] "OTU1" "OTU2" "OTU3" "OTU4" ...
$ rand :List of 4
..$ bMPD.obs :List of 3
.. ..$ : num [1:20, 1:20] 0 0.00798 0.03268 0.01343 0.01722 ...
.. .. ..- attr(\*, "dimnames")=List of 2
.. .. .. ..$ : chr [1:20] "s1" "s2" "s3" "s4" ...
.. .. .. ..$ : chr [1:20] "s1" "s2" "s3" "s4" ...
.. ..$ : num [1:20, 1:20] 0 0.086 0.0216 0.0949 0.047 ...
.. .. ..- attr(\*, "dimnames")=List of 2
.. .. .. ..$ : chr [1:20] "s1" "s2" "s3" "s4" ...
.. .. .. ..$ : chr [1:20] "s1" "s2" "s3" "s4" ...
.. ..$ : num [1:20, 1:20] 0 1.48 1.29 1.27 1.64 ...
.. .. ..- attr(\*, "dimnames")=List of 2
.. .. .. ..$ : chr [1:20] "s1" "s2" "s3" "s4" ...
.. .. .. ..$ : chr [1:20] "s1" "s2" "s3" "s4" ...
..$ bMPDi.rand:List of 3
.. ..$ :'data.frame': 190 obs. of 102 variables:
.. .. ..$ name1 : Factor w/ 19 levels "s10","s11","s12",..: 11 13 14 15 16 17 18 19 1 2 ...
.. .. ..$ name2 : Factor w/ 19 levels "s1","s10","s11",..: 1 1 1 1 1 1 1 1 1 1 ...
.. .. ..$ rand1 : num [1:190] 0.00798 0.03197 0.01217 0.01476 0.00992 ...
.. .. ..$ rand2 : num [1:190] 0.00543 0.02743 0.01838 0.01224 0.00822 ...
.. .. ..$ rand3 : num [1:190] 0.00357 0.0245 0.02358 0.01224 0.00822 ...
.. .. ..$ rand4 : num [1:190] 0.00839 0.04672 0.03604 0.00615 0.00413 ...
.. .. ..$ rand5 : num [1:190] 0.0016 0.0226 0.0313 0.0172 0.0116 ...
.. .. ..$ rand6 : num [1:190] 0.00626 0.03441 0.0261 0.00615 0.00413 ...
.. .. ..$ rand7 : num [1:190] 0.00839 0.03746 0.01959 0.01476 0.00992 ...
.. .. ..$ rand8 : num [1:190] 0.00839 0.03351 0.01257 0.01722 0.01157 ...
.. .. ..$ rand9 : num [1:190] 0.00798 0.03268 0.01343 0.00697 0.00468 ...
.. .. ..$ rand10 : num [1:190] 0.00357 0.02626 0.02671 0.00615 0.00413 ...
.. .. ..$ rand11 : num [1:190] 0.00357 0.03161 0.03623 0.00313 0.0021 ...
.. .. ..$ rand12 : num [1:190] 0.0016 0.0125 0.01325 0.01476 0.00992 ...
.. .. ..$ rand13 : num [1:190] 0.00881 0.05001 0.03956 0.00248 0.00166 ...
.. .. ..$ rand14 : num [1:190] 0.00127 0.02228 0.03252 0.01722 0.01157 ...
.. .. ..$ rand15 : num [1:190] 0.00881 0.04554 0.03161 0.00248 0.00166 ...
.. .. ..$ rand16 : num [1:190] 0.0016 0.02264 0.03127 0.00697 0.00468 ...
.. .. ..$ rand17 : num [1:190] 0.00881 0.03988 0.02154 0.00313 0.0021 ...
.. .. ..$ rand18 : num [1:190] 0.0016 0.02176 0.02971 0.00615 0.00413 ...
.. .. ..$ rand19 : num [1:190] 0.0016 0.0134 0.0148 0.0172 0.0116 ...
.. .. ..$ rand20 : num [1:190] 0.00127 0.02053 0.02939 0.01558 0.01047 ...
.. .. ..$ rand21 : num [1:190] 0.00543 0.03581 0.03327 0.00248 0.00166 ...
.. .. ..$ rand22 : num [1:190] 0.00798 0.04518 0.03565 0.0106 0.00712 ...
.. .. ..$ rand23 : num [1:190] 0.00798 0.03197 0.01217 0.0106 0.00712 ...
.. .. ..$ rand24 : num [1:190] 0.00881 0.05001 0.03956 0.01224 0.00822 ...
.. .. ..$ rand25 : num [1:190] 0.00543 0.03309 0.02845 0.00697 0.00468 ...
.. .. ..$ rand26 : num [1:190] 0.00839 0.03421 0.01382 0.00615 0.00413 ...
.. .. ..$ rand27 : num [1:190] 0.00357 0.02626 0.02671 0.0106 0.00712 ...
.. .. ..$ rand28 : num [1:190] 0.00881 0.03575 0.01421 0.00697 0.00468 ...
.. .. ..$ rand29 : num [1:190] 0.00626 0.03441 0.0261 0.00697 0.00468 ...
.. .. ..$ rand30 : num [1:190] 0.00798 0.04782 0.04035 0.00697 0.00468 ...
.. .. ..$ rand31 : num [1:190] 0.00626 0.04064 0.03718 0.00248 0.00166 ...
.. .. ..$ rand32 : num [1:190] 0.00127 0.02316 0.03408 0.01224 0.00822 ...
.. .. ..$ rand33 : num [1:190] 0.00626 0.04152 0.03875 0.00248 0.00166 ...
.. .. ..$ rand34 : num [1:190] 0.00881 0.03575 0.01421 0.01558 0.01047 ...
.. .. ..$ rand35 : num [1:190] 0.0016 0.0244 0.0344 0.0156 0.0105 ...
.. .. ..$ rand36 : num [1:190] 0.00543 0.02655 0.01681 0.01224 0.00822 ...
.. .. ..$ rand37 : num [1:190] 0.00127 0.01781 0.02457 0.0164 0.01102 ...
.. .. ..$ rand38 : num [1:190] 0.00756 0.03916 0.02731 0.00248 0.00166 ...
.. .. ..$ rand39 : num [1:190] 0.00756 0.03438 0.0188 0.00313 0.0021 ...
.. .. ..$ rand40 : num [1:190] 0.00357 0.01647 0.00931 0.01722 0.01157 ...
.. .. ..$ rand41 : num [1:190] 0.00127 0.02053 0.02939 0.0106 0.00712 ...
.. .. ..$ rand42 : num [1:190] 0.0016 0.0125 0.0132 0.0164 0.011 ...
.. .. ..$ rand43 : num [1:190] 0.0016 0.0218 0.0297 0.0164 0.011 ...
.. .. ..$ rand44 : num [1:190] 0.00756 0.0454 0.03839 0.00313 0.0021 ...
.. .. ..$ rand45 : num [1:190] 0.0016 0.02352 0.03283 0.00615 0.00413 ...
.. .. ..$ rand46 : num [1:190] 0.00626 0.02963 0.0176 0.00697 0.00468 ...
.. .. ..$ rand47 : num [1:190] 0.00315 0.02472 0.02632 0.0106 0.00712 ...
.. .. ..$ rand48 : num [1:190] 0.00798 0.04782 0.04035 0.00313 0.0021 ...
.. .. ..$ rand49 : num [1:190] 0.00315 0.02744 0.03115 0.0164 0.01102 ...
.. .. ..$ rand50 : num [1:190] 0.00839 0.03746 0.01959 0.00313 0.0021 ...
.. .. ..$ rand51 : num [1:190] 0.00127 0.02141 0.03096 0.0106 0.00712 ...
.. .. ..$ rand52 : num [1:190] 0.00839 0.03421 0.01382 0.01476 0.00992 ...
.. .. ..$ rand53 : num [1:190] 0.00839 0.04935 0.04074 0.01224 0.00822 ...
.. .. ..$ rand54 : num [1:190] 0.00315 0.02919 0.03427 0.00313 0.0021 ...
.. .. ..$ rand55 : num [1:190] 0.00357 0.02986 0.0331 0.00313 0.0021 ...
.. .. ..$ rand56 : num [1:190] 0.00357 0.01647 0.00931 0.01558 0.01047 ...
.. .. ..$ rand57 : num [1:190] 0.00315 0.02296 0.02319 0.00697 0.00468 ...
.. .. ..$ rand58 : num [1:190] 0.00756 0.0454 0.03839 0.00615 0.00413 ...
.. .. ..$ rand59 : num [1:190] 0.00315 0.01493 0.00892 0.0164 0.01102 ...
.. .. ..$ rand60 : num [1:190] 0.00315 0.02744 0.03115 0.00313 0.0021 ...
.. .. ..$ rand61 : num [1:190] 0.00756 0.03438 0.0188 0.0164 0.01102 ...
.. .. ..$ rand62 : num [1:190] 0.00839 0.044 0.03122 0.00248 0.00166 ...
.. .. ..$ rand63 : num [1:190] 0.00798 0.0368 0.02076 0.00313 0.0021 ...
.. .. ..$ rand64 : num [1:190] 0.00315 0.01493 0.00892 0.01476 0.00992 ...
.. .. ..$ rand65 : num [1:190] 0.00798 0.04518 0.03565 0.00248 0.00166 ...
.. .. ..$ rand66 : num [1:190] 0.00626 0.02963 0.0176 0.0106 0.00712 ...
.. .. ..$ rand67 : num [1:190] 0.00881 0.03505 0.01296 0.0164 0.01102 ...
.. .. ..$ rand68 : num [1:190] 0.00626 0.04152 0.03875 0.0164 0.01102 ...
.. .. ..$ rand69 : num [1:190] 0.00626 0.02568 0.01058 0.0164 0.01102 ...
.. .. ..$ rand70 : num [1:190] 0.00127 0.02141 0.03096 0.01476 0.00992 ...
.. .. ..$ rand71 : num [1:190] 0.00798 0.0407 0.0277 0.01476 0.00992 ...
.. .. ..$ rand72 : num [1:190] 0.00839 0.04935 0.04074 0.00248 0.00166 ...
.. .. ..$ rand73 : num [1:190] 0.00543 0.02743 0.01838 0.00615 0.00413 ...
.. .. ..$ rand74 : num [1:190] 0.00881 0.03505 0.01296 0.01224 0.00822 ...
.. .. ..$ rand75 : num [1:190] 0.00626 0.04064 0.03718 0.01722 0.01157 ...
.. .. ..$ rand76 : num [1:190] 0.00881 0.03988 0.02154 0.01558 0.01047 ...
.. .. ..$ rand77 : num [1:190] 0.00315 0.01906 0.01625 0.01224 0.00822 ...
.. .. ..$ rand78 : num [1:190] 0.00756 0.03114 0.01304 0.00615 0.00413 ...
.. .. ..$ rand79 : num [1:190] 0.0016 0.02352 0.03283 0.01476 0.00992 ...
.. .. ..$ rand80 : num [1:190] 0.00127 0.01605 0.02144 0.01476 0.00992 ...
.. .. ..$ rand81 : num [1:190] 0.00127 0.01605 0.02144 0.01558 0.01047 ...
.. .. ..$ rand82 : num [1:190] 0.00543 0.03669 0.03484 0.01476 0.00992 ...
.. .. ..$ rand83 : num [1:190] 0.00626 0.03051 0.01916 0.0106 0.00712 ...
.. .. ..$ rand84 : num [1:190] 0.00798 0.0368 0.02076 0.01722 0.01157 ...
.. .. ..$ rand85 : num [1:190] 0.00881 0.04914 0.038 0.00313 0.0021 ...
.. .. ..$ rand86 : num [1:190] 0.00357 0.02986 0.0331 0.01722 0.01157 ...
.. .. ..$ rand87 : num [1:190] 0.00756 0.03043 0.01178 0.0106 0.00712 ...
.. .. ..$ rand88 : num [1:190] 0.00756 0.03114 0.01304 0.0164 0.01102 ...
.. .. ..$ rand89 : num [1:190] 0.00543 0.0226 0.00979 0.01558 0.01047 ...
.. .. ..$ rand90 : num [1:190] 0.00626 0.02568 0.01058 0.01722 0.01157 ...
.. .. ..$ rand91 : num [1:190] 0.00127 0.02316 0.03408 0.0164 0.01102 ...
.. .. ..$ rand92 : num [1:190] 0.00756 0.03916 0.02731 0.01558 0.01047 ...
.. .. ..$ rand93 : num [1:190] 0.00881 0.04914 0.038 0.00697 0.00468 ...
.. .. ..$ rand94 : num [1:190] 0.00756 0.04452 0.03683 0.00248 0.00166 ...
.. .. ..$ rand95 : num [1:190] 0.0016 0.0134 0.0148 0.0156 0.0105 ...
.. .. ..$ rand96 : num [1:190] 0.00357 0.01972 0.01508 0.0106 0.00712 ...
.. .. ..$ rand97 : num [1:190] 0.00543 0.03309 0.02845 0.00615 0.00413 ...
.. .. .. [list output truncated]
.. ..$ :'data.frame': 190 obs. of 102 variables:
.. .. ..$ name1 : Factor w/ 19 levels "s10","s11","s12",..: 11 13 14 15 16 17 18 19 1 2 ...
.. .. ..$ name2 : Factor w/ 19 levels "s1","s10","s11",..: 1 1 1 1 1 1 1 1 1 1 ...
.. .. ..$ rand1 : num [1:190] 0.1008 0.0194 0.1046 0.0538 0 ...
.. .. ..$ rand2 : num [1:190] 0.1009 0.0418 0.0792 0.059 0 ...
.. .. ..$ rand3 : num [1:190] 0.093 0.0289 0.0847 0.0521 0 ...
.. .. ..$ rand4 : num [1:190] 0.0695 0.0198 0.0566 0.0386 0 ...
.. .. ..$ rand5 : num [1:190] 0.1207 0.0288 0.1134 0.0656 0 ...
.. .. ..$ rand6 : num [1:190] 0.0985 0.0328 0.0897 0.0557 0 ...
.. .. ..$ rand7 : num [1:190] 0.0769 0.0274 0.0749 0.0439 0 ...
.. .. ..$ rand8 : num [1:190] 0.0659 0.0216 0.0713 0.0372 0 ...
.. .. ..$ rand9 : num [1:190] 0.1118 0.0418 0.1078 0.0643 0 ...
.. .. ..$ rand10 : num [1:190] 0.1054 0.0256 0.0966 0.0574 0 ...
.. .. ..$ rand11 : num [1:190] 0.0823 0.0288 0.0721 0.0469 0 ...
.. .. ..$ rand12 : num [1:190] 0.1124 0.0274 0.0929 0.0613 0 ...
.. .. ..$ rand13 : num [1:190] 0.1193 0.044 0.1063 0.0685 0 ...
.. .. ..$ rand14 : num [1:190] 0.1036 0.0384 0.094 0.0595 0 ...
.. .. ..$ rand15 : num [1:190] 0.0938 0.0271 0.0728 0.0521 0 ...
.. .. ..$ rand16 : num [1:190] 0.1036 0.0384 0.0924 0.0595 0 ...
.. .. ..$ rand17 : num [1:190] 0.1112 0.0385 0.0964 0.0633 0 ...
.. .. ..$ rand18 : num [1:190] 0.0881 0.0328 0.0837 0.0506 0 ...
.. .. ..$ rand19 : num [1:190] 0.0876 0.0271 0.0684 0.0491 0 ...
.. .. ..$ rand20 : num [1:190] 0.0846 0.0198 0.0862 0.0459 0 ...
.. .. ..$ rand21 : num [1:190] 0.1051 0.0148 0.101 0.0548 0 ...
.. .. ..$ rand22 : num [1:190] 0.1387 0.0384 0.1101 0.0766 0 ...
.. .. ..$ rand23 : num [1:190] 0.08 0.0295 0.0768 0.0459 0 ...
.. .. ..$ rand24 : num [1:190] 0.1174 0.0311 0.1051 0.0646 0 ...
.. .. ..$ rand25 : num [1:190] 0.0883 0.0256 0.0691 0.0491 0 ...
.. .. ..$ rand26 : num [1:190] 0.1071 0.0385 0.088 0.0613 0 ...
.. .. ..$ rand27 : num [1:190] 0.1282 0.0311 0.1127 0.0699 0 ...
.. .. ..$ rand28 : num [1:190] 0.1254 0.044 0.12 0.0715 0 ...
.. .. ..$ rand29 : num [1:190] 0.1009 0.0418 0.0917 0.059 0 ...
.. .. ..$ rand30 : num [1:190] 0.1054 0.0194 0.0979 0.056 0 ...
.. .. ..$ rand31 : num [1:190] 0.099 0.0328 0.0919 0.056 0 ...
.. .. ..$ rand32 : num [1:190] 0.0938 0.0271 0.0728 0.0521 0 ...
.. .. ..$ rand33 : num [1:190] 0.1008 0.0311 0.1012 0.0565 0 ...
.. .. ..$ rand34 : num [1:190] 0.1147 0.0418 0.0949 0.0657 0 ...
.. .. ..$ rand35 : num [1:190] 0.0825 0.0289 0.0677 0.047 0 ...
.. .. ..$ rand36 : num [1:190] 0.1031 0.0328 0.0926 0.058 0 ...
.. .. ..$ rand37 : num [1:190] 0.0899 0.0385 0.0949 0.0529 0 ...
.. .. ..$ rand38 : num [1:190] 0.1104 0.0216 0.1006 0.059 0 ...
.. .. ..$ rand39 : num [1:190] 0.0869 0.0194 0.0928 0.047 0 ...
.. .. ..$ rand40 : num [1:190] 0.0501 0.0198 0.0605 0.0291 0 ...
.. .. ..$ rand41 : num [1:190] 0.094 0.0296 0.0917 0.0528 0 ...
.. .. ..$ rand42 : num [1:190] 0.0865 0.0407 0.0823 0.0517 0 ...
.. .. ..$ rand43 : num [1:190] 0.1221 0.044 0.1069 0.0699 0 ...
.. .. ..$ rand44 : num [1:190] 0.0541 0.0114 0.0642 0.0291 0 ...
.. .. ..$ rand45 : num [1:190] 0.0805 0.0198 0.0782 0.0439 0 ...
.. .. ..$ rand46 : num [1:190] 0.0934 0.0216 0.1001 0.0506 0 ...
.. .. ..$ rand47 : num [1:190] 0.094 0.0274 0.0969 0.0522 0 ...
.. .. ..$ rand48 : num [1:190] 0.1015 0.0296 0.085 0.0565 0 ...
.. .. ..$ rand49 : num [1:190] 0.1015 0.0296 0.1019 0.0565 0 ...
.. .. ..$ rand50 : num [1:190] 0.0908 0.0198 0.0857 0.049 0 ...
.. .. ..$ rand51 : num [1:190] 0.0879 0.0418 0.0761 0.0527 0 ...
.. .. ..$ rand52 : num [1:190] 0.1118 0.0271 0.0854 0.0609 0 ...
.. .. ..$ rand53 : num [1:190] 0.1032 0.0361 0.0784 0.0588 0 ...
.. .. ..$ rand54 : num [1:190] 0.0794 0.0296 0.0885 0.0456 0 ...
.. .. ..$ rand55 : num [1:190] 0.1112 0.0384 0.0909 0.0632 0 ...
.. .. ..$ rand56 : num [1:190] 0.133 0.0418 0.114 0.0746 0 ...
.. .. ..$ rand57 : num [1:190] 0.0988 0.0148 0.0938 0.0517 0 ...
.. .. ..$ rand58 : num [1:190] 0.1199 0.0296 0.1148 0.0655 0 ...
.. .. ..$ rand59 : num [1:190] 0.1435 0.0418 0.1215 0.0798 0 ...
.. .. ..$ rand60 : num [1:190] 0.1249 0.0361 0.1007 0.0694 0 ...
.. .. ..$ rand61 : num [1:190] 0.0917 0.0256 0.0855 0.0508 0 ...
.. .. ..$ rand62 : num [1:190] 0.1032 0.0361 0.094 0.0588 0 ...
.. .. ..$ rand63 : num [1:190] 0.1252 0.0289 0.1136 0.0679 0 ...
.. .. ..$ rand64 : num [1:190] 0.0965 0.0194 0.1025 0.0517 0 ...
.. .. ..$ rand65 : num [1:190] 0.0766 0.0271 0.0768 0.0437 0 ...
.. .. ..$ rand66 : num [1:190] 0.0995 0.0184 0.1038 0.0529 0 ...
.. .. ..$ rand67 : num [1:190] 0.1209 0.0289 0.1205 0.0657 0 ...
.. .. ..$ rand68 : num [1:190] 0.1118 0.0418 0.1078 0.0643 0 ...
.. .. ..$ rand69 : num [1:190] 0.068 0.0114 0.0691 0.0358 0 ...
.. .. ..$ rand70 : num [1:190] 0.0674 0.0184 0.0708 0.0372 0 ...
.. .. ..$ rand71 : num [1:190] 0.1124 0.0274 0.1128 0.0613 0 ...
.. .. ..$ rand72 : num [1:190] 0.0944 0.0296 0.092 0.053 0 ...
.. .. ..$ rand73 : num [1:190] 0.1237 0.0385 0.1194 0.0694 0 ...
.. .. ..$ rand74 : num [1:190] 0.0801 0.0296 0.0771 0.046 0 ...
.. .. ..$ rand75 : num [1:190] 0.0944 0.0296 0.0805 0.053 0 ...
.. .. ..$ rand76 : num [1:190] 0.1032 0.0361 0.1024 0.0588 0 ...
.. .. ..$ rand77 : num [1:190] 0.1009 0.0418 0.0917 0.059 0 ...
.. .. ..$ rand78 : num [1:190] 0.0733 0.0361 0.0701 0.0442 0 ...
.. .. ..$ rand79 : num [1:190] 0.1051 0.0148 0.0911 0.0548 0 ...
.. .. ..$ rand80 : num [1:190] 0.0822 0.0311 0.0797 0.0474 0 ...
.. .. ..$ rand81 : num [1:190] 0.0986 0.0295 0.0834 0.055 0 ...
.. .. ..$ rand82 : num [1:190] 0.0865 0.0407 0.0823 0.0517 0 ...
.. .. ..$ rand83 : num [1:190] 0.0659 0.0216 0.0713 0.0372 0 ...
.. .. ..$ rand84 : num [1:190] 0.1038 0.0385 0.0946 0.0596 0 ...
.. .. ..$ rand85 : num [1:190] 0.0999 0.044 0.0991 0.059 0 ...
.. .. ..$ rand86 : num [1:190] 0.1174 0.0407 0.0949 0.0668 0 ...
.. .. ..$ rand87 : num [1:190] 0.1326 0.0385 0.1113 0.0737 0 ...
.. .. ..$ rand88 : num [1:190] 0.0846 0.0198 0.0811 0.0459 0 ...
.. .. ..$ rand89 : num [1:190] 0.0769 0.0274 0.0749 0.0439 0 ...
.. .. ..$ rand90 : num [1:190] 0.0755 0.0114 0.0772 0.0395 0 ...
.. .. ..$ rand91 : num [1:190] 0.136 0.044 0.1166 0.0766 0 ...
.. .. ..$ rand92 : num [1:190] 0.1036 0.0384 0.0924 0.0595 0 ...
.. .. ..$ rand93 : num [1:190] 0.0501 0.0198 0.0491 0.0291 0 ...
.. .. ..$ rand94 : num [1:190] 0.0562 0.0148 0.0739 0.0309 0 ...
.. .. ..$ rand95 : num [1:190] 0.0902 0.0289 0.084 0.0508 0 ...
.. .. ..$ rand96 : num [1:190] 0.086 0.0216 0.0869 0.047 0 ...
.. .. ..$ rand97 : num [1:190] 0.093 0.0289 0.0847 0.0521 0 ...
.. .. .. [list output truncated]
.. ..$ :'data.frame': 190 obs. of 102 variables:
.. .. ..$ name1 : Factor w/ 19 levels "s10","s11","s12",..: 11 13 14 15 16 17 18 19 1 2 ...
.. .. ..$ name2 : Factor w/ 19 levels "s1","s10","s11",..: 1 1 1 1 1 1 1 1 1 1 ...
.. .. ..$ rand1 : num [1:190] 1.36 1.25 1.23 1.66 2.19 ...
.. .. ..$ rand2 : num [1:190] 1.46 1.25 1.27 1.79 2.31 ...
.. .. ..$ rand3 : num [1:190] 1.34 1.15 1.06 1.52 1.96 ...
.. .. ..$ rand4 : num [1:190] 1.26 1.13 1.03 1.43 1.94 ...
.. .. ..$ rand5 : num [1:190] 1.349 1.137 0.997 1.545 1.841 ...
.. .. ..$ rand6 : num [1:190] 1.43 1.27 1.23 1.67 2.1 ...
.. .. ..$ rand7 : num [1:190] 1.12 1.02 1.04 1.48 2.07 ...
.. .. ..$ rand8 : num [1:190] 1.38 1.17 1.17 1.8 2.34 ...
.. .. ..$ rand9 : num [1:190] 1.15 0.934 0.867 1.5 1.997 ...
.. .. ..$ rand10 : num [1:190] 1.48 1.26 1.22 1.75 2.26 ...
.. .. ..$ rand11 : num [1:190] 1.39 1.25 1.22 1.48 1.98 ...
.. .. ..$ rand12 : num [1:190] 1.28 1.11 1.17 1.87 2.41 ...
.. .. ..$ rand13 : num [1:190] 1.328 1.109 0.973 1.653 2.036 ...
.. .. ..$ rand14 : num [1:190] 1.5 1.3 1.28 1.62 2.06 ...
.. .. ..$ rand15 : num [1:190] 1.35 1.24 1.27 1.74 2.27 ...
.. .. ..$ rand16 : num [1:190] 0.99 0.865 0.923 1.242 1.662 ...
.. .. ..$ rand17 : num [1:190] 1.26 1.15 1.08 1.52 1.98 ...
.. .. ..$ rand18 : num [1:190] 1.259 1.08 0.976 1.326 1.75 ...
.. .. ..$ rand19 : num [1:190] 1.72 1.53 1.44 2.03 2.62 ...
.. .. ..$ rand20 : num [1:190] 1.085 0.868 0.893 1.23 1.578 ...
.. .. ..$ rand21 : num [1:190] 0.969 0.938 0.883 1.278 1.695 ...
.. .. ..$ rand22 : num [1:190] 1.17 1.03 1 1.4 1.81 ...
.. .. ..$ rand23 : num [1:190] 1.26 1.04 1.06 1.36 1.72 ...
.. .. ..$ rand24 : num [1:190] 1.144 0.993 0.991 1.479 1.978 ...
.. .. ..$ rand25 : num [1:190] 0.973 0.818 0.878 1.294 1.771 ...
.. .. ..$ rand26 : num [1:190] 1.44 1.29 1.25 1.82 2.33 ...
.. .. ..$ rand27 : num [1:190] 1.34 1.23 1.22 1.55 1.9 ...
.. .. ..$ rand28 : num [1:190] 1.21 1.12 1.11 1.6 2.06 ...
.. .. ..$ rand29 : num [1:190] 0.996 0.869 0.977 1.239 1.724 ...
.. .. ..$ rand30 : num [1:190] 1.29 1.04 1.03 1.59 2 ...
.. .. ..$ rand31 : num [1:190] 0.991 0.807 0.907 1.473 1.771 ...
.. .. ..$ rand32 : num [1:190] 1.59 1.3 1.27 1.7 2.23 ...
.. .. ..$ rand33 : num [1:190] 1.52 1.44 1.3 1.92 2.46 ...
.. .. ..$ rand34 : num [1:190] 1.12 1.01 1 1.41 1.77 ...
.. .. ..$ rand35 : num [1:190] 1.76 1.55 1.51 1.88 2.43 ...
.. .. ..$ rand36 : num [1:190] 1.13 1.02 1.08 1.43 1.92 ...
.. .. ..$ rand37 : num [1:190] 1.57 1.25 1.39 1.91 2.4 ...
.. .. ..$ rand38 : num [1:190] 1.4 1.24 1.24 1.66 2.08 ...
.. .. ..$ rand39 : num [1:190] 1.43 1.18 1.13 1.66 2.15 ...
.. .. ..$ rand40 : num [1:190] 1.78 1.5 1.38 1.84 2.31 ...
.. .. ..$ rand41 : num [1:190] 1.33 1.09 1.06 1.55 1.94 ...
.. .. ..$ rand42 : num [1:190] 1.45 1.33 1.22 1.76 2.31 ...
.. .. ..$ rand43 : num [1:190] 1.119 1.014 0.993 1.496 1.879 ...
.. .. ..$ rand44 : num [1:190] 1.4 1.29 1.18 1.63 2.17 ...
.. .. ..$ rand45 : num [1:190] 1.59 1.45 1.29 1.68 2.2 ...
.. .. ..$ rand46 : num [1:190] 1.45 1.28 1.27 1.77 2.22 ...
.. .. ..$ rand47 : num [1:190] 1.24 1.05 1.09 1.5 2.03 ...
.. .. ..$ rand48 : num [1:190] 1.14 1.16 1.17 1.54 2.1 ...
.. .. ..$ rand49 : num [1:190] 0.951 0.928 0.924 1.384 1.856 ...
.. .. ..$ rand50 : num [1:190] 1.39 1.24 1.23 1.65 2.05 ...
.. .. ..$ rand51 : num [1:190] 1.233 1.037 0.956 1.522 1.937 ...
.. .. ..$ rand52 : num [1:190] 0.962 0.815 0.929 1.202 1.568 ...
.. .. ..$ rand53 : num [1:190] 1.42 1.3 1.28 1.95 2.46 ...
.. .. ..$ rand54 : num [1:190] 0.936 0.733 0.769 1.24 1.453 ...
.. .. ..$ rand55 : num [1:190] 1.197 0.968 0.983 1.272 1.743 ...
.. .. ..$ rand56 : num [1:190] 1.48 1.37 1.29 1.7 2.33 ...
.. .. ..$ rand57 : num [1:190] 1.038 0.976 1.023 1.476 1.916 ...
.. .. ..$ rand58 : num [1:190] 1.42 1.3 1.2 1.69 2.18 ...
.. .. ..$ rand59 : num [1:190] 0.979 0.93 0.833 1.418 1.849 ...
.. .. ..$ rand60 : num [1:190] 1.33 1.11 1.15 1.63 2.09 ...
.. .. ..$ rand61 : num [1:190] 1.54 1.23 1.13 1.59 2.07 ...
.. .. ..$ rand62 : num [1:190] 1.117 0.805 0.844 1.329 1.576 ...
.. .. ..$ rand63 : num [1:190] 1.25 1.12 1.21 1.44 1.82 ...
.. .. ..$ rand64 : num [1:190] 1.36 1.18 1.13 1.6 2.09 ...
.. .. ..$ rand65 : num [1:190] 1.15 1.029 0.947 1.348 1.851 ...
.. .. ..$ rand66 : num [1:190] 1.143 0.963 0.956 1.278 1.784 ...
.. .. ..$ rand67 : num [1:190] 1.128 0.834 0.855 1.401 1.661 ...
.. .. ..$ rand68 : num [1:190] 1.02 0.93 0.87 1.57 1.97 ...
.. .. ..$ rand69 : num [1:190] 0.984 0.931 0.919 1.472 1.913 ...
.. .. ..$ rand70 : num [1:190] 1.092 0.835 0.889 1.337 1.635 ...
.. .. ..$ rand71 : num [1:190] 1.2 1.104 0.992 1.777 2.262 ...
.. .. ..$ rand72 : num [1:190] 1.21 1.06 1.14 1.27 1.64 ...
.. .. ..$ rand73 : num [1:190] 1.32 1.07 0.93 1.55 2.07 ...
.. .. ..$ rand74 : num [1:190] 1.5 1.3 1.26 1.74 2.27 ...
.. .. ..$ rand75 : num [1:190] 1.62 1.46 1.42 1.86 2.45 ...
.. .. ..$ rand76 : num [1:190] 1.28 1.09 1.13 1.7 2.2 ...
.. .. ..$ rand77 : num [1:190] 1.156 0.874 0.936 1.548 2.069 ...
.. .. ..$ rand78 : num [1:190] 1.201 0.989 0.922 1.352 1.769 ...
.. .. ..$ rand79 : num [1:190] 1.34 1.12 1.14 1.37 1.8 ...
.. .. ..$ rand80 : num [1:190] 1.31 1.12 1.02 1.59 2.06 ...
.. .. ..$ rand81 : num [1:190] 1.43 1.13 1.03 1.49 1.94 ...
.. .. ..$ rand82 : num [1:190] 1.216 0.798 0.897 1.465 1.809 ...
.. .. ..$ rand83 : num [1:190] 1.139 1.048 0.957 1.462 1.843 ...
.. .. ..$ rand84 : num [1:190] 1.5 1.23 1.18 1.76 2.26 ...
.. .. ..$ rand85 : num [1:190] 1.58 1.46 1.45 1.87 2.53 ...
.. .. ..$ rand86 : num [1:190] 1.39 1.22 1.18 1.65 2.14 ...
.. .. ..$ rand87 : num [1:190] 0.994 0.731 0.766 1.279 1.735 ...
.. .. ..$ rand88 : num [1:190] 1.15 1.04 1.06 1.54 2.01 ...
.. .. ..$ rand89 : num [1:190] 1.19 1.02 1.05 1.46 1.84 ...
.. .. ..$ rand90 : num [1:190] 1.65 1.39 1.35 1.93 2.29 ...
.. .. ..$ rand91 : num [1:190] 1.26 1.01 1.04 1.39 1.81 ...
.. .. ..$ rand92 : num [1:190] 1.57 1.35 1.3 1.76 2.16 ...
.. .. ..$ rand93 : num [1:190] 1.22 1.09 1.11 1.31 1.74 ...
.. .. ..$ rand94 : num [1:190] 1.4 1.31 1.24 1.78 2.35 ...
.. .. ..$ rand95 : num [1:190] 1.71 1.48 1.36 1.92 2.48 ...
.. .. ..$ rand96 : num [1:190] 1.4 1.23 1.04 1.61 2.02 ...
.. .. ..$ rand97 : num [1:190] 1.47 1.23 1.18 1.5 1.96 ...
.. .. .. [list output truncated]
..$ BC.obs :List of 3
.. ..$ : num [1:20, 1:20] 0 0.0317 0.1653 0.1389 0.051 ...
.. .. ..- attr(\*, "dimnames")=List of 2
.. .. .. ..$ : chr [1:20] "s1" "s2" "s3" "s4" ...
.. .. .. ..$ : chr [1:20] "s1" "s2" "s3" "s4" ...
.. ..$ : num [1:20, 1:20] 0 0.1468 0.0861 0.1444 0.1111 ...
.. .. ..- attr(\*, "dimnames")=List of 2
.. .. .. ..$ : chr [1:20] "s1" "s2" "s3" "s4" ...
.. .. .. ..$ : chr [1:20] "s1" "s2" "s3" "s4" ...
.. ..$ : num [1:20, 1:20] 0 0.345 0.263 0.317 0.494 ...
.. .. ..- attr(\*, "dimnames")=List of 2
.. .. .. ..$ : chr [1:20] "s1" "s2" "s3" "s4" ...
.. .. .. ..$ : chr [1:20] "s1" "s2" "s3" "s4" ...
..$ BCa.rand :List of 3
.. ..$ :'data.frame': 190 obs. of 102 variables:
.. .. ..$ name1 : Factor w/ 19 levels "s10","s11","s12",..: 11 13 14 15 16 17 18 19 1 2 ...
.. .. ..$ name2 : Factor w/ 19 levels "s1","s10","s11",..: 1 1 1 1 1 1 1 1 1 1 ...
.. .. ..$ rand1 : num [1:190] 0.127 0.1931 0.0333 0.137 0.0451 ...
.. .. ..$ rand2 : num [1:190] 0.1429 0.0875 0.0778 0.0465 0.0208 ...
.. .. ..$ rand3 : num [1:190] 0.1071 0.125 0.0667 0.0698 0.0521 ...
.. .. ..$ rand4 : num [1:190] 0.19 0.225 0.206 0.262 0.281 ...
.. .. ..$ rand5 : num [1:190] 0.107 0.296 0.117 0.095 0.104 ...
.. .. ..$ rand6 : num [1:190] 0.123 0.169 0.194 0.148 0.163 ...
.. .. ..$ rand7 : num [1:190] 0 0.1125 0.0889 0.0698 0.0104 ...
.. .. ..$ rand8 : num [1:190] 0.107 0.167 0.178 0.143 0.104 ...
.. .. ..$ rand9 : num [1:190] 0.127 0.1431 0.0778 0.1253 0.0972 ...
.. .. ..$ rand10 : num [1:190] 0.1587 0.1403 0.0278 0.1208 0.0903 ...
.. .. ..$ rand11 : num [1:190] 0.1548 0.1958 0.0944 0.1415 0.0938 ...
.. .. ..$ rand12 : num [1:190] 0.0833 0.0375 0.1111 0.093 0.1562 ...
.. .. ..$ rand13 : num [1:190] 0.111 0.211 0.189 0.123 0.142 ...
.. .. ..$ rand14 : num [1:190] 0.0992 0.1361 0.1556 0.1693 0.2049 ...
.. .. ..$ rand15 : num [1:190] 0.127 0.156 0.1 0.16 0.16 ...
.. .. ..$ rand16 : num [1:190] 0.07937 0.07778 0.00556 0.13243 0.07986 ...
.. .. ..$ rand17 : num [1:190] 0.234 0.16 0.244 0.222 0.253 ...
.. .. ..$ rand18 : num [1:190] 0.0754 0.1653 0.15 0.1441 0.0278 ...
.. .. ..$ rand19 : num [1:190] 0.1905 0.1125 0.1556 0.093 0.0104 ...
.. .. ..$ rand20 : num [1:190] 0.337 0.29 0.3 0.243 0.236 ...
.. .. ..$ rand21 : num [1:190] 0.1706 0.1736 0.1889 0.0995 0.1944 ...
.. .. ..$ rand22 : num [1:190] 0.0952 0.0375 0.1889 0.1395 0.0833 ...
.. .. ..$ rand23 : num [1:190] 0.0595 0.1333 0.1722 0.1531 0.059 ...
.. .. ..$ rand24 : num [1:190] 0.19 0.204 0.178 0.167 0.115 ...
.. .. ..$ rand25 : num [1:190] 0.0952 0.05 0.1889 0.093 0.0417 ...
.. .. ..$ rand26 : num [1:190] 0.246 0.21 0.267 0.257 0.264 ...
.. .. ..$ rand27 : num [1:190] 0.1786 0.2125 0.0222 0.0698 0.125 ...
.. .. ..$ rand28 : num [1:190] 0.2262 0.1292 0.0556 0.1085 0.1875 ...
.. .. ..$ rand29 : num [1:190] 0.119 0.075 0.0778 0.0465 0.1042 ...
.. .. ..$ rand30 : num [1:190] 0.0595 0.075 0.1 0.1163 0.125 ...
.. .. ..$ rand31 : num [1:190] 0.119 0.1 0.167 0.128 0 ...
.. .. ..$ rand32 : num [1:190] 0.131 0.0625 0.1667 0.0814 0.0833 ...
.. .. ..$ rand33 : num [1:190] 0.0119 0.0958 0.1389 0.1996 0.1667 ...
.. .. ..$ rand34 : num [1:190] 0.119 0 0.1444 0.0465 0.125 ...
.. .. ..$ rand35 : num [1:190] 0.23 0.249 0.144 0.111 0.132 ...
.. .. ..$ rand36 : num [1:190] 0.0357 0.15 0.0333 0.0233 0.1146 ...
.. .. ..$ rand37 : num [1:190] 0.0675 0.0681 0.0889 0.1486 0.1389 ...
.. .. ..$ rand38 : num [1:190] 0.0119 0.1 0.1444 0.1279 0.125 ...
.. .. ..$ rand39 : num [1:190] 0.0873 0.1653 0.1278 0.051 0.1528 ...
.. .. ..$ rand40 : num [1:190] 0.246 0.222 0.233 0.257 0.243 ...
.. .. ..$ rand41 : num [1:190] 0.119 0.125 0.0778 0.0814 0.0521 ...
.. .. ..$ rand42 : num [1:190] 0.0714 0.125 0.1333 0.0698 0.1562 ...
.. .. ..$ rand43 : num [1:190] 0.0595 0.1 0.0778 0.0698 0.0729 ...
.. .. ..$ rand44 : num [1:190] 0.1429 0.15 0.0333 0.0814 0.1042 ...
.. .. ..$ rand45 : num [1:190] 0.0476 0.1 0.1444 0.0814 0.0938 ...
.. .. ..$ rand46 : num [1:190] 0.1667 0.075 0.0111 0.093 0.0521 ...
.. .. ..$ rand47 : num [1:190] 0.0317 0.1306 0.1667 0.0904 0.0556 ...
.. .. ..$ rand48 : num [1:190] 0.119 0.1125 0.0667 0.0349 0.1562 ...
.. .. ..$ rand49 : num [1:190] 0.0476 0 0.0444 0.0465 0.1354 ...
.. .. ..$ rand50 : num [1:190] 0.163 0.133 0.161 0.095 0.125 ...
.. .. ..$ rand51 : num [1:190] 0.0595 0.125 0.1 0.0698 0.1042 ...
.. .. ..$ rand52 : num [1:190] 0.1667 0.15 0.0889 0.1628 0 ...
.. .. ..$ rand53 : num [1:190] 0.1667 0 0.0889 0.093 0 ...
.. .. ..$ rand54 : num [1:190] 0.0357 0.125 0.1111 0.1163 0.1354 ...
.. .. ..$ rand55 : num [1:190] 0.0873 0.1167 0.1667 0.1667 0.1562 ...
.. .. ..$ rand56 : num [1:190] 0.1587 0.2736 0.1 0.0762 0.1632 ...
.. .. ..$ rand57 : num [1:190] 0.0556 0.1181 0.0667 0.084 0.0278 ...
.. .. ..$ rand58 : num [1:190] 0.0833 0.1875 0.1 0.1279 0.0938 ...
.. .. ..$ rand59 : num [1:190] 0.1111 0.0736 0.1778 0.0879 0.1632 ...
.. .. ..$ rand60 : num [1:190] 0.1746 0.1639 0.1833 0.0691 0.1424 ...
.. .. ..$ rand61 : num [1:190] 0 0.1375 0.1222 0.093 0.0833 ...
.. .. ..$ rand62 : num [1:190] 0.1151 0.1403 0.0833 0.0536 0.0799 ...
.. .. ..$ rand63 : num [1:190] 0.135 0.178 0.106 0.121 0.17 ...
.. .. ..$ rand64 : num [1:190] 0.0476 0.1 0.1556 0.1047 0.125 ...
.. .. ..$ rand65 : num [1:190] 0.131 0.0375 0.0778 0.0698 0.0208 ...
.. .. ..$ rand66 : num [1:190] 0.25 0.25 0.139 0.18 0.271 ...
.. .. ..$ rand67 : num [1:190] 0.0357 0.1375 0.0667 0.0698 0 ...
.. .. ..$ rand68 : num [1:190] 0.1071 0.0833 0.1056 0.1298 0.1875 ...
.. .. ..$ rand69 : num [1:190] 0.234 0.164 0.172 0.185 0.181 ...
.. .. ..$ rand70 : num [1:190] 0.226 0.204 0.233 0.19 0.198 ...
.. .. ..$ rand71 : num [1:190] 0.0714 0.1125 0.0444 0.093 0.1146 ...
.. .. ..$ rand72 : num [1:190] 0.1508 0.0889 0.1611 0.1156 0.1181 ...
.. .. ..$ rand73 : num [1:190] 0.175 0.226 0.106 0.127 0.191 ...
.. .. ..$ rand74 : num [1:190] 0.0357 0.125 0.1556 0.0233 0.0729 ...
.. .. ..$ rand75 : num [1:190] 0.159 0.14 0.139 0.206 0.309 ...
.. .. ..$ rand76 : num [1:190] 0 0.158 0.106 0.165 0.146 ...
.. .. ..$ rand77 : num [1:190] 0.123 0.161 0.122 0.169 0.163 ...
.. .. ..$ rand78 : num [1:190] 0.0635 0.1611 0.1444 0.2506 0.2153 ...
.. .. ..$ rand79 : num [1:190] 0.0238 0.075 0.0556 0.093 0.1042 ...
.. .. ..$ rand80 : num [1:190] 0.155 0.246 0.106 0.095 0.198 ...
.. .. ..$ rand81 : num [1:190] 0.1706 0.0861 0.1889 0.1227 0.1944 ...
.. .. ..$ rand82 : num [1:190] 0.262 0.154 0.289 0.202 0.135 ...
.. .. ..$ rand83 : num [1:190] 0.127 0.0403 0.1278 0.1208 0.0972 ...
.. .. ..$ rand84 : num [1:190] 0.00397 0.05556 0.11111 0.07881 0.07639 ...
.. .. ..$ rand85 : num [1:190] 0.313 0.278 0.211 0.15 0.267 ...
.. .. ..$ rand86 : num [1:190] 0.23 0.136 0.133 0.204 0.111 ...
.. .. ..$ rand87 : num [1:190] 0 0.1875 0.1444 0.1047 0.0833 ...
.. .. ..$ rand88 : num [1:190] 0.2143 0.125 0.0333 0.1047 0.1771 ...
.. .. ..$ rand89 : num [1:190] 0.333 0.358 0.267 0.403 0.312 ...
.. .. ..$ rand90 : num [1:190] 0.23 0.194 0.261 0.218 0.174 ...
.. .. ..$ rand91 : num [1:190] 0.179 0.167 0.256 0.202 0.167 ...
.. .. ..$ rand92 : num [1:190] 0.0357 0.0375 0.0556 0.1512 0.0729 ...
.. .. ..$ rand93 : num [1:190] 0.0238 0.1625 0.1667 0.1628 0.0833 ...
.. .. ..$ rand94 : num [1:190] 0.131 0.1875 0.0444 0.093 0.0625 ...
.. .. ..$ rand95 : num [1:190] 0.1032 0.1181 0.1222 0.0556 0.1076 ...
.. .. ..$ rand96 : num [1:190] 0.0556 0.0931 0.0778 0.1602 0.1701 ...
.. .. ..$ rand97 : num [1:190] 0.1825 0.1111 0.0778 0.0762 0.059 ...
.. .. .. [list output truncated]
.. ..$ :'data.frame': 190 obs. of 102 variables:
.. .. ..$ name1 : Factor w/ 19 levels "s10","s11","s12",..: 11 13 14 15 16 17 18 19 1 2 ...
.. .. ..$ name2 : Factor w/ 19 levels "s1","s10","s11",..: 1 1 1 1 1 1 1 1 1 1 ...
.. .. ..$ rand1 : num [1:190] 0.1151 0.0306 0.0778 0.137 0.1076 ...
.. .. ..$ rand2 : num [1:190] 0.0397 0.0903 0.0611 0.1324 0.0903 ...
.. .. ..$ rand3 : num [1:190] 0.1032 0.1181 0.0778 0.1137 0.0868 ...
.. .. ..$ rand4 : num [1:190] 0.0635 0.0403 0.0389 0.0627 0.0694 ...
.. .. ..$ rand5 : num [1:190] 0.0833 0 0.0778 0.1047 0.0417 ...
.. .. ..$ rand6 : num [1:190] 0.0238 0.05 0.1222 0.0465 0.0417 ...
.. .. ..$ rand7 : num [1:190] 0.167 0.146 0.106 0.153 0.104 ...
.. .. ..$ rand8 : num [1:190] 0.0476 0.0375 0.0556 0.0116 0.0312 ...
.. .. ..$ rand9 : num [1:190] 0.0516 0.0556 0.1 0.0672 0.0868 ...
.. .. ..$ rand10 : num [1:190] 0.0794 0.0556 0.0444 0.0672 0.066 ...
.. .. ..$ rand11 : num [1:190] 0.0278 0.0278 0.1278 0.1092 0.059 ...
.. .. ..$ rand12 : num [1:190] 0.0595 0.1958 0.1167 0.1415 0.0729 ...
.. .. ..$ rand13 : num [1:190] 0.262 0.167 0.156 0.248 0.281 ...
.. .. ..$ rand14 : num [1:190] 0.0992 0.0861 0.0889 0.0762 0.0694 ...
.. .. ..$ rand15 : num [1:190] 0.1706 0.1361 0.0778 0.146 0.1111 ...
.. .. ..$ rand16 : num [1:190] 0.1349 0.1153 0.05 0.0743 0.0486 ...
.. .. ..$ rand17 : num [1:190] 0.0952 0.0833 0.0722 0.095 0.1042 ...
.. .. ..$ rand18 : num [1:190] 0.0833 0.025 0 0.0698 0.0729 ...
.. .. ..$ rand19 : num [1:190] 0.0119 0.075 0.0333 0.1279 0.0625 ...
.. .. ..$ rand20 : num [1:190] 0.0595 0.0125 0.0889 0.0814 0.0312 ...
.. .. ..$ rand21 : num [1:190] 0.0675 0.0806 0.1222 0.1486 0.0347 ...
.. .. ..$ rand22 : num [1:190] 0.0278 0.0403 0.0278 0.1092 0.1111 ...
.. .. ..$ rand23 : num [1:190] 0.0714 0.0625 0.0333 0.0581 0.0417 ...
.. .. ..$ rand24 : num [1:190] 0.0357 0.05 0.0222 0.0233 0.0625 ...
.. .. ..$ rand25 : num [1:190] 0.0119 0.0375 0.0333 0.0698 0.0729 ...
.. .. ..$ rand26 : num [1:190] 0.127 0.1181 0.1 0.0323 0.0972 ...
.. .. ..$ rand27 : num [1:190] 0.1429 0.0958 0.1056 0.0975 0.1042 ...
.. .. ..$ rand28 : num [1:190] 0.1071 0.0375 0.0444 0.0233 0.0417 ...
.. .. ..$ rand29 : num [1:190] 0.23 0.219 0.139 0.206 0.122 ...
.. .. ..$ rand30 : num [1:190] 0.183 0.186 0.189 0.146 0.111 ...
.. .. ..$ rand31 : num [1:190] 0.0476 0.2333 0.0722 0.1415 0.1563 ...
.. .. ..$ rand32 : num [1:190] 0.187 0.247 0.256 0.234 0.253 ...
.. .. ..$ rand33 : num [1:190] 0.0595 0.0125 0.1 0.0233 0.0312 ...
.. .. ..$ rand34 : num [1:190] 0.0238 0.05 0.0222 0.0465 0 ...
.. .. ..$ rand35 : num [1:190] 0.0952 0.05 0.0667 0.0349 0.1042 ...
.. .. ..$ rand36 : num [1:190] 0.0476 0.075 0.0667 0.1047 0.1146 ...
.. .. ..$ rand37 : num [1:190] 0.0476 0.1 0.1222 0.0698 0.0938 ...
.. .. ..$ rand38 : num [1:190] 0.19 0.179 0.144 0.134 0.188 ...
.. .. ..$ rand39 : num [1:190] 0 0 0.0889 0.0233 0.0104 ...
.. .. ..$ rand40 : num [1:190] 0.04365 0.06806 0.05556 0.09044 0.00347 ...
.. .. ..$ rand41 : num [1:190] 0.0119 0.0375 0.0111 0.1047 0.0729 ...
.. .. ..$ rand42 : num [1:190] 0.214 0.275 0.183 0.192 0.25 ...
.. .. ..$ rand43 : num [1:190] 0.0913 0.0931 0.0556 0.137 0.1181 ...
.. .. ..$ rand44 : num [1:190] 0.1587 0.0861 0.1111 0.0762 0.0694 ...
.. .. ..$ rand45 : num [1:190] 0.143 0.167 0.189 0.178 0.26 ...
.. .. ..$ rand46 : num [1:190] 0.0278 0.0403 0.0389 0.0627 0.0382 ...
.. .. ..$ rand47 : num [1:190] 0.0357 0.0625 0.0222 0.1137 0.0208 ...
.. .. ..$ rand48 : num [1:190] 0.131 0.0708 0.1611 0.095 0.1042 ...
.. .. ..$ rand49 : num [1:190] 0 0.075 0.0111 0.0465 0 ...
.. .. ..$ rand50 : num [1:190] 0.119 0 0.0222 0.093 0.0625 ...
.. .. ..$ rand51 : num [1:190] 0.1071 0.0625 0.0222 0.0349 0 ...
.. .. ..$ rand52 : num [1:190] 0.00794 0.10278 0.08333 0.06266 0.10069 ...
.. .. ..$ rand53 : num [1:190] 0.123 0.0861 0.1222 0.1227 0.2569 ...
.. .. ..$ rand54 : num [1:190] 0.1071 0.0681 0.0778 0.0439 0.1042 ...
.. .. ..$ rand55 : num [1:190] 0.0357 0.0875 0.0889 0.0116 0.0208 ...
.. .. ..$ rand56 : num [1:190] 0.0833 0.0458 0.0833 0.1298 0.0833 ...
.. .. ..$ rand57 : num [1:190] 0.0595 0.0625 0.0667 0.0698 0.0521 ...
.. .. ..$ rand58 : num [1:190] 0.135 0.136 0.1 0.123 0.101 ...
.. .. ..$ rand59 : num [1:190] 0.1071 0.0833 0.0944 0.095 0.0938 ...
.. .. ..$ rand60 : num [1:190] 0.0119 0 0.0333 0.0581 0.0104 ...
.. .. ..$ rand61 : num [1:190] 0.1984 0.1514 0.0944 0.1389 0.1285 ...
.. .. ..$ rand62 : num [1:190] 0.0833 0.0528 0.0944 0.1182 0.184 ...
.. .. ..$ rand63 : num [1:190] 0.119 0.1083 0.1944 0.0368 0.0729 ...
.. .. ..$ rand64 : num [1:190] 0.0556 0.0931 0.0556 0.0788 0.0868 ...
.. .. ..$ rand65 : num [1:190] 0.0357 0.0875 0.0333 0.0465 0.0312 ...
.. .. ..$ rand66 : num [1:190] 0.123 0.1528 0.1278 0.051 0.0278 ...
.. .. ..$ rand67 : num [1:190] 0.0992 0.0528 0.1056 0.0743 0.1111 ...
.. .. ..$ rand68 : num [1:190] 0.0119 0.025 0.0556 0.0349 0.0729 ...
.. .. ..$ rand69 : num [1:190] 0.0754 0.1111 0.1556 0.1344 0.1007 ...
.. .. ..$ rand70 : num [1:190] 0.0595 0.0375 0.1 0.0698 0.0417 ...
.. .. ..$ rand71 : num [1:190] 0.0238 0.025 0.1 0.0233 0.0833 ...
.. .. ..$ rand72 : num [1:190] 0.1468 0.1111 0.0778 0.0995 0.0799 ...
.. .. ..$ rand73 : num [1:190] 0.0119 0.05 0 0.0116 0.125 ...
.. .. ..$ rand74 : num [1:190] 0.0873 0.0528 0.05 0.1001 0.0139 ...
.. .. ..$ rand75 : num [1:190] 0.0952 0.0833 0.0611 0.1066 0.0521 ...
.. .. ..$ rand76 : num [1:190] 0.0714 0 0.1333 0.0581 0.0521 ...
.. .. ..$ rand77 : num [1:190] 0 0.0125 0 0.0581 0 ...
.. .. ..$ rand78 : num [1:190] 0.0119 0.0125 0.0556 0 0.0208 ...
.. .. ..$ rand79 : num [1:190] 0.119 0.075 0.0778 0.0698 0.0208 ...
.. .. ..$ rand80 : num [1:190] 0.0992 0.0278 0.0389 0.0627 0.0174 ...
.. .. ..$ rand81 : num [1:190] 0 0.0625 0.0556 0.1163 0.0521 ...
.. .. ..$ rand82 : num [1:190] 0.0873 0.1486 0.1333 0.1111 0.1528 ...
.. .. ..$ rand83 : num [1:190] 0.0476 0.05 0.0556 0.0581 0.0417 ...
.. .. ..$ rand84 : num [1:190] 0.0833 0.0625 0 0.0116 0 ...
.. .. ..$ rand85 : num [1:190] 0.0992 0.0403 0.1056 0.051 0.059 ...
.. .. ..$ rand86 : num [1:190] 0.0952 0.1208 0.1167 0.0601 0.125 ...
.. .. ..$ rand87 : num [1:190] 0.0357 0.0375 0.0667 0 0.0417 ...
.. .. ..$ rand88 : num [1:190] 0.0952 0.075 0.1111 0.0116 0.0312 ...
.. .. ..$ rand89 : num [1:190] 0.0833 0.1125 0.0667 0.0581 0.0312 ...
.. .. ..$ rand90 : num [1:190] 0.1071 0.1125 0.0667 0.0814 0 ...
.. .. ..$ rand91 : num [1:190] 0.0556 0.1181 0.1333 0.0672 0.0972 ...
.. .. ..$ rand92 : num [1:190] 0 0.025 0.0778 0.0233 0.0625 ...
.. .. ..$ rand93 : num [1:190] 0 0.0625 0.0222 0.0581 0.1354 ...
.. .. ..$ rand94 : num [1:190] 0 0.025 0.0111 0.0581 0 ...
.. .. ..$ rand95 : num [1:190] 0.1429 0.1083 0.0611 0.1647 0.1146 ...
.. .. ..$ rand96 : num [1:190] 0.119 0.075 0.0667 0.0233 0.0938 ...
.. .. ..$ rand97 : num [1:190] 0.0833 0 0.0333 0.0349 0 ...
.. .. .. [list output truncated]
.. ..$ :'data.frame': 190 obs. of 102 variables:
.. .. ..$ name1 : Factor w/ 19 levels "s10","s11","s12",..: 11 13 14 15 16 17 18 19 1 2 ...
.. .. ..$ name2 : Factor w/ 19 levels "s1","s10","s11",..: 1 1 1 1 1 1 1 1 1 1 ...
.. .. ..$ rand1 : num [1:190] 0.639 0.526 0.511 0.563 0.535 ...
.. .. ..$ rand2 : num [1:190] 0.603 0.572 0.683 0.561 0.618 ...
.. .. ..$ rand3 : num [1:190] 0.742 0.557 0.833 0.607 0.542 ...
.. .. ..$ rand4 : num [1:190] 0.627 0.41 0.444 0.397 0.649 ...
.. .. ..$ rand5 : num [1:190] 0.714 0.704 0.561 0.614 0.583 ...
.. .. ..$ rand6 : num [1:190] 0.409 0.319 0.394 0.457 0.538 ...
.. .. ..$ rand7 : num [1:190] 0.405 0.517 0.517 0.545 0.427 ...
.. .. ..$ rand8 : num [1:190] 0.456 0.696 0.489 0.58 0.58 ...
.. .. ..$ rand9 : num [1:190] 0.591 0.426 0.411 0.389 0.649 ...
.. .. ..$ rand10 : num [1:190] 0.667 0.629 0.461 0.533 0.427 ...
.. .. ..$ rand11 : num [1:190] 0.365 0.626 0.367 0.307 0.368 ...
.. .. ..$ rand12 : num [1:190] 0.762 0.642 0.683 0.626 0.708 ...
.. .. ..$ rand13 : num [1:190] 0.413 0.497 0.478 0.513 0.556 ...
.. .. ..$ rand14 : num [1:190] 0.635 0.553 0.533 0.452 0.58 ...
.. .. ..$ rand15 : num [1:190] 0.488 0.458 0.622 0.554 0.604 ...
.. .. ..$ rand16 : num [1:190] 0.587 0.632 0.644 0.561 0.726 ...
.. .. ..$ rand17 : num [1:190] 0.337 0.532 0.483 0.45 0.455 ...
.. .. ..$ rand18 : num [1:190] 0.484 0.585 0.583 0.647 0.649 ...
.. .. ..$ rand19 : num [1:190] 0.631 0.662 0.5 0.453 0.802 ...
.. .. ..$ rand20 : num [1:190] 0.603 0.406 0.389 0.513 0.649 ...
.. .. ..$ rand21 : num [1:190] 0.762 0.596 0.6 0.612 0.729 ...
.. .. ..$ rand22 : num [1:190] 0.544 0.45 0.628 0.495 0.597 ...
.. .. ..$ rand23 : num [1:190] 0.393 0.479 0.394 0.417 0.406 ...
.. .. ..$ rand24 : num [1:190] 0.679 0.696 0.644 0.554 0.49 ...
.. .. ..$ rand25 : num [1:190] 0.79 0.487 0.6 0.558 0.677 ...
.. .. ..$ rand26 : num [1:190] 0.484 0.547 0.411 0.594 0.389 ...
.. .. ..$ rand27 : num [1:190] 0.44 0.417 0.583 0.48 0.521 ...
.. .. ..$ rand28 : num [1:190] 0.27 0.522 0.322 0.455 0.333 ...
.. .. ..$ rand29 : num [1:190] 0.389 0.606 0.472 0.492 0.42 ...
.. .. ..$ rand30 : num [1:190] 0.702 0.683 0.567 0.627 0.479 ...
.. .. ..$ rand31 : num [1:190] 0.476 0.442 0.361 0.364 0.594 ...
.. .. ..$ rand32 : num [1:190] 0.516 0.59 0.489 0.499 0.497 ...
.. .. ..$ rand33 : num [1:190] 0.643 0.586 0.339 0.591 0.427 ...
.. .. ..$ rand34 : num [1:190] 0.5 0.492 0.433 0.563 0.639 ...
.. .. ..$ rand35 : num [1:190] 0.317 0.551 0.678 0.528 0.326 ...
.. .. ..$ rand36 : num [1:190] 0.694 0.669 0.344 0.5 0.604 ...
.. .. ..$ rand37 : num [1:190] 0.623 0.632 0.567 0.596 0.663 ...
.. .. ..$ rand38 : num [1:190] 0.393 0.621 0.489 0.45 0.583 ...
.. .. ..$ rand39 : num [1:190] 0.389 0.413 0.494 0.657 0.58 ...
.. .. ..$ rand40 : num [1:190] 0.425 0.535 0.467 0.513 0.462 ...
.. .. ..$ rand41 : num [1:190] 0.623 0.713 0.478 0.558 0.625 ...
.. .. ..$ rand42 : num [1:190] 0.452 0.575 0.417 0.576 0.427 ...
.. .. ..$ rand43 : num [1:190] 0.452 0.482 0.6 0.514 0.434 ...
.. .. ..$ rand44 : num [1:190] 0.389 0.289 0.656 0.54 0.701 ...
.. .. ..$ rand45 : num [1:190] 0.738 0.458 0.622 0.671 0.542 ...
.. .. ..$ rand46 : num [1:190] 0.528 0.474 0.628 0.663 0.569 ...
.. .. ..$ rand47 : num [1:190] 0.52 0.576 0.544 0.494 0.681 ...
.. .. ..$ rand48 : num [1:190] 0.464 0.642 0.539 0.521 0.406 ...
.. .. ..$ rand49 : num [1:190] 0.579 0.925 0.611 0.767 0.615 ...
.. .. ..$ rand50 : num [1:190] 0.552 0.511 0.394 0.431 0.215 ...
.. .. ..$ rand51 : num [1:190] 0.69 0.812 0.811 0.826 0.812 ...
.. .. ..$ rand52 : num [1:190] 0.413 0.747 0.561 0.542 0.378 ...
.. .. ..$ rand53 : num [1:190] 0.567 0.714 0.544 0.366 0.514 ...
.. .. ..$ rand54 : num [1:190] 0.635 0.446 0.656 0.654 0.469 ...
.. .. ..$ rand55 : num [1:190] 0.655 0.546 0.656 0.473 0.469 ...
.. .. ..$ rand56 : num [1:190] 0.615 0.506 0.572 0.631 0.628 ...
.. .. ..$ rand57 : num [1:190] 0.48 0.708 0.444 0.572 0.601 ...
.. .. ..$ rand58 : num [1:190] 0.472 0.601 0.544 0.61 0.618 ...
.. .. ..$ rand59 : num [1:190] 0.496 0.501 0.483 0.399 0.264 ...
.. .. ..$ rand60 : num [1:190] 0.552 0.611 0.406 0.617 0.674 ...
.. .. ..$ rand61 : num [1:190] 0.516 0.536 0.406 0.605 0.538 ...
.. .. ..$ rand62 : num [1:190] 0.603 0.626 0.533 0.563 0.556 ...
.. .. ..$ rand63 : num [1:190] 0.484 0.514 0.567 0.726 0.41 ...
.. .. ..$ rand64 : num [1:190] 0.468 0.357 0.433 0.514 0.559 ...
.. .. ..$ rand65 : num [1:190] 0.548 0.625 0.511 0.628 0.573 ...
.. .. ..$ rand66 : num [1:190] 0.46 0.272 0.511 0.443 0.535 ...
.. .. ..$ rand67 : num [1:190] 0.627 0.685 0.606 0.582 0.611 ...
.. .. ..$ rand68 : num [1:190] 0.476 0.617 0.439 0.486 0.531 ...
.. .. ..$ rand69 : num [1:190] 0.429 0.375 0.428 0.494 0.51 ...
.. .. ..$ rand70 : num [1:190] 0.476 0.458 0.5 0.554 0.531 ...
.. .. ..$ rand71 : num [1:190] 0.683 0.751 0.367 0.628 0.483 ...
.. .. ..$ rand72 : num [1:190] 0.44 0.25 0.517 0.483 0.531 ...
.. .. ..$ rand73 : num [1:190] 0.552 0.557 0.361 0.443 0.392 ...
.. .. ..$ rand74 : num [1:190] 0.496 0.767 0.506 0.635 0.524 ...
.. .. ..$ rand75 : num [1:190] 0.46 0.46 0.6 0.571 0.368 ...
.. .. ..$ rand76 : num [1:190] 0.643 0.567 0.639 0.498 0.594 ...
.. .. ..$ rand77 : num [1:190] 0.56 0.596 0.578 0.531 0.385 ...
.. .. ..$ rand78 : num [1:190] 0.615 0.601 0.522 0.578 0.542 ...
.. .. ..$ rand79 : num [1:190] 0.595 0.583 0.511 0.651 0.667 ...
.. .. ..$ rand80 : num [1:190] 0.675 0.551 0.511 0.633 0.472 ...
.. .. ..$ rand81 : num [1:190] 0.425 0.576 0.556 0.459 0.399 ...
.. .. ..$ rand82 : num [1:190] 0.579 0.597 0.511 0.548 0.545 ...
.. .. ..$ rand83 : num [1:190] 0.437 0.585 0.572 0.635 0.618 ...
.. .. ..$ rand84 : num [1:190] 0.611 0.432 0.733 0.63 0.569 ...
.. .. ..$ rand85 : num [1:190] 0.587 0.507 0.461 0.52 0.382 ...
.. .. ..$ rand86 : num [1:190] 0.413 0.393 0.55 0.48 0.535 ...
.. .. ..$ rand87 : num [1:190] 0.472 0.45 0.522 0.616 0.493 ...
.. .. ..$ rand88 : num [1:190] 0.595 0.675 0.489 0.442 0.604 ...
.. .. ..$ rand89 : num [1:190] 0.583 0.454 0.489 0.516 0.552 ...
.. .. ..$ rand90 : num [1:190] 0.433 0.593 0.606 0.488 0.535 ...
.. .. ..$ rand91 : num [1:190] 0.353 0.615 0.567 0.429 0.66 ...
.. .. ..$ rand92 : num [1:190] 0.702 0.562 0.511 0.547 0.552 ...
.. .. ..$ rand93 : num [1:190] 0.548 0.65 0.5 0.523 0.559 ...
.. .. ..$ rand94 : num [1:190] 0.536 0.487 0.878 0.57 0.812 ...
.. .. ..$ rand95 : num [1:190] 0.635 0.674 0.617 0.64 0.715 ...
.. .. ..$ rand96 : num [1:190] 0.444 0.54 0.344 0.584 0.528 ...
.. .. ..$ rand97 : num [1:190] 0.409 0.428 0.378 0.517 0.33 ...
.. .. .. [list output truncated]
$ special.crct:List of 1
..$ SigbMPDi:List of 3
.. ..$ special.ses :'data.frame': 190 obs. of 5 variables:
.. .. ..$ name1: Factor w/ 19 levels "s10","s11","s12",..: 11 13 14 15 16 17 18 19 1 2 ...
.. .. ..$ name2: Factor w/ 19 levels "s1","s10","s11",..: 1 1 1 1 1 1 1 1 1 1 ...
.. .. ..$ bin1 : num [1:190] 0 0 0 0 0 0 0 0 0 0 ...
.. .. ..$ bin2 : num [1:190] 0 0 0 0 0 0 0 -99 0 0 ...
.. .. ..$ bin3 : num [1:190] 0 0 0 0 0 0 0 0 0 0 ...
.. ..$ special.rc :'data.frame': 190 obs. of 5 variables:
.. .. ..$ name1: Factor w/ 19 levels "s10","s11","s12",..: 11 13 14 15 16 17 18 19 1 2 ...
.. .. ..$ name2: Factor w/ 19 levels "s1","s10","s11",..: 1 1 1 1 1 1 1 1 1 1 ...
.. .. ..$ bin1 : num [1:190] 0 0 0 0 0 0 0 0 0 0 ...
.. .. ..$ bin2 : num [1:190] 0 0 0 0 0 0 0 -1.1 0 0 ...
.. .. ..$ bin3 : num [1:190] 0 0 0 0 0 0 0 0 0 0 ...
.. ..$ special.conf:'data.frame': 190 obs. of 5 variables:
.. .. ..$ name1: Factor w/ 19 levels "s10","s11","s12",..: 11 13 14 15 16 17 18 19 1 2 ...
.. .. ..$ name2: Factor w/ 19 levels "s1","s10","s11",..: 1 1 1 1 1 1 1 1 1 1 ...
.. .. ..$ bin1 : num [1:190] 0 0 0 0 0 0 0 0 0 0 ...
.. .. ..$ bin2 : num [1:190] 0 0 0 0 0 0 0 -1.1 0 0 ...
.. .. ..$ bin3 : num [1:190] 0 0 0 0 0 0 0 0 0 0 ...

### Details

See help of icamp.big for detail.

### Source

icamp.big result from the example.data.

### References

Ning, D., Yuan, M., Wu, L., Zhang, Y., Guo, X., Zhou, X. et al. (2020). A quantitative framework reveals the ecological drivers of grassland soil microbial community assembly in response to warming. bioRxiv. doi:10.1101/2020.02.22.960872 (Nature Communications, final revision).

### Examples

```
data(icamp.out)
```

---

[Package *iCAMP* version 1.2.4 Index]
