# Supplementary material for: A quantitative framework reveals ecological drivers of grassland microbial community assembly in response to warming
Source: Nat Commun. 2020 Sep 18;11:4717. doi: 10.1038/s41467-020-18560-z (PMC7501310; doi:10.1038/s41467-020-18560-z)
Supplement: Supplementary file 5 — Supplementary Code 1 [file 41467_2020_18560_MOESM5_ESM.zip › iCAMP_1.2.4.zip!/iCAMP/html/match.2col.html]

R: Check the consistency of the first two columns of different...

|  |  |
| --- | --- |
| match.2col {iCAMP} | R Documentation |

## Check the consistency of the first two columns of different matrixes

### Description

This function is usually used to check the consistency of samples names in different pairwise comparison matrixes.

### Usage

```
match.2col(check.list, name.check = NULL, rerank = TRUE, silent = FALSE)
```

### Arguments

|  |  |
| --- | --- |
| `check.list` | List, each element is a matrix. It must be set in a format like "check.list=list(A=A,B=B)". The first two columns of the matrixes will be compared and matched with each other. |
| `name.check` | matrix, the first two columns will be used as a standard. The pairs not appear in this matrix will be removed from all matrixes. |
| `rerank` | Logic, make the first two columns in all matrixes in the same rank or not. Default is TRUE. |
| `silent` | Logic, whether to show messages. Default is FALSE, thus all messages will be showed. |

### Details

A tool to match IDs.

### Value

Return a list object, new matrixes with the same first two columns. Some messages will return if some names are removed or all names matches very well.

### Note

Version 2: 2020.8.19, add example.
Version 1: 2018.10.20

### Author(s)

Daliang Ning

### Examples

```
# here two simple matrixes are generated and the pairwise comparison IDs not matched are removed.
A=1:5
names(A)=paste0("S",1:5)
B=1:6
names(B)=paste0("S",1:6)
DA3c=dist.3col(dist(A))
DB3c=dist.3col(dist(B))

checkid=match.2col(check.list = list(DA3c=DA3c,DB3c=DB3c))
DA3cnew=checkid$DA3c
DB3cnew=checkid$DB3c
```

---

[Package *iCAMP* version 1.2.4 Index]
