# Supplementary material for: A quantitative framework reveals ecological drivers of grassland microbial community assembly in response to warming
Source: Nat Commun. 2020 Sep 18;11:4717. doi: 10.1038/s41467-020-18560-z (PMC7501310; doi:10.1038/s41467-020-18560-z)
Supplement: Supplementary file 5 — Supplementary Code 1 [file 41467_2020_18560_MOESM5_ESM.zip › iCAMP_1.2.4.zip!/iCAMP/html/match.name.html]

R: Check and ensure the consistency of IDs in different objects.

|  |  |
| --- | --- |
| match.name {iCAMP} | R Documentation |

## Check and ensure the consistency of IDs in different objects.

### Description

This function is usually used to check the consistency of species or samples names in different data table (e.g. OTU table and phylogenetic distance matrix). it can be used to check row names and/or column names of different matrixes, names in vector(s) or list(s), and tip.lable in tree(s)

### Usage

```
match.name(name.check=integer(0), rn.list=list(integer(0)),
           cn.list=list(integer(0)), both.list=list(integer(0)),
           v.list=list(integer(0)), lf.list=list(integer(0)),
           tree.list=list(integer(0)), group=integer(0),
           rerank=TRUE, silent=FALSE)
```

### Arguments

|  |  |
| --- | --- |
| `name.check` | A character vector, indicating reference name list or the names you would like to keep. If not available, a union of all names is set as reference name list. |
| `rn.list` | A list object, including the matrix(es) of which the row names will be check. rn.list must be set in a format like "rn.list=list(A=A,B=B)". default is nothing. |
| `cn.list` | A list object, including the matrix(es) of which the column names will be check. cn.list must be set in a format like "cn.list=list(A=A,B=B)". default is nothing. |
| `both.list` | A list object, including the matrix(es) of which both column and row names will be check. both.list must be set in a format like "both.list=list(A=A,B=B)". default is nothing. |
| `v.list` | A list object, including the vector(s) of which the names will be check. v.list must be set in a format like "v.list=list(A=A,B=B)".default is nothing. |
| `lf.list` | A list object, including the list(s) of which the names will be check. lf.list must be set in a format like "lf.list=list(A=A,B=B)".default is nothing. |
| `tree.list` | A list object, including the tree(s) of which the tip.label names will be check. tree.list must be set in a format like "tree.list=list(A=A,B=B)".default is nothing. |
| `group` | a vector or one-column matrix/data.frame indicating the grouping information of samples or species, of which the sample/species names will be check. |
| `rerank` | Logic, make all names in the same rank or not. Default is TRUE |
| `silent` | Logic, whether to show messages. Default is FALSE, thus all messages will be showed. |

### Details

In many cases and functions, species names and samples names must be checked and set in the same rank. Sometimes, we also need to select some samples or species as necessary. This function can help.

### Value

Return a list object, new matrixes with the same row/column names in the same rank.
Some messages will return if some names are removed or all names match very well.

### Note

Version 3: 2017.3.13
Version 2: 2015.9.25

### Author(s)

Daliang Ning

### Examples

```
data("example.data")
comm=example.data$comm
treat=example.data$treat
tree=example.data$tree
pd=example.data$pd
clas=example.data$classification

env=example.data$env
# remove one sample in purpose to see how match.name works
env=env[-13,]

sampid.check=match.name(rn.list = list(comm=comm, treat=treat, env=env))
comm.ck=sampid.check$comm
comm.ck=comm.ck[,colSums(comm.ck)>0,drop=FALSE]
treat.ck=sampid.check$treat
env.ck=sampid.check$env

taxid.check=match.name(cn.list = list(comm.ck=comm.ck),
                       rn.list = list(clas=clas),
                       tree.list = list(tree=tree))
comm.ck=taxid.check$comm.ck
clas.ck=taxid.check$clas
tree.ck=taxid.check$tree
```

---

[Package *iCAMP* version 1.2.4 Index]
