# Supplementary material for: A quantitative framework reveals ecological drivers of grassland microbial community assembly in response to warming
Source: Nat Commun. 2020 Sep 18;11:4717. doi: 10.1038/s41467-020-18560-z (PMC7501310; doi:10.1038/s41467-020-18560-z)
Supplement: Supplementary file 5 — Supplementary Code 1 [file 41467_2020_18560_MOESM5_ESM.zip › iCAMP_1.2.4.zip!/iCAMP/html/maxbigm.html]

R: Find maximum value in a big matrix

|  |  |
| --- | --- |
| maxbigm {iCAMP} | R Documentation |

## Find maximum value in a big matrix

### Description

Return the maxium value and its (their) location(s) in a big matrix.

### Usage

```
maxbigm(m.desc, m.wd, nworker = 1, rm.na = TRUE, size.limit = 10000 * 10000)
```

### Arguments

|  |  |
| --- | --- |
| `m.desc` | the name of the file to hold the backingfile description of the big matrix. |
| `m.wd` | the path of the folder holding the big matrix file. |
| `nworker` | integer, for parallel computing. Either a character vector of host names on which to run the worker copies of R, or a positive integer (in which case that number of copies is run on localhost). default is 4, means 4 threads will be run. |
| `rm.na` | logic, whether to remove NA. Default is TRUE. |
| `size.limit` | the matrix size which your current computer memory can easily handle at each time. |

### Details

A tool to figure out the maximum value in the big phylogenetic distance matrix.

### Value

Output is a list of two elements.

|  |  |
| --- | --- |
| `max.value` | Numeric, the maximum value. |
| `row.col` | Matrix, the row(s) and column(s), i.e. the location(s), of the maximum value in the big matrix. |

### Note

Version 2: 2020.8.19, add example.
Version 1: 2015.12.16

### Author(s)

Daliang Ning

### References

Michael J. Kane, John Emerson, Stephen Weston (2013). Scalable Strategies for Computing with Massive Data. Journal of Statistical Software, 55(14), 1-19. URL http://www.jstatsoft.org/v55/i14/.

### See Also

`midpoint.root.big`

### Examples

```
# this example shows how to find maximum value
# in a big phylogenetic distance matrix.
data("example.data")
tree=example.data$tree
# since pdist.big need to save output to a certain folder,
# the following code is set as 'not run'.
# but you may test the code on your computer
# after change the folder path for 'save.wd'.
## Not run: 
  save.wd="C:/test/" # please change to the folder you want to save the pd.big output.
  nworker=2 # parallel computing thread number
  pd.big=pdist.big(tree = tree, wd=save.wd, nworker = nworker)
  
  maxb=maxbigm(m.desc = pd.big$pd.file, m.wd = pd.big$pd.wd,
               nworker = nworker, rm.na = TRUE)

## End(Not run)
```

---

[Package *iCAMP* version 1.2.4 Index]
