# Supplementary material for: A quantitative framework reveals ecological drivers of grassland microbial community assembly in response to warming
Source: Nat Commun. 2020 Sep 18;11:4717. doi: 10.1038/s41467-020-18560-z (PMC7501310; doi:10.1038/s41467-020-18560-z)
Supplement: Supplementary file 5 — Supplementary Code 1 [file 41467_2020_18560_MOESM5_ESM.zip › iCAMP_1.2.4.zip!/iCAMP/html/midpoint.root.big.html]

R: Midpoint root a large phylogeny

|  |  |
| --- | --- |
| midpoint.root.big {iCAMP} | R Documentation |

## Midpoint root a large phylogeny

### Description

This is modified from the function "modpoint.root" in package "phytools". To deal with a large tree, phylogenetic distance is calculated and saved by using bigmemory in advance.

### Usage

```
midpoint.root.big(tree, pd.desc, pd.spname, pd.wd, nworker = 4)
```

### Arguments

|  |  |
| --- | --- |
| `tree` | phylogenetic tree, an object of class "phylo". |
| `pd.desc` | the name of the file to hold the backingfile description of the phylogenetic distance matrix, it is usually "pd.desc" if using default setting in pdist.big function. |
| `pd.spname` | character vector, taxa id in the same rank as the big matrix of phylogenetic distances. |
| `pd.wd` | folder path, where the bigmemmory file of the phylogenetic distance matrix are saved. |
| `nworker` | integer, for parallel computing. Either a character vector of host names on which to run the worker copies of R, or a positive integer (in which case that number of copies is run on localhost). default is 4, means 4 threads will be run. |

### Details

iCAMP analysis need a rooted tree. If it is difficult to figure out the root, midpoint root is recommended for iCAMP analysis. Modified from the function 'midpoint.root' in package 'phytool'(Revell 2012), this function uses bigmemory (Kane et al 2013) to deal with large datasets.

### Value

Output is a list with two elements.

|  |  |
| --- | --- |
| `tree` | The rooted tree. |
| `max.pd` | The maximum pairwise phylogenetic distance. |

### Note

Version 2: 2020.8.19, update help document, add example.
Version 1: 2015.12.16

### Author(s)

Daliang Ning

### References

Farris, J. (1972) Estimating phylogenetic trees from distance matrices. American Naturalist, 106, 645-667.

Paradis, E., J. Claude, and K. Strimmer (2004) APE: Analyses of phylogenetics and evolution in R language. Bioinformatics, 20, 289-290.

Revell, L. J. (2012) phytools: An R package for phylogenetic comparative biology (and other things). Methods Ecol. Evol., 3, 217-223.

Michael J. Kane, John Emerson, Stephen Weston (2013). Scalable Strategies for Computing with Massive Data. Journal of Statistical Software, 55(14), 1-19. URL http://www.jstatsoft.org/v55/i14/.

### See Also

`maxbigm`

### Examples

```
data("example.data")
tree=example.data$tree
# since pdist.big need to save output to a certain folder,
# the following code is set as 'not run'.
# but you may test the code on your computer
# after change the folder path for 'save.wd'.
## Not run: 
  save.wd="C:/test/" # please change to the folder you want to save the pd.big output.
  nworker=2 # parallel computing thread number
  pd.big=pdist.big(tree = tree, wd=save.wd, nworker = nworker)
  
  mroot=midpoint.root.big(tree = tree, pd.desc = pd.big$pd.file,
                          pd.spname = pd.big$tip.label,
                          pd.wd = pd.big$pd.wd, nworker = nworker)

## End(Not run)
```

---

[Package *iCAMP* version 1.2.4 Index]
