# Supplementary material for: A quantitative framework reveals ecological drivers of grassland microbial community assembly in response to warming
Source: Nat Commun. 2020 Sep 18;11:4717. doi: 10.1038/s41467-020-18560-z (PMC7501310; doi:10.1038/s41467-020-18560-z)
Supplement: Supplementary file 5 — Supplementary Code 1 [file 41467_2020_18560_MOESM5_ESM.zip › iCAMP_1.2.4.zip!/iCAMP/html/mntdn.html]

R: Mean nearest taxon distance (MNTD)

|  |  |
| --- | --- |
| mntdn {iCAMP} | R Documentation |

## Mean nearest taxon distance (MNTD)

### Description

Calculate mean nearest taxon distance (MNTD) in each community in a given community matrix.

### Usage

```
mntdn(comm, pd, abundance.weighted = TRUE,
      check.name = TRUE, memory.G = 50, time.count = FALSE)
```

### Arguments

|  |  |
| --- | --- |
| `comm` | matrix or data.frame, community data matrix, rownames are sample names, colnames are OTU ids. |
| `pd` | matrix, pairwise phylogenetic distance matrix. |
| `abundance.weighted` | logic, whether weighted by species abundance, default is TRUE, means weighted. |
| `check.name` | logic, whether to check the OTU ids (species names) in community matrix and phylogenetic distance matrix are the same. |
| `memory.G` | numeric, to set the memory size as you need, so that calculation of large tree will not be limited by physical memory. unit is Gb. default is 50Gb |
| `time.count` | logic, whether to count calculation time, default is FALSE. |

### Details

mean nearest taxon distance (MNTD) in each community, using the same algrithm as the function 'mntd' in package 'picante'.

### Value

result is a numeric vector with sample names

### Note

Version 2: 2020.8.19, update help document, add example.
Version 1: 2017.3.13

### Author(s)

Daliang Ning

### References

Webb CO, Ackerly DD, and Kembel SW. (2008). Phylocom: software for the analysis of phylogenetic community structure and trait evolution. Bioinformatics 18:2098-2100

Kembel, S.W., Cowan, P.D., Helmus, M.R., Cornwell, W.K., Morlon, H., Ackerly, D.D. et al. (2010). Picante: R tools for integrating phylogenies and ecology. Bioinformatics, 26, 1463-1464.

### See Also

`NTI.p`

### Examples

```
data("example.data")
comm=example.data$comm
pd=example.data$pd
mntd=mntdn(comm=comm,pd=pd,abundance.weighted = TRUE)
```

---

[Package *iCAMP* version 1.2.4 Index]
