# Supplementary material for: A quantitative framework reveals ecological drivers of grassland microbial community assembly in response to warming
Source: Nat Commun. 2020 Sep 18;11:4717. doi: 10.1038/s41467-020-18560-z (PMC7501310; doi:10.1038/s41467-020-18560-z)
Supplement: Supplementary file 5 — Supplementary Code 1 [file 41467_2020_18560_MOESM5_ESM.zip › iCAMP_1.2.4.zip!/iCAMP/html/mpdn.html]

R: Mean pairwise distance (MPD)

|  |  |
| --- | --- |
| mpdn {iCAMP} | R Documentation |

## Mean pairwise distance (MPD)

### Description

Calculate mean pairwise distance (MPD) in each community in a given community matrix.

### Usage

```
mpdn(comm, pd, abundance.weighted = TRUE, time.output = FALSE)
```

### Arguments

|  |  |
| --- | --- |
| `comm` | matrix or data.frame, community data matrix, rownames are sample names, colnames are OTU ids. |
| `pd` | matrix, pairwise phylogenetic distance matrix. |
| `abundance.weighted` | logic, whether weighted by species abundance, default is TRUE, means weighted. |
| `time.output` | logic, whether to count calculation time, default is FALSE. |

### Details

mean pairwise distance (MPD) in each community, which is the same index as 'mpd' in package 'picante', but calculated by matrix multiplication.

### Value

result is a numeric vector with sample names

### Note

Version 2: 2020.8.19, update help document, add example.
Version 1: 2017.3.13

### Author(s)

Daliang Ning

### References

Webb C, Ackerly D, McPeek M, and Donoghue M. (2002). Phylogenies and community ecology. Annual Review of Ecology and Systematics 33:475-505.

Kembel, S.W., Cowan, P.D., Helmus, M.R., Cornwell, W.K., Morlon, H., Ackerly, D.D. et al. (2010). Picante: R tools for integrating phylogenies and ecology. Bioinformatics, 26, 1463-1464.

### See Also

`NRI.p`

### Examples

```
data("example.data")
comm=example.data$comm
pd=example.data$pd
mpd=mpdn(comm = comm, pd = pd, abundance.weighted = TRUE)
```

---

[Package *iCAMP* version 1.2.4 Index]
