# Supplementary material for: A quantitative framework reveals ecological drivers of grassland microbial community assembly in response to warming
Source: Nat Commun. 2020 Sep 18;11:4717. doi: 10.1038/s41467-020-18560-z (PMC7501310; doi:10.1038/s41467-020-18560-z)
Supplement: Supplementary file 5 — Supplementary Code 1 [file 41467_2020_18560_MOESM5_ESM.zip › iCAMP_1.2.4.zip!/iCAMP/html/NRI.p.html]

R: Calculate net relatedness index (NRI) by parallel computing.

|  |  |
| --- | --- |
| NRI.p {iCAMP} | R Documentation |

## Calculate net relatedness index (NRI) by parallel computing.

### Description

Calculate net relatedness index (NRI) or other index of null model significance test based on mean pairwise distance (MPD) by parallel computing, for small and medium size dataset.

### Usage

```
NRI.p(comm, dis, nworker = 4, memo.size.GB = 50,
      weighted = c(TRUE, FALSE), check.name = TRUE,
      rand = 1000, output.MPD = c(FALSE, TRUE), silent = FALSE,
      sig.index=c("SES","NRI","Confidence","RC","all"))
```

### Arguments

|  |  |
| --- | --- |
| `comm` | community data matrix. rownames are sample names. colnames are species names. |
| `dis` | Phylogenetic distance matrix |
| `nworker` | for parallel computing. Either a character vector of host names on which to run the worker copies of R, or a positive integer (in which case that number of copies is run on localhost). default is 4, means 4 threads will be run. |
| `memo.size.GB` | numeric, to set the memory size as you need, so that calculation of large tree will not be limited by physical memory. unit is Gb. default is 50Gb. |
| `weighted` | Logic, consider abundances or not (just presence/absence). default is TRUE. |
| `check.name` | Logic, whether to check the species names in comm and dis. default is TRUE. |
| `rand` | integer, randomization times. default is 1000. |
| `output.MPD` | Logic, whether to output observed MNTD, so that you do not need to calculate observed MNTD alone. default is FALSE. |
| `silent` | Logic, if FALSE, some messages will be showed during calculation. Default is FALSE. |
| `sig.index` | character, the index for null model significance test. SES or NRI, standard effect size, i.e. net relatedness index (NRI); Confidence, percentage of null values less extreme than the observed value, i.e. non-parametric one-side confidence level; RC, modified Raup-Crick index (RC) based on MPD, i.e. count the number of null MPD lower than observed MPD plus a half of the number of null MPD equal to observed MPD, to get alpha, then calculate MPD-based RC as (2 x alpha - 1); all, output all the three indexes. default is SES. If input a vector, only the first element will be used. |

### Details

The net relatedness index (NRI) is a standardized measure of the mean pairwise phylogenetic distance in each sample/community (MPD). Currently this function only performs one null model algorithm, "taxa.labels" ("taxa shuffle", Kembel 2009), which is to shuffle distance matrix labels (across all taxa included in distance matrix). If the randomized results are all the same, the standard deviation will be zero and NRI will be NAN. In this case, NRI will be set as zero, since the observed result is not differentiable from randomized results.

RC (Chase et al 2011) and Confidence (Ning et al 2020) are alternative significance test indexes to evaluate how the observed diversity index deviates from null expectation, which could be a better metric than standardized effect size (NRI) in some cases, e.g. null values do not follow normal distribution.

### Value

Output can be a data.frame with each row representing a sample and only one column of index values, or a list of several data.frame objects.

|  |  |
| --- | --- |
| `SES` | output if sig.index is Confidence or all, a data.frame with NRI value for each sample. |
| `Confidence` | output if sig.index is SES or all, a data.frame showing confidence level based on MPD for each sample. |
| `RC` | output if sig.index is RC or all, a data.frame showing RC based on MPD for each sample. |
| `MPD.obs` | output if output.MPD is TRUE, a data.frame showing observed MPD for each sample. |
| `MPD.rand` | output if output.MPD is TRUE, a matrix showing all null MPD values. |

### Note

Version 2: 2020.8.19, update help document, add example
Version 1: 2017.5.10

### Author(s)

Daliang Ning

### References

Webb CO, Ackerly DD, and Kembel SW. 2008. Phylocom: software for the analysis of phylogenetic community structure and trait evolution. Bioinformatics 18:2098-2100

Kembel, S.W. (2009). Disentangling niche and neutral influences on community assembly: assessing the performance of community phylogenetic structure tests. Ecol Lett, 12, 949-960.

Chase, J.M., Kraft, N.J.B., Smith, K.G., Vellend, M. & Inouye, B.D. (2011). Using null models to disentangle variation in community dissimilarity from variation in alpha-diversity. Ecosphere, 2, 1-11.

Ning, D., Yuan, M., Wu, L., Zhang, Y., Guo, X., Zhou, X. et al. (2020). A quantitative framework reveals the ecological drivers of grassland soil microbial community assembly in response to warming. bioRxiv. doi:10.1101/2020.02.22.960872 (Nature Communications, final revision).

### See Also

`mpdn`

### Examples

```
data("example.data")
comm=example.data$comm
pd=example.data$pd
nworker=2 # parallel computing thread number.
rand.time=20 # usually use 1000 for real data.
sigmpd=NRI.p(comm=comm, dis=pd, nworker=nworker,
             weighted=TRUE, rand=rand.time,
             sig.index="all")
NRI=sigmpd$SES
CMPD=sigmpd$Confidence
RCMPD=sigmpd$RC
```

---

[Package *iCAMP* version 1.2.4 Index]
