# Supplementary material for: A quantitative framework reveals ecological drivers of grassland microbial community assembly in response to warming
Source: Nat Commun. 2020 Sep 18;11:4717. doi: 10.1038/s41467-020-18560-z (PMC7501310; doi:10.1038/s41467-020-18560-z)
Supplement: Supplementary file 5 — Supplementary Code 1 [file 41467_2020_18560_MOESM5_ESM.zip › iCAMP_1.2.4.zip!/iCAMP/html/NTI.p.html]

R: Calculate nearest taxon index (NTI) with parallel computing

|  |  |
| --- | --- |
| NTI.p {iCAMP} | R Documentation |

## Calculate nearest taxon index (NTI) with parallel computing

### Description

Calculate nearest taxon index (NTI) of each sample with parallel computing.

### Usage

```
NTI.p(comm, dis, nworker = 4, memo.size.GB = 50,
      weighted = c(TRUE, FALSE), rand = 1000,
      check.name = TRUE, output.MNTD = c(FALSE, TRUE),
      sig.index=c("SES","NTI","Confidence","RC","all"),
      silent=FALSE)
```

### Arguments

|  |  |
| --- | --- |
| `comm` | community data matrix. rownames are sample names. colnames are species names. |
| `dis` | Phylogenetic distance matrix. |
| `nworker` | for parallel computing. Either a character vector of host names on which to run the worker copies of R, or a positive integer (in which case that number of copies is run on localhost). default is 4, means 4 threads will be run. |
| `memo.size.GB` | numeric, to set the memory size as you need, so that calculation of large tree will not be limited by physical memory. unit is Gb. default is 50Gb. |
| `weighted` | Logic, consider abundances or not (just presence/absence). default is TRUE. |
| `rand` | integer, randomization times. default is 1000. |
| `check.name` | logic, whether to check the taxa names in comm and dis, which must be the same and in the same order; if not match, remove mismatched names and change to the same order. default is TRUE. |
| `output.MNTD` | logic, if TRUE, the NTI and MNTD will be output, if FALSE, only output NTI. |
| `sig.index` | character, the index for null model significance test. SES or NTI, standard effect size, i.e. nearest taxon index (NTI); Confidence, percentage of null values less extreme than the observed value, i.e. non-parametric one-side confidence level; RC, modified Raup-Crick index (RC) based on MNTD, i.e. count the number of null MNTD lower than observed MNTD plus a half of the number of null MNTD equal to observed MNTD, to get alpha, then calculate MNTD-based RC as (2 x alpha - 1); all, output all the three indexes. default is SES. If input a vector, only the first element will be used. |
| `silent` | logic, if FALSE, some messages will show during calculation. |

### Details

The nearest taxon index (NTI) is a standardized measure of the mean phylogenetic distance to the nearest taxon in each sample/community (MNTD). Currently this function only performs one null model algorithm, "taxa.labels" ("taxa shuffle", Kembel 2009), which is to shuffle distance matrix labels (across all taxa included in distance matrix). If the randomized results are all the same, the standard deviation will be zero and NTI will be NAN. In this case, NTI will be set as zero, since the observed result is not differentiable from randomized results.

RC (Chase et al 2011) and Confidence (Ning et al 2020) are alternative significance test indexes to evaluate how the observed diversity index deviates from null expectation, which could be a better metric than standardized effect size (NTI) in some cases, e.g. null values do not follow normal distribution.

### Value

If output.MNTD is FALSE, output is a one-column matrix where rownames are sample IDs and the only column shows NTI values. If output.MNTD is TRUE, output is a list of three elements.

|  |  |
| --- | --- |
| `NTI` | matrix, NTI values. |
| `MNTD` | matrix, observed MNTD. |
| `MNTD.rand` | array, null MNTD values, the third dimension represent randomization times. |

### Note

Version 2: 2020.8.19, update help document, add example.
Version 1: 2018.10.19

### Author(s)

Daliang Ning

### References

Webb CO, Ackerly DD, and Kembel SW. 2008. Phylocom: software for the analysis of phylogenetic community structure and trait evolution. Bioinformatics 18:2098-2100

Kembel, S.W. (2009). Disentangling niche and neutral influences on community assembly: assessing the performance of community phylogenetic structure tests. Ecol Lett, 12, 949-960.

Chase, J.M., Kraft, N.J.B., Smith, K.G., Vellend, M. & Inouye, B.D. (2011). Using null models to disentangle variation in community dissimilarity from variation in alpha-diversity. Ecosphere, 2, 1-11.

Ning, D., Yuan, M., Wu, L., Zhang, Y., Guo, X., Zhou, X. et al. (2020). A quantitative framework reveals the ecological drivers of grassland soil microbial community assembly in response to warming. bioRxiv. doi:10.1101/2020.02.22.960872 (Nature Communications, final revision).

### See Also

`mntdn`

### Examples

```
data("example.data")
comm=example.data$comm
pd=example.data$pd
nworker=2 # parallel computing thread number.
rand.time=4 # usually use 1000 for real data.
sigmntd=NTI.p(comm=comm, dis=pd, nworker = nworker,
              weighted = TRUE, rand = rand.time,
              sig.index="all")
NTI=sigmntd$SES
CMNTD=sigmntd$Confidence
RCMNTD=sigmntd$RC
```

---

[Package *iCAMP* version 1.2.4 Index]
