# Supplementary material for: A quantitative framework reveals ecological drivers of grassland microbial community assembly in response to warming
Source: Nat Commun. 2020 Sep 18;11:4717. doi: 10.1038/s41467-020-18560-z (PMC7501310; doi:10.1038/s41467-020-18560-z)
Supplement: Supplementary file 5 — Supplementary Code 1 [file 41467_2020_18560_MOESM5_ESM.zip › iCAMP_1.2.4.zip!/iCAMP/html/null.norm.html]

R: Normality test for null values

|  |  |
| --- | --- |
| null.norm {iCAMP} | R Documentation |

## Normality test for null values

### Description

To test whether the null values of each turnover of each bin follow normal distribution.

### Usage

```
null.norm(icamp.output = NULL, rand.list = NULL, index.name = "Test.Index",
          p.norm.cut = 0.05, detail.out = FALSE)
```

### Arguments

|  |  |
| --- | --- |
| `icamp.output` | list, the exact output of the function icamp.big in which detail.null must be TRUE, to save all null values. |
| `rand.list` | list, the null values of a certain dissimilarity index. Each eletment is a matrix that represents a bin. In each eletment matrix, the first two columns indicate sample IDs of the pairwise comparison (turnover), and each of the other columns shows the null values from one time of randomization. |
| `index.name` | character, when rand.list is given, to specify the name of the dissimilarity index. |
| `p.norm.cut` | numeric, the threshold of significant P value. A p value lower than this indicates significant difference from normal distribution. |
| `detail.out` | logic, if TRUE, the detailed statistics and P values for each turnover of each bin will be output; otherwise, only output a summary on non-normal percentage for each bin. |

### Details

Normal distribution of null values is basic assumption when using Standard Effect Size (SES, e.g. betaNRI, betaNTI) to identify significant difference between null and observed values. This function uses five different methods to perform normality test, including Anderson-Darling test (Anderson), Cramer-von Mises test (Cramer), Kolmogorov-Smirnov test (Kolmogorov, also known as Lilliefors test), Shapiro-Francia test (ShapiroF), and Shapiro-Wilk test (Shapiro). The function 'shapiro.test' in package 'stats', and various functions in package 'nortest' are used.

### Value

Output is a list object.

|  |  |
| --- | --- |
| `summary` | data.frame, each row represents a bin and a dissimilarity index. Seven columns. The first column indicates the dissimilarity index; the second column indicate Bin ID; each of the other columns indicate non-normal ratio based on a method. The non-normal raio is calculated as percentage of turnovers where null value distribution is significantly different from normal distribution. |
| `P.value.cut` | the value of p.norm.cut |
| `detail` | list, each first-level element represents a dissimilarity index; each second-level element is a matrix represents a bin; and the matrix has 14 columns, including the dissimilarity index (Index), bin ID (BinID), sample IDs (name1 and name2), and the statistics and P value based on different methods. |

### Note

Version 2: 2020.8.19, update help document, add example
Version 1: 2020.8.1

### Author(s)

Daliang Ning

### References

Stephens, M.A. (1986): Tests based on EDF statistics. In: D'Agostino, R.B. and Stephens, M.A., eds.: Goodness-of-Fit Techniques. Marcel Dekker, New York.

Dallal, G.E. and Wilkinson, L. (1986): An analytic approximation to the distribution of Lilliefors' test for normality. The American Statistician, 40, 294-296.

Stephens, M.A. (1974): EDF statistics for goodness of fit and some comparisons. Journal of the American Statistical Association, 69, 730-737.

Royston, P. (1993): A pocket-calculator algorithm for the Shapiro-Francia test for non-normality: an application to medicine. Statistics in Medicine, 12, 181-184.

Thode Jr., H.C. (2002): Testing for Normality. Marcel Dekker, New York

Patrick Royston (1982). An extension of Shapiro and Wilk's W test for normality to large samples. Applied Statistics, 31, 115-124. doi: 10.2307/2347973.

Patrick Royston (1982). Algorithm AS 181: The W test for Normality. Applied Statistics, 31, 176-180. doi: 10.2307/2347986.

Patrick Royston (1995). Remark AS R94: A remark on Algorithm AS 181: The W test for normality. Applied Statistics, 44, 547-551. doi: 10.2307/2986146.

Juergen Gross and Uwe Ligges (2015). nortest: Tests for Normality. R package version 1.0-4. https://CRAN.R-project.org/package=nortest

### See Also

`icamp.big`, `change.sigindex`

### Examples

```
data("icamp.out")
nntest=null.norm(icamp.output = icamp.out, detail.out = TRUE)
```

---

[Package *iCAMP* version 1.2.4 Index]
