# Supplementary material for: A quantitative framework reveals ecological drivers of grassland microbial community assembly in response to warming
Source: Nat Commun. 2020 Sep 18;11:4717. doi: 10.1038/s41467-020-18560-z (PMC7501310; doi:10.1038/s41467-020-18560-z)
Supplement: Supplementary file 5 — Supplementary Code 1 [file 41467_2020_18560_MOESM5_ESM.zip › iCAMP_1.2.4.zip!/iCAMP/html/pdist.big.html]

R: Pairwise phylogenetic distance matrix from big tree

|  |  |
| --- | --- |
| pdist.big {iCAMP} | R Documentation |

## Pairwise phylogenetic distance matrix from big tree

### Description

Calculates between-species phylogenetic distance matrix from a tree, using bigmemory to deal with too large dataset.

### Usage

```
pdist.big(tree, wd = getwd(), tree.asbig = FALSE, output = FALSE,
          nworker = 4, nworker.pd = nworker, memory.G = 50, time.count = FALSE)
```

### Arguments

|  |  |
| --- | --- |
| `tree` | phylogenetic tree, an object of class "phylo". |
| `wd` | path of a folder to save the big phylogenetic distance matrix, default is current work directory. |
| `tree.asbig` | logic, whether to treat tree attributes also as big data, default is FALSE, generally no need to set as TRUE. |
| `output` | logic, whether to output the big phylogenetic distance matrix, default is FALSE, generally do not output it, could be too large. |
| `nworker` | for parallel computing the tree paths. a positive integer (in which case that number of copies is run on localhost). default is 4, means 4 threads will be run. |
| `nworker.pd` | for parallel computing the phylogenetic distance matrix. default is set the same as nworker. may need to set lower than nworker if the matrix is too large. |
| `memory.G` | numeric, to set the memory size as you need, so that calculation of large tree will not be limited by physical memory. unit is Gb. default is 50Gb |
| `time.count` | logic, whether to count calculation time, default is FALSE. |

### Details

The cophenetic distance between each pair of taxa is calculated (Sokal and Rohlf 1962). Modified from the function "cophenetic" in package "ape" (Paradis & Schliep 2018), this function can calculate pairwise distance from large phylogenetic tree quickly by parallel computing. This function uses bigmemory (Kane et al 2013) to deal with large phylogenetic distance matrix, which will not occupy memory but directly be saved at the hard disk.

### Value

Output is a list

|  |  |
| --- | --- |
| `tip.label` | OTU ids or species names, which is tip.label in tree file. |
| `pd.wd` | the folder saving the big phylogenetic distance matrix. |
| `pd.file` | the folder saving the big phylogenetic distance matrix. |
| `pd.name.file` | the file saving the tip.label information. |

### Note

Version 3: 2020.8.19, add example.
Version 2: 2017.3.13
Version 1: 2015.7.24

### Author(s)

Daliang Ning

### References

Sokal, R. R. & Rohlf, F. J.. (1962). The comparison of dendrograms by objective methods. Taxon, 11:33-40

Paradis, E. & Schliep, K. (2018). ape 5.0: an environment for modern phylogenetics and evolutionary analyses in R. Bioinformatics 35: 526-528.

Kane, M.J., Emerson, J., & Weston, S. (2013). Scalable Strategies for Computing with Massive Data. Journal of Statistical Software, 55(14), 1-19. URL http://www.jstatsoft.org/v55/i14/.

### Examples

```
data("example.data")
tree=example.data$tree
# since pdist.big need to save output to a certain folder,
# the following code is set as 'not run'.
# but you may test the code on your computer
# after change the folder path for 'save.wd'.
## Not run: 
  save.wd="C:/test/" # please change to the folder you want to save the pd.big output.
  nworker=2 # parallel computing thread number
  pd.big=pdist.big(tree = tree, wd=save.wd, nworker = nworker)

## End(Not run)
```

---

[Package *iCAMP* version 1.2.4 Index]
