# Supplementary material for: A quantitative framework reveals ecological drivers of grassland microbial community assembly in response to warming
Source: Nat Commun. 2020 Sep 18;11:4717. doi: 10.1038/s41467-020-18560-z (PMC7501310; doi:10.1038/s41467-020-18560-z)
Supplement: Supplementary file 5 — Supplementary Code 1 [file 41467_2020_18560_MOESM5_ESM.zip › iCAMP_1.2.4.zip!/iCAMP/html/ps.bin.html]

R: Test within-bin phylogenetic signal

|  |  |
| --- | --- |
| ps.bin {iCAMP} | R Documentation |

## Test within-bin phylogenetic signal

### Description

Use Mantel test to evaluate phylogenetic signal within each bin, i.e. correlation between phylogenetic distance and niche difference.

### Usage

```
ps.bin(sp.bin, sp.ra, spname.use = NULL,
       pd.desc = "pd.desc", pd.spname, pd.wd,
       nd.list, nd.spname = NULL, ndbig.wd = NULL,
       cor.method = c("pearson", "spearman"),
       r.cut = 0.01, p.cut = 0.2, min.spn = 6)
```

### Arguments

|  |  |
| --- | --- |
| `sp.bin` | one-column matrix or data.frame, indicating the bin ID for each species (OTU or ASV), rownames are species IDs. usually use the third column of "sp.bin" in the output of `taxa.binphy.big`. if input matrix with multiple columns, only the first column will be used. |
| `sp.ra` | one-column matrix or data.frame, or a vector with name for each element, indicating mean relative abundance of each species. |
| `spname.use` | character vector, to specify which species will be used for phylogenetic signal test. Default is NULL, means to use all species. |
| `pd.desc` | the name of the file to hold the backingfile description of the phylogenetic distance matrix, it is usually "pd.desc" if using default setting in pdist.big function. |
| `pd.spname` | character vector, species id in the same order as the big matrix of phylogenetic distances. |
| `pd.wd` | folder path, where the bigmemmory file of the phylogenetic distance matrix are saved. |
| `nd.list` | list object. if the niche difference matrixes are big.matrix, each element of this list is the big.matrix backingfile description, e.g. "pH.ND.desc"; otherwise, each element is a niche difference matrix based on an environment factor. usually this is the "nd" in the output of `dniche`. |
| `nd.spname` | character vector or NULL. If the niche difference matrixes are big.matrix, this is the species IDs in the same order as in each big matrix; otherwise, this should be set as NULL, the species IDs will be extracted from nd.list. |
| `ndbig.wd` | folder path or NULL. If the niche difference matrixes are big.matrix, this is where the big matrixes of niche differences are saved; otherwise, this is NULL. |
| `cor.method` | Correlation method, as accepted by cor: "pearson", "spearman" or "kendall". Multiple methods at a time are allowed. |
| `r.cut` | the cutoff of correlaiton coefficient to identify significant correlation. |
| `p.cut` | the cutoff of p value to identify significant correlation. |
| `min.spn` | the minimal spcies (or OUT or ASV) number required for phylogenetic signal test. |

### Details

This is simply Mantel test between phylogenetic distance and niche difference (i.e. phylogenetic signal) within each bin. Then, it returns the overall relative abundance of bins with significant phylogenetic signal, average correlation coefficient, as well as detailed results in each bin, to evaluate within-bin phylogenetic signal of the binning (inputed as sp.bin). Bigmemory (Kane et al 2013) is used to deal with large datasets.

### Value

Output is a list object with two elements.

|  |  |
| --- | --- |
| `Index` | Summary of phylogenetic signal test. The indexes include relative abundance of bins with significant phylogenetic signal in all bins (RAsig) or in bins with species number larger than min.spn (RAsig.adj), average correlation coefficient in significant bins (MeanR.sig) or in all bins (MeanR). |
| `detail` | correlation coefficient (r) and p value in each bin. |

### Note

Version 2: 2020.8.18, update help document, add example.
Version 1: 2020.5.15

### Author(s)

Daliang Ning

### References

Ning, D., Yuan, M., Wu, L., Zhang, Y., Guo, X., Zhou, X. et al. (2020). A quantitative framework reveals the ecological drivers of grassland soil microbial community assembly in response to warming. bioRxiv. doi:10.1101/2020.02.22.960872 (Nature Communications, final revision).

Kane, M.J., Emerson, J., & Weston, S. (2013). Scalable Strategies for Computing with Massive Data. Journal of Statistical Software, 55(14), 1-19. URL http://www.jstatsoft.org/v55/i14/.

### See Also

`taxa.binphy.big`, `dniche`

### Examples

```
data("example.data")
comm=example.data$comm
env=example.data$env
tree=example.data$tree

# since big.memory need to specify a certain folder,
# the following code is set as 'not run'.
# but you may test the code on your computer
# after change the folder path for 'save.wd'.
## Not run: 
  save.wd="C:/test/" # please change to the folder you want to save the big niche difference matrix.
  nworker=2 # parallel computing thread number
  niche.dif=dniche(env = env, comm = comm,
                   method = "niche.value", nworker = nworker,
                   out.dist=FALSE,bigmemo=TRUE,nd.wd = save.wd)
  pd.big=pdist.big(tree = tree, wd=save.wd, nworker = nworker)
  
  ds = 0.2 # setting can be changed to explore the best choice
  bin.size.limit = 5 # setting can be changed to explore the best choice.
  # here, bin.size.limit is set as 5 just for the small example dataset.
  # For real data, usually try 12 to 48.
  
  phylobin=taxa.binphy.big(tree = tree, pd.desc = pd.big$pd.file,
                           pd.spname = pd.big$tip.label, pd.wd = pd.big$pd.wd,
                           ds = ds, bin.size.limit = bin.size.limit,
                           nworker = nworker)
  sp.bin=phylobin$sp.bin[,3,drop=FALSE]
  
  sp.ra=colMeans(comm/rowSums(comm))
  abcut=3 # you may remove some species, if they are too rare to perform reliable correlation test.
  commc=comm[,colSums(comm)>=abcut,drop=FALSE]
  dim(commc)
  spname.use=colnames(commc)
  
  binps=ps.bin(sp.bin = sp.bin,sp.ra = sp.ra,spname.use = spname.use,
               pd.desc = pd.big$pd.file, pd.spname = pd.big$tip.label,
               pd.wd = pd.big$pd.wd, nd.list = niche.dif$nd,
               nd.spname = niche.dif$names, ndbig.wd = niche.dif$nd.wd,
               cor.method = "pearson",r.cut = 0.1, p.cut = 0.05, min.spn = 5)

## End(Not run)
```

---

[Package *iCAMP* version 1.2.4 Index]
