# Supplementary material for: A quantitative framework reveals ecological drivers of grassland microbial community assembly in response to warming
Source: Nat Commun. 2020 Sep 18;11:4717. doi: 10.1038/s41467-020-18560-z (PMC7501310; doi:10.1038/s41467-020-18560-z)
Supplement: Supplementary file 5 — Supplementary Code 1 [file 41467_2020_18560_MOESM5_ESM.zip › iCAMP_1.2.4.zip!/iCAMP/html/qp.bin.js.html]

R: Calculate relative importance of community assembly processes

|  |  |
| --- | --- |
| qp.bin.js {iCAMP} | R Documentation |

## Calculate relative importance of community assembly processes

### Description

Identify the community assembly process governing each bin in each turnover (i.e. pairwise comparison between two samples/communities), then calculate the relative importance of community assembly processes in each turnover.

### Usage

```
qp.bin.js(sig.phy.bin=NULL, sig.phy2.bin=NULL, sig.tax.bin=NULL,
          bin.weight, sig.phy.cut=1.96, sig.phy2.cut=1.96,
          sig.tax.cut=0.95, check.name=FALSE)
```

### Arguments

|  |  |
| --- | --- |
| `sig.phy.bin` | matrix, the first two columns are sample IDs, thus each row represent a turnover between two samples. From the third column, each column shows the significance testing index of phylogenetic null model analysis (e.g. betaNTI) of a bin. NULL means the data is not available. |
| `sig.phy2.bin` | matrix, the same as sig.phy.bin, serves as the second phylogenetic metrics when phylogenetic null model test is based on two different beta diversity indexes, e.g. both betaMPD and betaMNTD. NULL means the data is not available. |
| `sig.tax.bin` | matrix, the first two columns are sample IDs, thus each row represent a turnover between two samples. From the third column, each column shows the significance testing index of taxonomic null model analysis (e.g. RC.Bray) of a bin. NULL means the data is not available. |
| `bin.weight` | matrix, the first two columns are sample IDs, thus each row represent a turnover between two samples. From the third column, each column shows the abundance sum of a bin in each pair of samples. |
| `sig.phy.cut` | numeric, a cutoff for the null model significance testing index based on a phylogenetic beta diversity index, e.g. betaNRI based on betaMPD, default is 1.96. |
| `sig.phy2.cut` | numeric, a cutoff for the null model significance testing index based on the second phylogenetic beta diversity index, e.g. betaNTI based on betaMNTD, default is 1.96. |
| `sig.tax.cut` | numeric, a cutoff for the null model significance testing index based on a taxonomic beta diversity index, e.g. RC based on Bray-Curtis, default is 0.95. |
| `check.name` | logic, whether to check the sample IDs in different input matrixess are in the same order. |

### Details

The framework is proposed by James Stegen (2013 and 2015), to identify governing ecologcial process based on phylogenetic (betaNTI) and taonomic (RC.Bray) null model analysis. In all pairwised comparisons between samples/communities, the non-random phylogenetic turnovers recognized by phylogeny shuffle were counted as influence of environment selection, and the non-random taxonomic turnovers in the rest pairwised comparisons were counted as influence of dispersal limitation or homogenizing dispersal. The rest part is called undominated.

This function applied this framework to each phylogenetic bin and allowed to use betaNRI and/or betaNTI. When both betaNTI and betaNRI are provided, a turnover is idientified as controlled by selection when either betaNRI or betaNTI is significant. Alternatively, RC or confidence level based on betaMPD and/or betaMNTD can also be used (Ning et al. 2020).

### Value

Output is a matrix. The first two columns are sample IDs, and each row represent a turnover between two samples. From the third column, each column shows the relative importance of a community assembly process in each turnover (pairwise comparison between each pair of samples).

### Note

Version 5: 2020.8.19, update help document, add example.
Version 4: 2020.7.28, change bNTI.bin=NULL,bNRI.bin=NULL,RC.bin=NULL to sig.phy.bin and sig.tax.bin.
Version 3: 2018.10.20, add bNRI.bin as option; add check.name.
Version 2: 2016.3.26, add RC.all option
Version 1: 2015.12.16

### Author(s)

Daliang Ning

### References

Ning, D., Yuan, M., Wu, L., Zhang, Y., Guo, X., Zhou, X. et al. (2020). A quantitative framework reveals the ecological drivers of grassland soil microbial community assembly in response to warming. bioRxiv. doi:10.1101/2020.02.22.960872 (Nature Communications, final revision).

Stegen JC, Lin X, Fredrickson JK, Chen X, Kennedy DW, Murray CJ et al. 2013. Quantifying community assembly processes and identifying features that impose them. Isme Journal 7: 2069-2079.

Stegen JC, Lin X, Fredrickson JK, Konopka AE. 2015. Estimating and mapping ecological processes influencing microbial community assembly. Frontiers in Microbiology 6.

### See Also

`icamp.big`

### Examples

```
data("icamp.out")
bNRIbins=icamp.out$detail$SigbMPDi
RCbins=icamp.out$detail$SigBCa
binwt=icamp.out$detail$bin.weight
qpbin=qp.bin.js(sig.phy.bin = bNRIbins,sig.tax.bin = RCbins,
                bin.weight = binwt, sig.phy.cut = 1.96,
                sig.tax.cut = 0.95, check.name = TRUE)
```

---

[Package *iCAMP* version 1.2.4 Index]
