# Supplementary material for: A quantitative framework reveals ecological drivers of grassland microbial community assembly in response to warming
Source: Nat Commun. 2020 Sep 18;11:4717. doi: 10.1038/s41467-020-18560-z (PMC7501310; doi:10.1038/s41467-020-18560-z)
Supplement: Supplementary file 5 — Supplementary Code 1 [file 41467_2020_18560_MOESM5_ESM.zip › iCAMP_1.2.4.zip!/iCAMP/html/qpen.html]

R: Quantifying assembly processes based on entire-community null...

|  |  |
| --- | --- |
| qpen {iCAMP} | R Documentation |

## Quantifying assembly processes based on entire-community null model analysis

### Description

The previous framework quantifying assembly processes based on entire-community null model analysis (Stegen et al 2013, 2015). add bigmemory function to handle large datasets.

### Usage

```
qpen(comm = NULL, pd = NULL, pd.big.wd = NULL,
     pd.big.spname = NULL, tree = NULL,
     bNTI = NULL, RC = NULL, ab.weight = TRUE,
     meta.ab = NULL, exclude.conspecifics = FALSE,
     rand.time = 1000, sig.bNTI = 1.96, sig.rc = 0.95,
     nworker = 4, memory.G = 50, project = NA,
     wd = getwd(), output.detail = FALSE, save.bNTIRC = FALSE)
```

### Arguments

|  |  |
| --- | --- |
| `comm` | matrix or data.frame, community data, each row is a sample or site, each colname is a taxon (species or OTU or ASV), thus rownames should be sample IDs, colnames should be taxa IDs. |
| `pd` | a character or a matrix or NULL. If it is a character, it specifies the name of the file to hold the backingfile description of the big phylogenetic distance matrix, it is usually "pd.desc" if using default setting in pdist.big function. If it is matrix, it is the phylogenetic distance matrix with taxa IDs as colnames and rownames. The function will check the consistency of species names between phylogenetic matrix and comm. if pd is NULL, the function will calculate phylogenetic distance matrix from tree. |
| `pd.big.wd` | a folder path, where the bigmemmory file of the phylogenetic distance matrix are saved. If pd has given the phylogenetic matrix, pd.big.wd should be NULL. If pd is NULL and pd.big.wd is NULL either, a folder will be created to save the big phylgoenetic distance matrix. |
| `pd.big.spname` | character vector, taxa id in the same rank as the big matrix of phylogenetic distances. not necessary if pd has given the phylogenetic matrix. |
| `tree` | phylogenetic tree, an object of class "phylo". |
| `bNTI` | a matrix, if beta nearest taxon index (betaNTI) values are available, just directly input them as a squre matrix here. |
| `RC` | a matrix, if modified Raup-Crick metric based Bray-Curtis (RCbray) values are available, just directly input them as a squre matrix here. |
| `ab.weight` | logic, abundance-weighted or binary. default is TRUE, means abundance-weighted. |
| `meta.ab` | a numeric vector, to define the relative aubndance of each species in the regional pool. Default setting is NULL, means to calculate meta.ab as average relative abundance of each species across the samples. |
| `exclude.conspecifics` | Logic, should conspecific taxa in different communities be exclude from MNTD calculations? default is FALSE. The same as in the function bmntd. |
| `rand.time` | integer, randomization times. default is 1000. |
| `sig.bNTI` | numeric, the cutoff of significant betaNTI, default is 1.96. |
| `sig.rc` | numeric, the cutoff of significant modified Raup-Crick metric, default is 0.95. |
| `nworker` | for parallel computing. Either a character vector of host names on which to run the worker copies of R, or a positive integer (in which case that number of copies is run on localhost). default is 4, means 4 threads will be run. |
| `memory.G` | numeric, to set the memory size as you need, so that calculation of large tree will not be limited by physical memory. unit is Gb. default is 50Gb. |
| `project` | character string, the prefix of saved output files. |
| `wd` | a folder path, where the files will be saved. |
| `output.detail` | logic, if TRUE, some detailed results including all null values will be output. |
| `save.bNTIRC` | logic, if TRUE, a file will be saved in the folder specified by wd. |

### Details

This method is developed by Dr. James Stegen (2013 and 2015), from which we developed iCAMP. In all pairwised comparisons between samples/communities, the non-random phylogenetic turnovers recognized by phylogeny shuffle were counted as influence of environment selection (betaNTI>1.96 or <-1.96), and the non-random taxonomic turnovers in the rest pairwised comparisons were counted as influence of dispersal limitation (RCbray>0.95) or homogenizing dispersal (RCbray<-0.95). The rest part is called undominated. Please read the references for details.

betaNTI is the standardized effect size between the observed and null values of beta mean nearest taxon distance (betaMNTD). RCbray is the modified Roup-Crick metric based on taxonomic Bray-Curtis dissimilarity index.

Bigmemory (Kane et al 2013) is used to deal with large datasets.

### Value

output is a list with following elements.

|  |  |
| --- | --- |
| `ratio` | The overall percentage of turnovers governed by each process |
| `result` | a matrix, showing betaMNTD, Bray-Curtis dissimilarity, betaNTI, RC, and the identified process governing each turnover. Each row represents a turnover between two samples. |
| `pd` | phylogenetic distance matrix or the backingfile name if phylogenetic distance is saved by bigmemeory function. |
| `bMNTD` | a square matrix of observed betaMNTD. |
| `BC` | a square matrix of observed Bary-Curtis index. |
| `bMNTD.rand` | a matrix showing all null values of betaMNTD. |
| `BC.rand` | a matrix showing all null values of Bray-Curtis index. |
| `setting` | a data.frame showing all settings for this function. |

### Note

Version 4: 2020.8.21, update help document, add example.
Version 3: 2016.2.15, add options to use bigmemory to handle large phylogenetic distance matrixes.
Version 2: 2015.11.22

### Author(s)

Daliang Ning

### References

Stegen, J.C., Lin, X., Fredrickson, J.K., Chen, X., Kennedy, D.W., Murray, C.J. et al. (2013). Quantifying community assembly processes and identifying features that impose them. ISME J, 7, 2069.

Stegen, J.C., Lin, X., Fredrickson, J.K. & Konopka, A.E. (2015). Estimating and mapping ecological processes influencing microbial community assembly. Front Microbiol, 6, 370.

Kane, M.J., Emerson, J., & Weston, S. (2013). Scalable Strategies for Computing with Massive Data. Journal of Statistical Software, 55(14), 1-19. URL http://www.jstatsoft.org/v55/i14/.

### See Also

`bNTIn.p`, `bNTI.bin.big`, `RC.pc`

### Examples

```
data("example.data")
comm=example.data$comm
pd=example.data$pd
nworker=2 # parallel computing thread number
rand.time=10 # usually use 1000 for real data.
qp=qpen(comm=comm, pd=pd,
        rand.time=rand.time,nworker=nworker)
```

---

[Package *iCAMP* version 1.2.4 Index]
