# Supplementary material for: A quantitative framework reveals ecological drivers of grassland microbial community assembly in response to warming
Source: Nat Commun. 2020 Sep 18;11:4717. doi: 10.1038/s41467-020-18560-z (PMC7501310; doi:10.1038/s41467-020-18560-z)
Supplement: Supplementary file 5 — Supplementary Code 1 [file 41467_2020_18560_MOESM5_ESM.zip › iCAMP_1.2.4.zip!/iCAMP/html/RC.pc.html]

R: Modified Raup-Crick index based on Bray-Curtis similarity

|  |  |
| --- | --- |
| RC.pc {iCAMP} | R Documentation |

## Modified Raup-Crick index based on Bray-Curtis similarity

### Description

The Raup-Crick based on taxonomic dissimilarity index (i.e. Bray-Curtis) is to use null models to disentangle variation in community dissimilarity from variation in alpha-diversity.

### Usage

```
RC.pc(comm, rand = 1000, na.zero = TRUE, nworker = 4,
      memory.G = 50, weighted = TRUE, unit.sum = NULL,
      meta.ab = NULL,sig.index=c("RC","Confidence","SES"),
      detail.null=FALSE,output.bray=FALSE,silent=FALSE)
```

### Arguments

|  |  |
| --- | --- |
| `comm` | Community data matrix. rownames are sample names. colnames are species names. |
| `rand` | integer, randomization times, default is 1000. |
| `na.zero` | logic. If community data marix has any zero-sum row (sample), Bray-Curtis index will be NA. Somtimes, this kind of NA need be set as zero to avoid some format problem in following calculation. Default is TRUE. |
| `nworker` | for parallel computing. Either a character vector of host names on which to run the worker copies of R, or a positive integer (in which case that number of copies is run on localhost). default is 4, means 4 threads will be run. |
| `memory.G` | numeric, to set the memory size as you need, so that calculation of big data will not be limited by physical memory. unit is Gb. default is 50Gb. |
| `weighted` | lOgic, whether to use abundance-weighted metrics. default is TRUE |
| `unit.sum` | If unit.sum is set as a number or a numeric vector, the taxa abundances will be divided by unit.sum to calculate the relative abundances, and the Bray-Cuits index in each bin will become manhattan index divided by 2. usually, unit.sum can be set as the sequencing depth in each sample. Default setting is NULL, means not to do this special transformation. |
| `meta.ab` | a numeric vector, to define the relative aubndance of each species in the regional pool. Default setting is NULL, means to calculate meta.ab as average relative abundance of each species across the samples. |
| `sig.index` | character, the index for null model significance test. RC, modified Raup-Crick index (RC) based on Bray-Curtis (BC), i.e. count the number of null BC lower than observed BC plus a half of the number of null BC equal to observed BC, to get alpha, then calculate RCbray as (2 x alpha - 1). SES, standard effect size; Confidence, percentage of null values less extreme than the observed value, i.e. non-parametric one-side confidence level. default is RC. If input a vector, only the first element will be used. |
| `detail.null` | logic, if TRUE, the output will include all the null values. Default is FALSE. |
| `output.bray` | logic, if TRUE, the output will include observed Bray-Curtis. |
| `silent` | logic, if FALSE, some messages will show during calculation. |

### Details

The Raup-Crick based on taxonomic dissimilarity index was proposed by Chase in 2011, and then modified to include consider species relative abundances by Stegen in 2013. The non-random part recognized by RC can reflect the influence niche seletion and extreme dispersal. The original codes used a relatively time-consuming looping. This function improved the efficiency and added some parameters to fit iCAMP analysis.

SES (Kraft et al 2011) and Confidence (Ning et al 2020) are alternative significance testing indexes to evaluate how the observed beta diversity index deviates from null expectation.

### Value

Output is a list.

|  |  |
| --- | --- |
| `index` | a square matrix of RC (or SES or Confidence based on Bray-Curtis) values. |
| `sig.index` | character, indicates the index for null model significance test, RC, Confidence, or SES. |
| `BC.obs` | Output only if output.bray is TRUE. A square matrix of observed Bray-Curtis index values. |
| `rand` | Output only if detail.null is TRUE. A matrix with all null values of Bray-Curtis index for each turnover. |

### Note

Version 6: 2020.8.19, update help document, add example.
Version 5: 2020.8.2, add sig.index, detail.null, and output.bray.
Version 4: 2020.6.14, add meta.ab
Version 3: 2018.10.3, add unit.sum.
Version 2: 2015.8.5, revise the randomization algorithm according to Stegen et al 2013.
Version 1: 2015.2.12

### Author(s)

Daliang Ning

### References

Ning, D., Yuan, M., Wu, L., Zhang, Y., Guo, X., Zhou, X. et al. (2020). A quantitative framework reveals the ecological drivers of grassland soil microbial community assembly in response to warming. bioRxiv. doi:10.1101/2020.02.22.960872 (Nature Communications, final revision).

Chase, J.M., Kraft, N.J.B., Smith, K.G., Vellend, M. & Inouye, B.D. (2011). Using null models to disentangle variation in community dissimilarity from variation in alpha-diversity. Ecosphere, 2, 1-11.

Stegen, J.C., Lin, X., Fredrickson, J.K., Chen, X., Kennedy, D.W., Murray, C.J. et al. (2013). Quantifying community assembly processes and identifying features that impose them. ISME J, 7, 2069.

Kraft, N.J.B., Comita, L.S., Chase, J.M., Sanders, N.J., Swenson, N.G., Crist, T.O. et al. (2011). Disentangling the drivers of beta diversity along latitudinal and elevational gradients. Science, 333, 1755-1758.

### See Also

`RC.bin.bigc`

### Examples

```
data("example.data")
comm=example.data$comm
rand.time=20 # usually use 1000 for real data.
nworker=2 # parallel computing thread number
RC=RC.pc(comm=comm, rand = rand.time,
         nworker = nworker, weighted = TRUE,
         sig.index="RC")
```

---

[Package *iCAMP* version 1.2.4 Index]
