# Supplementary material for: A quantitative framework reveals ecological drivers of grassland microbial community assembly in response to warming
Source: Nat Commun. 2020 Sep 18;11:4717. doi: 10.1038/s41467-020-18560-z (PMC7501310; doi:10.1038/s41467-020-18560-z)
Supplement: Supplementary file 5 — Supplementary Code 1 [file 41467_2020_18560_MOESM5_ESM.zip › iCAMP_1.2.4.zip!/iCAMP/html/snm.html]

R: Estimation of neutral taxa percentae and dispersal rate

|  |  |
| --- | --- |
| snm {iCAMP} | R Documentation |

## Estimation of neutral taxa percentae and dispersal rate

### Description

To calculate the abundance-weighted or unweighted percentage of taxa following Sloan's neutral theory model. The original R code is from Burns et al (2016). Bootstrapping test and different metacommunity settings are added.

### Usage

```
snm(comm, meta.com = NULL, taxon = NULL,
    alpha = 0.05, simplify = FALSE)
snm.boot(comm, rand=1000, meta.com=NULL,
         taxon=NULL, alpha=0.05, detail=TRUE)
snm.comm(comm, treat=NULL, meta.coms=NULL,
         meta.com=NULL, meta.group=NULL,
         rand=1000,taxon=NULL,alpha=0.05,
         two.tail=TRUE,output.detail=TRUE)
```

### Arguments

|  |  |
| --- | --- |
| `comm` | matrix or data.frame, community data, each row is a sample or site, each colname is a taxon (a species or OTU or ASV), thus rownames should be sample IDs, colnames should be taxa IDs. |
| `meta.com` | matrix or data.frame, metacommunity data, each column represents a taxon, can be one or multiple rows. If NULL, the comm will be used to estimate relative abundance of each taxon in the metacommunity. In function snm.comm, if this is given, meta.group will be ignored. |
| `taxon` | matrix or data.frame, classification information of each taxon. Each row represents a taxon. Rownames are taxa IDs. |
| `alpha` | numeric, the significance level threshold counted as alpha value, usually 0.05. |
| `simplify` | logic, if FALSE, the function snm will performan more model fitting test and return detailed statistical information. |
| `rand` | integer, randomization times. default is 1000. |
| `detail` | logic, if TRUE, the detailed output from the function snm will be included into the output of snm.boot. |
| `treat` | matrix or data.frame, indicating the group or treatment of each sample, rownames are sample IDs. if input multiple columns, they will be analyzed one column after another. |
| `meta.coms` | a list, to specify the metacommunity data for each level of treatments in each of the columns of 'treat'. A basic element is a matrix, each column represents a taxon, can be one or multiple rows. If this is given, 'meta.group' and 'meta.com' will be ignored. |
| `meta.group` | a matrix, to specify the metacommunity ID that each sample belongs to. It should have the some column number as 'treat' if 'treat' is given. If meta.coms, meta.com, and meta.group are all NULL, the samples are deemed from the same metacommunity. |
| `two.tail` | logic, to specify the p value is calculated as two-tail (TRUE) or one-tail (FALSE). |
| `output.detail` | logic, if TRUE, the output of the function snm.comm will include all bootstrapping values. |

### Details

The method is developed by Burns et al (2016) based on the Sloan's model (Sloan et al 2006, 2007) which is derived from Hubbell's unified neutral theory (Hubbell 2001). According to neutral theory, the regional relative abundance and occurrence frequency of each taxon should follow a certain model (Sloan et al 2006). Thus, a taxon can be counted as 'neutral taxon' if inside a certain confidence interval of the neutral expectation, and their percentage in a sample may be used to reflect the importance of neutral processes (Burns et al 2016).

### Value

Output of snm is a list.

|  |  |
| --- | --- |
| `stats` | output only if simplify is FALSE, showing statistics about model fitting and coefficients.  m, dispersal rate estimated by Non-linear least squares (NLS).  m.ci.2.5 and m.ci.97.5, the confidence interval of m.  m.mle, dispersal rate calculated by Maximum likelihood estimation.  maxLL, binoLL, and poisLL, maximum likelihood function value (L) for neutral theory model, binomial model, and Poisson model, respectively.  Rsqr, Rsqr.bino, and Rsqr.pois, R squared (coefficient of determination) of neutral theory model, binomial model, and Poisson model, respectively.  RMSE, RMSE.bino, and RMSE.pois, root-mean-square error.  AIC, BIC, AIC.bino, BIC.bino, AIC.pois, and BIC.pois, AIC and BIC of different models.  N, mean individual number in each local community.  Samples, sample number.  Richness, total number of taxa.  Detect, detected limitation of relative abundance in each local community, i.e. 1/N. |
| `detail` | output only if simplify is FALSE. a matrix, showing detailed information of each taxon. Each row represent a taxon. Columns as blow.  p, observed regional relative abundance of each taxon.  freq, observed occurrence frequency of each taxon.  freq.pred, occurrence frequency predicted by neutral theory model.  pred.lwr and pred.upr, the confidence interval of occurrence frequency estimated by neutral theory model.  bino.pred, bino.lwr, bino.upr, pois.pred, pois.lwr, and bino.upr, the expectation and confidence interval of occurrence frequency estimated by bionomial and Poisson model, respectively.  type, the taxon is identified as 'Neutral', or 'Below' or 'Above' the confidence interval of neutral expectation. |
| `type.uw` | the percentage (unweighted) of taxa within (Neutral), below, or above the confidence interval of the neutral theory expected frequency (given the regional relative abudance). |
| `type.wt` | the abundance weighted percentage (relative abundance sum) of taxa within (Neutral), below, or above the confidence interval of the neutral theory expectation. |
| `sp.names` | the taxa IDs for each type. |

Output of snm.boot is a list.

|  |  |
| --- | --- |
| `stats, detail, type.uw, type.wt` | output only if detail is TRUE. the same as output of snm. |
| `summary` | a matrix, showing observed values, mean, standard deviation, quartiles, boxplot key points and outliers, for the unweighted and weighted precentage of taxa within (Neutral), below, and above the confidence interval of neutral theory expectation. |
| `rand` | a matrix, showing the bootstrapping values of the unweighted and weighted precentage of different types of taxa. Each row represents one time of bootstrapping. |

Output of snm.comm is a list.

|  |  |
| --- | --- |
| `stats` | treat.type, the treatment type, a column name of the input 'treat'. treatment.id, the treatment name. Others are the same as the output 'stats' of snm. The most commonly used information is the dispersal rate values under different treatments, to investigate the effect of treatment on species dispersal. |
| `plot.detail` | a matrix, showing the output 'detail' of snm for each treatment. it is ofen used to show the type of each taxon and draw the figure of neutral confidence interval and each taxon. |
| `ratio.summary` | a matrix, showing the output 'summary' of snm.boot for each treatment. it is ofen used to draw box plots. |
| `pvalues` | a matrix, showing significance of difference between different treatments. |
| `boot.detail` | output only if output.detail is TRUE. A matrix, showing the output 'rand' of snm.boot for each treatment. |

### Note

Version 3: 2020.8.21, update help document, add example.

Version 2: 2018.4.16, add meta.group, meta.com, meta.coms, to consider if the samples are from different metacommunities.

Version 1: 2017.7.21

### Author(s)

Daliang Ning

### References

Burns, A.R., Stephens, W.Z., Stagaman, K., Wong, S., Rawls, J.F., Guillemin, K. et al. (2016). Contribution of neutral processes to the assembly of gut microbial communities in the zebrafish over host development. ISME J, 10, 655-664.

Sloan, W.T., Lunn, M., Woodcock, S., Head, I.M., Nee, S. & Curtis, T.P. (2006). Quantifying the roles of immigration and chance in shaping prokaryote community structure. Environ Microbiol, 8, 732-740.

Sloan, W.T., Woodcock, S., Lunn, M., Head, I.M. & Curtis, T.P. (2007). Modeling taxa-abundance distributions in microbial communities using environmental sequence data. Microbial Ecology, 53, 443-455.

Hubbell, S.P. (2001). The unified neutral theory of biodiversity and biogeography. Princeton University Press, Princeton, New Jersey.

### Examples

```
data("example.data")
comm=example.data$comm
treat=example.data$treat
rand.time=10 # usually use 1000 for real data.
snmtest=snm.comm(comm = comm, treat = treat,
                 rand = rand.time)
```

---

[Package *iCAMP* version 1.2.4 Index]
