# Supplementary material for: A quantitative framework reveals ecological drivers of grassland microbial community assembly in response to warming
Source: Nat Commun. 2020 Sep 18;11:4717. doi: 10.1038/s41467-020-18560-z (PMC7501310; doi:10.1038/s41467-020-18560-z)
Supplement: Supplementary file 5 — Supplementary Code 1 [file 41467_2020_18560_MOESM5_ESM.zip › iCAMP_1.2.4.zip!/iCAMP/html/taxa.binphy.big.html]

R: Phylogenetic binning based on phylogenetic tree

|  |  |
| --- | --- |
| taxa.binphy.big {iCAMP} | R Documentation |

## Phylogenetic binning based on phylogenetic tree

### Description

Phylogenetic binning for iCAMP analysis. To handle large phylogenetic tree, phylogenetic distance matrix should be calculated and saved using the package 'bigmemory' in advance.

### Usage

```
taxa.binphy.big(tree, pd.desc, pd.spname, pd.wd,
                outgroup.tip = NA, outgroup.rm = TRUE,
                d.cut=NULL, ds=0.2, bin.size.limit = 24,
                nworker = 4)
```

### Arguments

|  |  |
| --- | --- |
| `tree` | phylogenetic tree, an object of class "phylo". |
| `pd.desc` | the name of the file to hold the backingfile description of the phylogenetic distance matrix, it is usually "pd.desc" if using default setting in pdist.big function. |
| `pd.spname` | character vector, taxa id in the same rank as the big matrix of phylogenetic distances. |
| `pd.wd` | folder path, where the bigmemmory file of the phylogenetic distance matrix are saved. |
| `outgroup.tip` | a vector of tip names (i.e. OTU IDs) which is in totally different lineage from all other tips, thus can be used as outgroup to root the tree. For example, Archaeal OTUs may be set as outgroup tips when analyzing Bacterial OTUs. Default is NA, means no need to set outgroup tip. |
| `outgroup.rm` | logic, whether to remove the outgroup.tip after the tree is rooted. Default is TRUE. |
| `d.cut` | numeric, the distance from root to the truncating point of the tree. |
| `ds` | numeric, the general threshold of phylogenetic distance within which the phylogenetic signal is significant. default is 0.2. |
| `bin.size.limit` | integer, the minimal requirement of bin size (taxa numer in a bin). Default setting is 24. |
| `nworker` | integer, for parallel computing. Either a character vector of host names on which to run the worker copies of R, or a positive integer (in which case that number of copies is run on localhost). default is 4, means 4 threads will be run. |

### Details

The phylogenetic tree is truncated at a certain phylogenetic distance (as short as necessary) to the root (d.cut), by which all the rest connections between tips (taxa) are lower than a threshold. Within the threshold, phylogenetic signal is generally significant. The taxa derived from the same ancestor after the truncating point are grouped to the same strict bin. Then, each small bin is merged into the bin with the nearest relatives. This procedure is repeated until all merged bins have enough taxa (>= bin.size.limit). Bigmemory (Kane et al 2013) is used to deal with large datasets.

### Value

Output is a list.

|  |  |
| --- | --- |
| `sp.bin` | matrix, rownames are taxa IDs; the first column is strict bin IDs; the second column indicates which strict bin the taxon is merged into; the third column is the final bin IDs. |
| `bin.united.sp` | list, each element is a vector of taxa IDs, indicating the taxa in a final bin (after small bins are merged into nearest large bins). |
| `bin.strict.sp` | list, each element is a vector of taxa ID(s), indicating the taxa in a strict bin (before small bins are merged into large bins). |
| `state.strict` | matrix, status of each strict bin. bin.strict.id, the strict bin ID; bin.strict.taxa.num, taxa number in each strict bin; bin.pd.max, bin.pd.mean, and bin.pd.sd, the maximum, mean, and standard deviation of the pairwise phylogenetic distances in each strict bin. |
| `state.united` | matrix, status of each final bin. bin.united.id.old, the ID of the largest strict bin in each final bin; bin.united.tax.num, taxa number in each final bin; bin.pd.max, bin.pd.mean, and bin.pd.sd, the maximum, mean, and standard deviation of the pairwise phylogenetic distances in each strict bin. |

### Note

Version 2: 2020.8.19, update help document, add example.
Version 1: 2015.12.16

### Author(s)

Daliang Ning

### References

Ning, D., Yuan, M., Wu, L., Zhang, Y., Guo, X., Zhou, X. et al. (2020). A quantitative framework reveals the ecological drivers of grassland soil microbial community assembly in response to warming. bioRxiv. doi:10.1101/2020.02.22.960872 (Nature Communications, final revision).

Kane, M.J., Emerson, J., & Weston, S. (2013). Scalable Strategies for Computing with Massive Data. Journal of Statistical Software, 55(14), 1-19. URL http://www.jstatsoft.org/v55/i14/.

### See Also

`icamp.big`

### Examples

```
data("example.data")
comm=example.data$comm
tree=example.data$tree

# since pd.big need to specify a certain folder,
# the following code is set as 'not run'.
# but you may test the code on your computer
# after change the folder path for 'save.wd'.
## Not run: 
  save.wd="C:/test/" # please change to the folder you want to save the big niche difference matrix.
  nworker=2 # parallel computing thread number
  pd.big=pdist.big(tree = tree, wd=save.wd, nworker = nworker)
  
  ds = 0.2 # setting can be changed to explore the best choice
  
  bin.size.limit = 5 # setting can be changed to explore the best choice.
  # here set as 5 just for the small example dataset.
  # For real data, usually try 12 to 48.
  
  phylobin=taxa.binphy.big(tree = tree, pd.desc = pd.big$pd.file,
                           pd.spname = pd.big$tip.label, pd.wd = pd.big$pd.wd,
                           ds = ds, bin.size.limit = bin.size.limit,
                           nworker = nworker)

## End(Not run)
```

---

[Package *iCAMP* version 1.2.4 Index]
