# Supplementary material for: A quantitative framework reveals ecological drivers of grassland microbial community assembly in response to warming
Source: Nat Commun. 2020 Sep 18;11:4717. doi: 10.1038/s41467-020-18560-z (PMC7501310; doi:10.1038/s41467-020-18560-z)
Supplement: Supplementary file 5 — Supplementary Code 1 [file 41467_2020_18560_MOESM5_ESM.zip › iCAMP_1.2.4.zip!/iCAMP/html/tree.droot.html]

R: Distance from root to tip(s) and node(s) on phylogenetic tree

|  |  |
| --- | --- |
| tree.droot {iCAMP} | R Documentation |

## Distance from root to tip(s) and node(s) on phylogenetic tree

### Description

To calculate the distance from root to tip(s) and node(s) on phylogenetic tree

### Usage

```
tree.droot(tree, range = NA, nworker = 4, output.path = FALSE)
```

### Arguments

|  |  |
| --- | --- |
| `tree` | Phylogenetic tree, an object of class "phylo". |
| `range` | NA or a vector of integer, to specify the numbering of the tips/nodes of which the distances to root will be calculated. The numbering corresponds to those in the element "edge" of the tree. Default is NA, means to calculate all tips and nodes. |
| `nworker` | integer, for parallel computing. Either a character vector of host names on which to run the worker copies of R, or a positive integer (in which case that number of copies is run on localhost). default is 4, means 4 threads will be run. |
| `output.path` | logic, this function will call the function tree.path, if output.path is TRUE, the result of tree.path will be included in the output. Default is FALSE. |

### Details

A tool to get distances to root, used in phylogenetic binning.

### Value

If output.path is FALSE, output is a matrix where the first column indicates the numbering of nodes/tips and the second column has the distance to root.
If output.path is TRUE, output is a list with two elements.

|  |  |
| --- | --- |
| `droot` | matrix, the first column indicates the numbering of nodes/tips and the second column has the distance to root. |
| `path` | result of tree.path, list of nodes and edge lengthes from root to each tip and/or node. |

### Note

Version 2: 2020.8.19, add example.
Version 1: 2015.8.19

### Author(s)

Daliang Ning

### See Also

`tree.path`

### Examples

```
tree=ape::rtree(4)
nworker=2 # parallel computing thread number
droot=tree.droot(tree = tree, nworker = nworker)
```

---

[Package *iCAMP* version 1.2.4 Index]
