# Supplementary material for: A quantitative framework reveals ecological drivers of grassland microbial community assembly in response to warming
Source: Nat Commun. 2020 Sep 18;11:4717. doi: 10.1038/s41467-020-18560-z (PMC7501310; doi:10.1038/s41467-020-18560-z)
Supplement: Supplementary file 5 — Supplementary Code 1 [file 41467_2020_18560_MOESM5_ESM.zip › iCAMP_1.2.4.zip!/iCAMP/html/tree.path.html]

R: List nodes and edge lengthes from root to each tip and/or...

|  |  |
| --- | --- |
| tree.path {iCAMP} | R Documentation |

## List nodes and edge lengthes from root to each tip and/or node

### Description

To list all the nodes and edge lengthes from root to every tip and/or node.

### Usage

```
tree.path(tree, nworker = 4, range = NA, cum = c("no", "from.root", "from.tip", "both"))
```

### Arguments

|  |  |
| --- | --- |
| `tree` | Phylogenetic tree, an object of class "phylo". |
| `nworker` | for parallel computing. Either a character vector of host names on which to run the worker copies of R, or a positive integer (in which case that number of copies is run on localhost). default is 4. |
| `range` | a numeric vector, to specify nodes and/or tips to which the path from root will be calculated. default is NA, means all tips. |
| `cum` | method to calculate cumulative banch length. "no" means not to calculate cumulative lenght; "from.root" means to cumulate from root to tip; "from.tip" means to cumulate from tip to root; "both" means to calculate in both ways and return both results. |

### Details

This function can be useful in phylogenetic diversity analysis, for example, phylogenetic distance, phylogenetic Hill number, phylogenetic binning, etc.

### Value

A list result will be returned.
1st layer (the names of the list) is the end of the path, usually the names of tips and/or nodes
In 2nd layer, [[1]] is the orders of nodes between root and the tip/node specified in 1st layer;
[[2]] is the edge lengthes. if cum="both", [[3]] is cumulative length from root, and [[4]] is cumulative length from tip, otherwise, [[3]] is the cumulative length specified by cum.

### Note

Version 1: 2016.2.14

### Author(s)

Daliang Ning

### See Also

`taxa.binphy.big`,`tree.droot`

### Examples

```
tree=ape::rtree(6)
nworker=2 # parallel computing thread number
tree.path(tree=tree, nworker=nworker, cum="both")
```

---

[Package *iCAMP* version 1.2.4 Index]
